# Evaluating the efficacy of human dental pulp stem cells and scaffold combination for bone regeneration in animal models: a systematic review and meta-analysis

**DOI:** 10.1186/s13287-023-03357-w

**Published:** 2023-05-15

**Authors:** Amin Namjoynik, Md Asiful Islam, Mohammad Islam

**Affiliations:** 1 School of Dentistry, University of Dundee, Dundee, DD1 4HR Scotland, UK; 2 Institute of Metabolism and Systems Research, University of Birmingham, Birmingham, B15 2TT UK

**Keywords:** Systematic review, Meta-analysis, Bone regeneration, Scaffolds, Dental pulp mesenchymal stem cells, animal bone defect model

## Abstract

**Introduction:**

Human adult dental pulp stem cells (hDPSC) and stem cells from human exfoliated deciduous teeth (SHED) hold promise in bone regeneration for their easy accessibility, high proliferation rate, self-renewal and osteogenic differentiation capacity. Various organic and inorganic scaffold materials were pre-seeded with human dental pulp stem cells in animals, with promising outcomes in new bone formation. Nevertheless, the clinical trial for bone regeneration using dental pulp stem cells is still in its infancy. Thus, the aim of this systematic review and meta-analysis is to synthesise the evidence of the efficacy of human dental pulp stem cells and the scaffold combination for bone regeneration in animal bone defect models.

**Methodology:**

This study was registered in PROSPERO (CRD2021274976), and PRISMA guideline was followed to include the relevant full-text papers using exclusion and inclusion criteria. Data were extracted for the systematic review. Quality assessment and the risk of bias were also carried out using the CAMARADES tool. Quantitative bone regeneration data of the experimental (scaffold + hDPSC/SHED) and the control (scaffold-only) groups were also extracted for meta-analysis.

**Results:**

Forty-nine papers were included for systematic review and only 27 of them were qualified for meta-analysis. 90% of the included papers were assessed as medium to low risk. In the meta-analysis, qualified studies were grouped by the unit of bone regeneration measurement. Overall, bone regeneration was significantly higher (*p* < 0.0001) in experimental group (scaffold + hDPSC/SHED) compared to the control group (scaffold-only) (SMD: 1.863, 95% CI 1.121–2.605). However, the effect is almost entirely driven by the % new bone formation group (SMD: 3.929, 95% CI 2.612–5.246) while % BV/TV (SMD: 2.693, 95% CI − 0.001–5.388) shows a marginal effect. Dogs and hydroxyapatite-containing scaffolds have the highest capacity in % new bone formation in response to human DPSC/SHED. The funnel plot exhibits no apparent asymmetry representing a lack of remarkable publication bias. Sensitivity analysis also indicated that the results generated in this meta-analysis are robust and reliable.

**Conclusion:**

This is the first synthesised evidence showing that human DPSCs/SHED and scaffold combination enhanced bone regeneration highly significantly compared to the cell-free scaffold irrespective of scaffold type and animal species used. So, dental pulp stem cells could be a promising tool for treating various bone diseases, and more clinical trials need to be conducted to evaluate the effectiveness of dental pulp stem cell-based therapies.

**Supplementary Information:**

The online version contains supplementary material available at 10.1186/s13287-023-03357-w.

## Introduction

Many orthopaedic and dental complications involve the need for bone grafts, such as repair of traumatic and congenital defects, spinal surgery and build-up of bone stock around biomedical implants. Nevertheless, achieving complete and functional bone regeneration remains major challenge for orthopaedic and craniofacial surgeons. Diverse techniques are currently used in the clinic for bone regeneration, such as bone grafting, distraction osteogenesis and guided bone regeneration (GBR) [1–3]. While autogenous bone grafts are the gold standard for bone regeneration, donor site morbidity and the limited availability of bone volume restrict their practical application in clinical contexts. Thus, xenograft and synthetic biomaterials are widely explored as bone graft substitutes or scaffolds. As the comprehension of bone tissue biology is improving and with the current advances in the development of tissue engineering, mesenchymal stem cell (MSCs) therapy has drawn major interest in enhancing bone tissue reconstruction [4–6].

Mesenchymal stem cells (MSCs) are multi-potent stromal cells with the ability to undergo self-renewal and multi-lineage differentiation. Dental pulp mesenchymal stem cells such as adult dental pulp stem cells (DPSCs) and stem cells from human exfoliated deciduous teeth (SHED) have attracted growing attention due to their high proliferation rate, excellent bone forming potential, and favourable paracrine and immunomodulatory properties [7]. Furthermore, the ease of isolation and accessibility of DPSCs and SHED from removed and discarded teeth offers an abundant source of cells for regenerative medicine with minimal risk of complications, putting them at an advantage over bone marrow and embryonic stem cells [8]. It has been more than twenty years since Gronthos et al*.* [9] coined the term dental pulp stem cells (DPSC) and successfully demonstrated their mesenchymal stem cells (MSCs) properties. DPSCs are members of dental mesenchymal stem cells (DMSCs), which with high multi-lineage differentiation potential, offer an exogenous alternative to osteoblasts and other slow or non-regenerating cells [10]. Also, DPSCs’ capacity to retain stemness after cryopreservation would allow for long-term preservation and upscale production [10]. While SHED is reported to have a higher differentiation yield, it produces an almost equivalent degree of bone regeneration to hDPSC [11]. hDPSCs/SHEDs are already studied in pre-clinical studies for healing of bone-related diseases or surgical interventions that require grafting, included but not limited to implant placement for missing teeth [12], healing of alveolar bone loss by periodontitis [13] and bone fracture [14].

The scaffold, another important component for tissue engineering, facilitates the regenerative process by providing a mechanical supporting network that holds recruited stem cells in place and allows growth factor attachment enabling regeneration. The degree of success of bone regeneration largely depends upon the stem cells and their incorporation with the scaffold materials and recruiting growth factors. Various organic and inorganic scaffold materials have been used in bone regeneration in vitro and in vivo so far, with a varying degree of success depending upon the type of stem cells used and scaffold’s ability to provide stem cells with a compatible home [15].

After 22 years of the first discovery of DPSCs, this is the high time to evaluate the efficacy of DPSCs/SHED on bone regeneration in the in vivo (animal) system to help scientists and clinicians make informed decisions for setting up clinical trials on bone regeneration therapy. This systematic review and meta-analysis aimed to synthesise the evidence of bone regeneration efficacy of DPSCs and SHED pre-seeded with different scaffolds used in animal bone defect models.

## Methodology

### Guidelines and protocol registration

This systematic review and meta-analysis were registered through the international prospective register of systematic reviews (PROSPERO, Registration number—CRD42021274976) following PRISMA 2020 flow diagram and guideline [16].

### Data sources and searches

A customised electronic search of scientific articles was carried out in the PubMed, PubMed–MEDLINE (Ovid), Scopus, EMBASE (Ovid) and Web of Science databases until 30 April 2022 without applying restrictions on the publication date. Articles containing the following keywords (Free text, or, MeSH terms), separately and in combination, were used: ‘Dental Pulp Mesenchymal Stem Cells (Free text), DPSC (Free text), Dental Pulp Stem cells (Free text), SHED (Free text), Stem cells from human exfoliated deciduous teeth (Free text), Bone Regeneration (MeSH term), Bone regenerations (MeSH term), Osteo-regeneration (Free text), Osteoregeneration (Free text), Guided—bone regeneration, Scaffold (MeSH term), Scaffolds (MeSH term), Scaffold Matrix (MeSH term), Scaffold/Matrix (MeSH term), Scaffold for bone regeneration (MeSH term), Scaffolding (MeSH term), Scaffoldings (MeSH term), Bone substitute (MeSH term), Bone substitute material (MeSH term), Bone substitutes (MeSH term), Bone augmentation material (Free text), Alloplastic material, Bone graft, xenograft, Allograft, Ceramics, Autograft’. These keywords were also searched without MeSH in PubMed–MEDLINE. An example of the search strategy is included in Additional file 1. Following this search strategy, all titles and abstracts retrieved were evaluated against the exclusion criteria.

### Eligibility criteria

#### Types of Studies

All studies published in English up to 30 April 2022, which also had been original in vivo (animal) studies using bone defect models, were eligible for this review. Any studies that did not specifically use the keyword ‘scaffold’ yet used a scaffold, bone substitute and bone augmentation materials were also included.

The exclusion criteria were studies that did not contain the search keywords (“Data sources and searches” section), articles written in languages other than English, studies that presented non-original full-text articles, including updates, reviews, systematic reviews, meta-analyses or case reports, studies that did not evaluate the bone regeneration and studies in which dental pulp stem cells were not used. Additionally, any articles that did not have their full text freely accessible were excluded. The review is limited to in vivo studies on animals; hence, ex vivo, in vitro, in silico only and human clinical trials were excluded.

#### Types of participants

All animal varieties/types were included in this review, irrespective of species, sex and age.

Furthermore, the included studies must have used the stem cells from human adult dental pulp (hDPSC) or human exfoliated deciduous teeth (SHED) as a source of human mesenchymal stem cells (hMSCs) for bone regeneration.

#### Types of interventions

Studies with no scaffold were excluded. Studies that used hDPSCs/SHED + scaffold as the experimental group and scaffold-only (cell-free) as the negative control were the primary criteria to be included in the meta-analysis.

#### Outcome measures

Studies that used either % BV/TV or BV (mm^3^) or bone mineral density or BMD (mg/cm^3^) or % bone formation or new bone formation (mm^2^) or osteogenic marker expression or a combination of two or more of the unit to measure the bone regeneration capacity of the DPSCs/SHED incorporated with the scaffolds were included.

### Study selection

Following PRISMA protocol, the inclusion and exclusion criteria were applied in two phases. The initial screening was based on the title and abstract of the articles and performed in Rayyan, the systematic reviews web app (https://www.rayyan.ai/). Also, any duplicated articles were excluded from the review at this stage. This was followed by a full-text screening of the eligible manuscripts for final inclusion, which was performed on EndNote reference management software. In each phase, two researchers conducted assessments independently. Discrepancies were resolved through discussion and consensus between the observers. In addition, reviewers reported the reason for each excluded article, labelled as; bone regeneration, In vitro, human clinical study, no DPSC/SHED, no scaffold, no human DPSCs, DPSCs/SHED not incorporated with the scaffold (cell-free scaffold) as the test sample, use of extracellular vesicles (EVs) and lack of correct characterisation.

### Data extraction process

Qualitative data were extracted by two independent reviewers from the full text of included literature, which was then categorised by the first author, year, scaffold types, stem cells origin (hDPSC or SHED), species of animals, total number of animals, type of bone defects, bone formation evaluation technique, criteria for bone regeneration measurement, the healing period in weeks and the concluding remarks of the included study. Similar to the previous stage, the observers resolved discrepancies through discussion and consensus.

Any relevant quantitative data from the tables, text or figures were also extracted. In case data were not reported or unclear, the authors were contacted by email (maximum two attempts; 2 weeks follow up period after the first message). If an outcome was measured at multiple time points, data from the last time point were included. In cases where data from the authors were not received, they were extracted from graphs using PlotDigitizer software. If any data was presented as SEM, they were converted to SD by multiplying the SEM by the squared root of the N (number of samples). In case of more than 1 type of scaffold is used in any study, scaffolds were labelled as small English letters (a, b, c) in the data extraction table, and the same letters were added after the author’s name and publication year in the meta-analysis corresponding the same scaffolds.

### Quality assessment and risk of bias

The 49 studies included in this review were assessed independently by two reviewers using the modified version of the ‘CAMARADES checklist for study quality [17]. Two components were altered to compensate for blinded implant/insertion of scaffolds (component 3) and the use of anaesthetic on the animal model where necessary throughout the study (component 6). Each ‘yes’ qualified for the score ‘1’, while ‘no’ or ‘unclear response’ carried no weight (i.e. score 0). The risk of each article was judged as ‘high’ for scoring 0 to 3, ‘medium’ for scoring between 4 and 6, or ‘low’ for scoring 7 to 10, according to their total score value (out of 10). For this assessment, the institutions’ names and journal titles were blinded; the only visible identifiers were the first author’s surname and publication year. Also, any discrepancies were resolved via discussion and consensus between the reviewers.

### Data synthesis and statistical analysis

The standardised mean difference (SMD) was calculated with a 95% confidence interval (CI) to estimate overall bone regeneration in the experimental group (dental pulp stem cells + scaffold) compared to the control group (scaffold-only). All the analyses and plots were generated by using comprehensive meta-analysis software.

### Publication bias and heterogeneity

To visually examine publication bias, we constructed a funnel plot displaying the SMD versus standard error. Heterogeneity between studies was assessed using the* I*^2^ statistic (*I*^2^ > 75% indicating substantial heterogeneity) in addition to using Cochran’s Q test to identify the significance of heterogeneity.

### Subgroup and sensitivity analysis

In subgroup analyses, we subgrouped the studies based on the units used and estimated the mean difference (MD) with 95% CI to estimate overall bone regeneration in the experimental group (DPSCs/SHED + scaffold) compared to the control group (scaffold-only). In sensitivity analyses, firstly, we used the leave-one-out method to explore whether any single study has an influence on the main outcome. Secondly, for the meta-analysis, we excluded the high risk of bias studies to observe whether any low-quality study influences the overall outcome. Thirdly, we excluded small studies with less than ten samples to see whether small studies have any effect on the main outcome.

## Results

### Study selection and PRISMA flow diagram

**Fig. 1:**
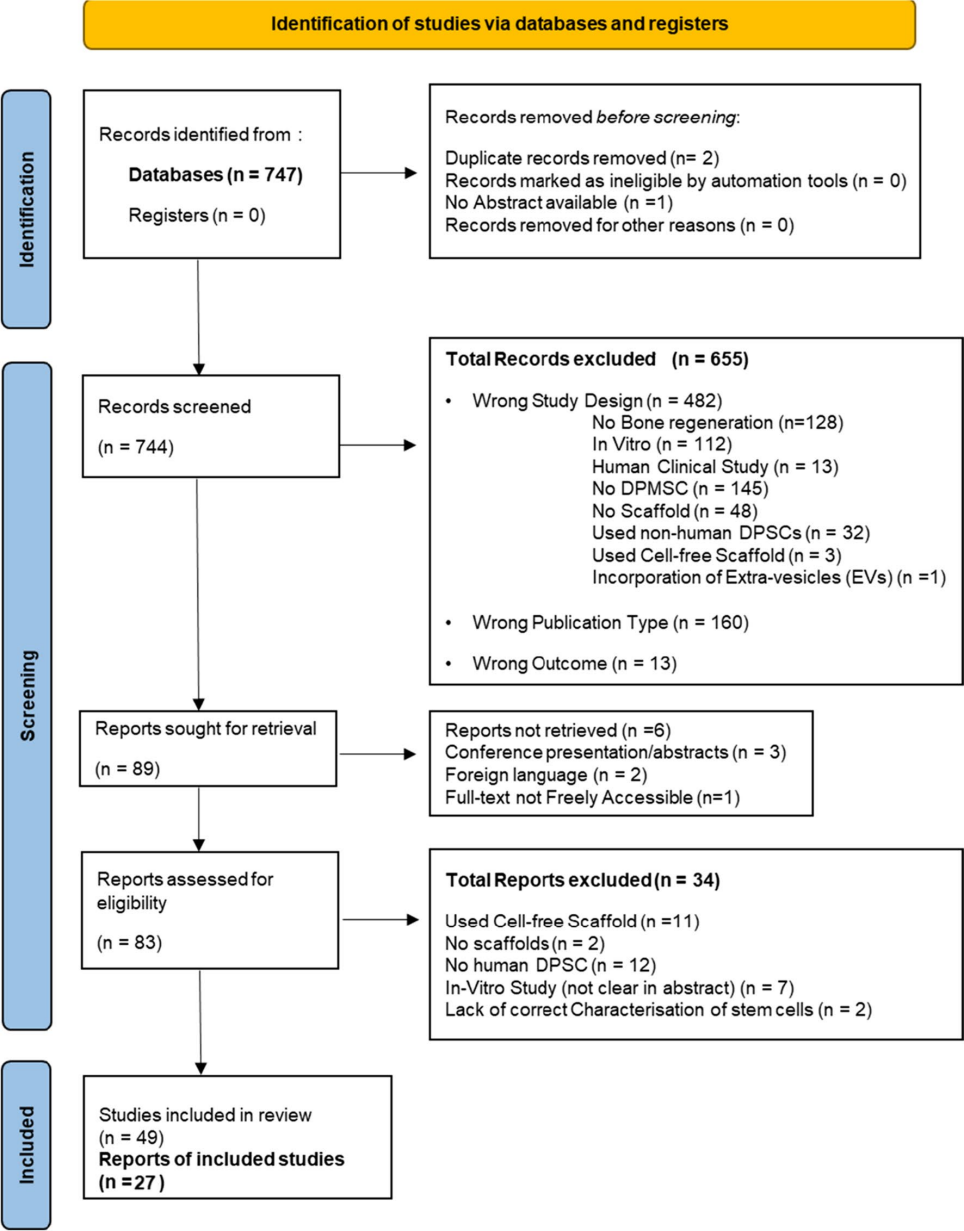
PRISMA flow diagram for systematic review [18]

If any study did not analyse and describe the result of ‘DPSC/SHED + scaffold’ in bone regeneration compared to ‘scaffold-only’, regarded as a ‘wrong outcome’. Studies that are not original full-text articles, including updates, reviews, systematic reviews, meta-analyses or case reports, were regarded as ‘wrong publication type’. Lack of full-text articles was regarded as the ‘reports not retrieved’ and if any study did not use any mesenchymal stem cell characterisation method or report any characterisation result, was regarded as a ‘lack of correct characterisation’. There were 2 phases of screening processes: the first was only abstract screening, from which some articles did not fully meet the exclusion criteria because, for example, it was not clear in the abstract whether they had used any scaffold or whether they were in vitro studies only and required reading of the full text; hence, they were included in the first phase. Some of those articles were excluded later, during the full-text screening phase. This made some of the articles excluded for ‘no scaffold’ and ‘in vitro study’ in the second phase as well (Fig. 1). Forty-nine articles were included in the systematic review after the full-text screening (Table 1).

**Table 1:** Data extracted from included papers in the systematic review

References	Type of scaffolds	Stem cells number/density	Animal species	Number of animals	Bone defect model and follow-up	Bone formation measurement	Scaffold + Cells (Mean ± SD (sample number))	Scaffold-only control (Mean ± SD (sample number))	Concluding remarks
Annibali [19]	GDPB (Bio-Oss) with collagen and ß-TCP	1 × 10^6^ DPSC/defect (scaffold)	NIH-RNU FOXN1 nude rats	8	Critical Size Cranial defect, 12 weeks	Bone mineral density, BMD (mg/cm^3^)	396.93 ± 298.39 (8)	333.38 ± 119.5 (4)	GDPB induces a greater percentage of bone formation as compared to ß-TCP
Annibali [20]	a) GDPB (Bio-Oss) with collagen	1 × 10^6^ DPSC/defect (scaffold)	Fox Chase SCID Beige mice	75	Critical Size Cranial defect, 8 weeks	% BV/TV	17.75 ± 4.8 (4)	21.31 ± 12.95 (3)	Bone regeneration is not significantly increased by DPSCs
	b) ß-TCP						12.5 ± 5.7 (6)	26.52 ± 9.9 (5)	
	c) Agarose/nano-hydroxyapatite						7.29 ± 3.9 (3)	20.08 ± 7.67 (5)	
Ansari [21]	Alginate hydrogel with Cacl2	4 × 10^6^ SHED/defect (scaffold)	C57BL/6 wild mice or Beige nu/nu XIDIII mice	5	Subcutaneous implantation, 8 weeks	% BV/TV	62.8 ± 6.3 (5)	4.2 ± 1.4 (5)	Encapsulated SHED in alginate 100 generated the largest amount of bone formation, while cell-free alginate failed to generate any bone (*p* < 0.05)
Asutay [22]	HA/TCP	DPSC (number not reported)	Albino Wistar rats	15	Calvarial defect, 8 weeks	BMD (mg/cm^3^)	0.40 ± 0.07 (10)	0.24 ± 0.03 (10)	DPSC-loaded-HA/TCP scaffolds demonstrated the potential to benefit of healing process
Bakopoulou [23]	Biomimetic chitosan/gelatin	DPSC (number not reported)	M/SOPF CB17/SCID mice	6	Subcutaneous implantation, 10 weeks	% bone formation	19.77 ± 0.69 (6)	10.3 ± 0.84 (6)	Densely nucleated, nanocrystalline mineralised was greater in the scaffold + DPSC group
Behnia [24]	Cylindrical collagen sponge	SHED (number not reported)	Dog (Iranian mixed breed)	4	Mandibular defect, 12 weeks	% bone formation	75.88 ± 13.12 (4)	45.39 ± 17.91 (4)	SHEDs were capable of proliferation and osteogenesis after 5 years of cryopreservation
Bressan [25]	Hydroxyapatite	1 × 10^7^/ml SHED	Wistar-NIH-FOXN1, nude rat	24	Calvarial defect, 3 weeks	Osteogenic marker expression	Not available	Not available	DPSCs of all donor ages are a potent tool for bone tissue regeneration when mixed with 3D nanostructured scaffolds
Campos [26]	HA and P2O5-CaO-based glass (synthetic bone graft)	1 × 10^5^ DPSC/defect (scaffold)	Merino sheep	12	Mid-diaphysial defect, 120 days	% bone formation	77.5 ± 9.5 (4)	67.8 ± 11.2 (12)	The study proposes bone-like VR and DPSC combination as an efficient binomial strategy
Colorado [27]	Polylactide-co-glycolide/hydroxyapatite (PLGA/HA)	1 × 10^6^ DPSC/defect (scaffold)	Wistar SPF rats	20	Calvarial defect, 10 weeks	New bone formation (mm^2^)	1017.48 ± 24.47 (5)	975.52 ± 35.46 (5)	PLGA/HA scaffolds containing hDPSCs displayed a significant increase in osteoid and mineralised tissue areas, which were superior to that obtained with PLGA/HA scaffolds alone
Colpak [28]	DBBG (deproteinised bovine bone graft) + collagen	2 × 10^6^ DPSC/defect (scaffold)	Healthy sheep	6	Bilateral Iliac defect, 6 weeks	% bone formation	29.00 ± 1.07 (16)	18.45 ± 0.33 (16)	Bone graft and DPMSCs application with dental implant have beneficial effects on newly formed bone and vertical bone height
da Silva [29]	Biphasic calcium phosphate (HA + ß-TCP)	5 × 10^4^ SHED/scaffold initial plating density, cultured for 7 days, then transplanted into the defect	Wistar rats	50	Calvarial defect, 8 weeks	% bone formation	54.38 ± 15.67 (5)	20.1 ± 1.51 (5)	BCP incorporated into SHED cultures showed promising outcomes for the repair of rat calvarial defects
Fahimipour [30]	Collagen-heparin-ß-TCP	5 × 10^6^ DPSC/defect (scaffold)	Fischer 344 rats	15	Subcutaneous implantation, 8 weeks	Osteogenic marker expression	Not available	Not available	The designed construct induced the ectopic bone formation
Fang [31]	Collagen	5 × 10^5^ SHED/scaffold initial plating density, cultured for 7 days, then transplanted into the defect	Sprague–Dawley rats	6	Calvariae cranial defects, 8 weeks	New bone formation (mm^2^)	4.684 ± 0.812 (2)	2.545 ± 0.704 (2)	Collagen + DPSC provides feasibility for clinical trials of large-scale bone loss
Fu [32]	Mineralised gelatin sponge	1 × 10^6^ DPSC/ml initial plating density, cultured for 7 days, then transplanted into the defect	Nude mice	2	Subcutaneous implantation, 7 weeks	Osteogenic marker expression	Not available	Not available	The combination of DPSCs and Gelatin sponge scaffold has a great potential for bone tissue engineering
Ghavimi [33]	Pluronic F68-containing aspirin-loaded PLGA nanoparticles	DPSC (number not reported)	Mongrel dogs	6	Alveolar defect, 4 weeks	New bone formation	Not available	Not available	The prepared membrane can be used as the GBR membrane for bone regeneration and antibacterial effect
Gonçalves [34]	a) PLLA/collagen/HA	1 × 10^6^ SHED/scaffold initial plating density, cultured for 24 h, then transplanted into the defect	Wistar rats	18	Periodontal Fenestration defect, 30 days	New bone formation (mm^2^)	0.27 ± 0.09 (6)	0.28 ± 0.09 (6)	Both materials, even in the absence of stem cells, was able to promote bone and periodontal regeneration
	b) PisPLLA/collagen/HA						0.22 ± 0.07 (6)	0.28 ± 0.09 (6)	
Gutiérrez-Quintero [35]	Hydroxy apatite matrix and polylactic polyglycolic acid (HA/PLGA)	5 × 10^5^ DPSC/scaffold initial plating density, cultured for 24 h, then transplanted into the defect	New Zealand albino male rabbits	8	Mandibular critical-sized defects, 4 weeks	New bone formation (mm)	5.59 ± 2.31 (8)	3.15 ± 1.75 (8)	DPSCs seem to provide osteogenic properties showing significant results in bone regeneration compared with HA/PLGA scaffold
Hiraki [36]	Atelocollagen	1 × 10^5^ SHED/defect (scaffold)	BALB/c-nu mice	18	Calvarial defect, 6 weeks	bone volume (mm^3^)	5.152 ± 1.77 (6)	1.722 ± 0.73 (6)	Bone regeneration was enhanced in defects treated with stem cells compared to that in controls
Huang [37]	HNTs/GelMA hydrogels: halloysite nanotubes (HNTs) + gelatin methacrylate	2 × 10^5^ DPSC/scaffold initial plating density, cultured for 24 h, then transplanted into the defect	Sprague − Dawley rats	12	Calvarial defect, 12 weeks	BMD (mg/cm^3^)	377.15 ± 46.35 (2)	94.4 ± 26.3 (2)	The HNT-incorporated hydrogel proved a promising alternative strategy for bone regeneration
Jahanbin [38]	Collagen matrix	1 × 10^6^ DPSC/scaffold initial plating density, cultured for 24 h, then transplanted into the defect	Wistar rats	60	Maxillary alveolar defect, 8 weeks	% bone formation	27.3 (11)	58.3 (12)	Stem cells plus scaffold have regenerative potential for repairing maxillary alveolar defects
Jin [39]	Puramatrix (Synthetic peptide hydrogel)	1 × 10^6^/ml DPSC	Rats	15	Mandibular bone defect, 6 weeks	% BV/TV	26.17 ± 3.6 (5)	9.62 ± 2.94 (5)	Regenerated bone area of the DPSC + scaffold group was significantly higher than those in the control group
Kang [40]	HA-TCPs, demineralised dentin matrix (DDM)	1 × 10^6^ DPSC/defect (scaffold)	Nude (athymic) mice	20	Subcutaneous implantation, 8 weeks	Bone volume change (mm3)	1.2 ± 1.4 (5)	− 0.819 (5)	Both HA-TCP and DDM induced in vitro osteogenic differentiation potential of hDPSCs transplanted, and they enhanced ectopic bone tissue formation
Kawanabe [41]	ß-TCP	2 × 10^6^ DPSC/defect (scaffold)	Fox Chase SCID mice	N/A	Subcutaneous implantation, 8 weeks	Osteogenic marker expression	Not available	Not available	Transplanted βTCP scaffolds and the specific cell surface antigen, SSEA-4 + DPSC generated a bone-like structure
Kunwong [42]	PLGA-10% bioactive glass	SHED (number not reported)	Sprague–Dawley rats	N/A	Cleft mimicking model, 180 days	Osteogenic marker expression	Not available	Not available	SHED-PLGA-10% bioactive glass transplantation group showed more bone matrix than PLGA-10% bioactive glass without cells
Kuo [43]	a) Calcium sulphate dehydrate (CSD)	2 × 10^6^/ml DPSC	Lanyu swine	12	Mandibular bone defect, 8 weeks	% bone formation	69.7 ± 4.9 (3)	33.9 ± 9.9 (3)	Mixing hDPSCs into the pure CSD showed effective improvement in new bone regeneration comparing to α-CSH/ACP or CSD/β-TCP
	b) α-calcium sulphate hemihydrate/amorphous calcium phosphate (α-CSH/ACP)						70.5 ± 6.6 (3)	61.7 ± 2.3 (3)	
	c) CSD/β tricalcium phosphates (β-TCP)						57.1 ± 4.1 (3)	44.5 ± 2.9 (3)	
Kwon [44]	PLGC co-polymer scaffold: (MPEG-(PLLA-co-PGA-co-PCL) (PLGC))	1 × 10^6^ DPSC/defect (scaffold)	Sprague–Dawley rats	30	Cranial defect, 12 weeks	% bone formation	53 ± 6.7 (5)	6 ± 2.1 (5)	The defect area in the PLGC scaffold/hDPSCs group was replaced by neo-bone tissues
Liu [45]	HA + ß-TCP	6 × 10^6^ SHED/defect (scaffold)	C57BL/6 J mice and Beige nude/nude Xid (III) mice	N/A	Subcutaneous implantation, 8 weeks	% bone formation	Not applicable	Not applicable	Effect of Acetyl Salicylic Acid (ASA) only was analysed. When HA/TCP implanted with low doses of ASA (10/50 μg/mL) treatment, SHED-mediated new bone regeneration was increased
Man [46]	3D silk fibroin	DPSC (number not reported)	CD1 nude mice	N/A	Subcutaneous implantation, 6 weeks	Osteogenic marker expression	Not available	Not available	Selective HDAC2 and 3 inhibitor MI192 can promote hDPSCs osteogenic differentiation within lyophilised Bombyx Mori silk scaffolds
Maraldi [47]	Collagen sponge	DPSC (number not reported)	CD® IG5 rats	30	Cranial defect, 8 weeks	% bone formation	57.32 ± 3.99 (5)	43.8 ± 7 (5)	Cell seeded group showed significantly higher mineralised tissue in the defect area than the cell-free group
Mohanram [48]	Natural HA (anorganic bone mineral—ABM)	5 × 10^6^ DPSC/defect (scaffold)	MF1 Nu/Nu mice	4	Intraperitoneal chamber diffusion model, 8 weeks	Osteogenic marker expression	Not available	Not available	ABM-P-15 (collagen peptide) promoted HDPSCs osteogenic differentiation and bone matrix formation
Nakajima [11]	PLGA membrane	SHED (number not reported)	BLAB/c-nu mice	20	Calvarial defect, 12 weeks	% BV/TV	27.1 ± 12.13 (5)	8.91 ± 6.5 (2) Empty ctrl	SHED may be one of the best cell source candidates for reconstructing an alveolar cleft
Niu [49]	Intrafibrillar-silicified collagen scaffolds (ISCS)	5 × 10^6^ DPSC/ml initial plating density, cultured for 2 weeks, then transplanted into the defect	Nude mice	6	Subcutaneous implantation, 8 weeks	Osteogenic marker expression	Not available	Not available	Intrafibrillar-silicified collagen scaffolds significantly promoted the proliferation, osteogenic differentiation and mineralisation of hDPSCs, when compared with control
Novais [50]	3D Collagen	SHED (number not reported)	Athymic (nude) ‘NMRI-Foxn1 nu/nu’ mice	45	Calvarial defect, 90 days	% BV/TV	Not applicable	Not applicable	Effect of hypoxia and FGF2 only analysed and discussed and scaffold + cells were used as control. Priming SHED with FGF-2 in compressed collagen greatly enhanced regeneration
Petridis [51]	Hydrogel scaffold (HyStem™-HP Cell Scaffold Kit, Sigma-Aldrich), composed by hyaluronic acid, heparin sulphate, gelatin and PEDGA solution	1 × 10^6^ DPSC/defect (scaffold)	Wistar rats	30	Calvarial defect, 8 weeks	% bone formation	32.78 ± 9.24 (17)	24.40 ± 8.29 (13)	The per cent of new bone formation in the cell–scaffold-treated group was significantly higher compared to scaffold treated groups
Pisciotta [52]	Collagen sponge	1 × 10^6^ DPSC/scaffold initial plating density, cultured for 10 days, then transplanted into the defect	Sprague–Dawley rats	10	Cranial defect, 6 weeks	% bone formation	69.15 ± 7.87 (4)	39.15 ± 4.89 (4)	Stem cell–scaffold constructs, showed a significant contribution to the regeneration of critical size bone defect
Prabha [53]	Polyvinyl alcohol (PVA)-Poly carbolactone (PCL)—hydroxyapatite-based (HAB) scaffold	5 × 10^5^ DPSC/defect (scaffold)	NOD.CB17‐Prkdcscid/J mice	2	Subcutaneous implantation, 8 weeks	Osteogenic marker expression	Not available	Not available	PVA-PCL-HAB scaffold supported the growth and attachment of DPSCs and in vivo vascularised bone formation
Prahasanti [54]	Carbonate apatite scaffold (CAS) + gelatin	1 × 10^6^ SHED/defect (scaffold)	Wistar rats	14	Alveolar defect, 1 week	Osteogenic marker expression	Not available	Not available	SHED-incorporated CAS can enhance BMP-2 and BMP-7 expression while attenuating MMP-8 expression
Prahasanti [55]	Hydroxyapatite	1 × 10^6^ SHED/defect (scaffold)	Wistar rats	14	Alveolar defect, 8 weeks	Osteogenic marker expression	Not available	Not available	Hydroxyapatite scaffold and SHED increase osteoprotegerin expression
Saha [56]	Self-assembling β-peptides (SAPs), P11-4	5 × 10^4^ DPSC/defect (scaffold)	Athymic rats	20	Calvarial defect, 6 weeks	BMD (mg/cm^3^)	871 ± 34.2 (6)	920 ± 71.4 (4)	Repair of the defect was not enhanced by the addition of hDPSCs with P11-4
Salgado [57]	Collagen–nanohydroxy apatite–phophoserine	3 × 10^5^ DPSC/scaffold initial plating density, cultured for 24 h, then transplanted into the defect	Nude mice	4	Subcutaneous implantation, 8 weeks	% bone formation	46.97 ± 3.51 (4)	43.21 ± 3.26 (4)	DPSC enhanced the percentage of the bone formation, but not statistically different to the control
Saskianti [13]	HAS (biohydrox hydroxyapatite)	1 × 10^6^ SHED/defect (scaffold)	Wistar rats	10	Alveolar defect, 1 week	Osteogenic marker expression	Not available	Not available	The expression of VEGF increases significantly and MMP8 expression decreases with treatment of SHED seeded in HAS
Saskianti [58]	Carbonate apatite	1 × 10^6^ SHED/ml initial plating density, cultured for 3 days, then transplanted into the defect	Rats (Rattus norvegicus)	8	Alveolar defect, 3 weeks	Osteogenic marker expression	Not available	Not available	The transplantation of SHED and carbonate apatite increased BMP4 expression as an indicator of osteogenic differentiation
Seo [59]	HA + TCP	2 × 10^6^ SHED/defect (scaffold)	NIH-bg-nu-xid, Harlan Sprague–Dawley mice	18	Calvarial defect 8 weeks	% bone formation	33.7 ± 6.3 (6)	1.24 ± 0.1 (6)	SHED may select unique mechanisms to exert osteogenesis
Serano-Bello [60]	Hydroxyapatite–microporous alginate sponges (MAS)	DPSC (number not reported)	Wistar rats	24	Calvarial defect, 90 days	% bone formation	90 ± 5.88 (6)	3.43 ± 0.35 (6), Empty ctrl	MAS with 30% HA, the total volume of the regenerated area was statistically significant with regard to the control and other groups
Vater [61]	Mineralised collagen Matrix (MCM)	5 × 10^4^ DPSC/scaffold initial plating density, cultured for 24 h, then transplanted into the defect	NMRI nude mice	36	Critical mid-diaphyseal defect, 6 weeks	BMD (mg/cm^3^)	825.5 ± 64 (12)	849.8 ± 43.94 (11)	Pre-seeding of MCM scaffolds with DPSCs did not enhance bone defect healing when compared with the cell-free MCM control
Wongsupa [62]	poly(ε-caprolactone)-biphasic calcium phosphate construct (PCL/BCP)	DPSC (number not reported)	New Zealand white rabbits	18	Calvarial defect, 8 weeks	% BV/TV	25.33 ± 0.75 (3)	13.28 ± 2.41 (3)	hDPSCs combined with PCL/BCP scaffolds may be an augmentation material for bony defect
Xavier Acasigua [63]	poly (lactic-co-glycolic acid) (PLGA)	5 × 10^4^ SHED/scaffold initial plating density, cultured for 14 days, then transplanted into the defect	Wistar rats	20	Calvarial defect, 60 days	% bone formation	17 ± 4.31 (5)	9.39 ± 2.55 (5)	PLGA associated with SHED can promote bone formation
Zhang [64]	Tyrosine-derived polycarbonate, E1001(1 K)/ß-TCP	2.5 × 10^4^ DPSC/mm3 initial plating density, cultured for 7 days, then transplanted into the defect	New Zealand White rabbits	10	Mandibular defect, 90 days	BMD (mg/cm^3^)	0.51 ± 0.1 (3)	0.40 ± 0.1 (2)	Vascularised craniofacial bone was regenerated using hDPSCs combined with the scaffolds
Zhu [65]	Bio-Oss—Collagen	2 × 10^7^ DPSC/ml initial plating density, cultured for 7 days, then transplanted into the defect	Nude mice	36	Calvarial defect, 8 weeks	% BV/TV	56.42 ± 2.62 (9)	47.36 ± 2.41 (9)	It was hypothesised that DPSCs implanted scaffold would promote bone healing in bone defect

### Study characteristics

Biocompatibilities of the scaffold materials were confirmed by in vitro studies in all the included studies. Studies were included only in which dental pulp stem cells were characterised properly in vitro before implanting in the in vivo animal model. Six different animal species were used: the most used species was rat (23 articles), followed by mice (18 articles), rabbit (3 articles), dog (2 articles), sheep (2 articles) and swine (1 article). Various bone defect and bone regeneration models were used, including different sizes of calvarial bone defects (16 articles), subcutaneous implantation (11 articles), alveolar bone defect (6 articles), cranial defect (5 articles), mandibular defect (5 articles), mid-diaphyseal defect (2 articles) and iliac defect, periodontal fenestration defect, cleft-mimicking defect and intraperitoneal diffusion model, 1 article each. hDPSCs were used in 33 articles, and SHEDs were used in 16 articles in the range of 5 × 10^4^–2 × 10^7^ initial transplantation number. Various time frames were used to observe the bone regeneration capacity of the scaffold + stem cell groups in various animal models. Most commonly, 8 weeks was used as the endpoint to analyse the potential of the scaffold for bone regeneration (21 articles) (Table 1).

Different units for outcome measures (bone regeneration) were used in different studies, such as % BV/TV, BV (mm^3^), BMD (mg/cm^3^), % bone formation, new bone formation (mm^2^) and osteogenic marker expression. The reliability of a method to measure % BV/TV, BV (mm^3^), BMD (mg/cm^3^), % bone formation and new bone formation (mm^2^) depends on several factors, including the type of measurement being performed, the equipment being used and the experience of the operator. Micro-CT scanning is a common method for measuring % BV/TV, BV (mm^3^), BMD (mg/cm^3^) and other bone regeneration parameters. Micro-CT scanners can provide highly accurate and precise measurements, but the quality of the results can depend on the resolution of the scanner, the type of sample being measured and the experience of the operator. Histomorphometry is a method for measuring % bone formation and new bone formation (mm^2^) in bone tissue formation. This involves staining and examining thin sections of bone tissue under  a microscope. The accuracy and precision of histomorphometric measurements can depend on the quality of the staining and the experience of the operator.

It is possible to estimate the volume/density/percentage of truly formed bone or bone-like tissues by DPSC/SHEDs application by both micro-CT and histological or histomorphometric analysis. All the included papers used any one or both techniques to estimate the newly formed bone. In the micro-CT analysis, black-and-white tomogram images can be converted into equal density pseudocolour images and the boundary between bone/residual graft can be calibrated. For example, Zhu et al. [65] defined tissues with CT values between 700 and 2000 Hounsfield unit (Hu) as the new bone. Tissues with CT values more than 2000 Hu were defined as the residual graft/scaffold after calibration. In histology or histomorphometric analysis, bone or bone-like tissues and residual graft/scaffold are distinguished and quantified by applying suitable staining reagents such as Masson’s Trichrome and related image analysis software, respectively. The volume/density/percentage of new bone formation by DPSCs/SHEDs in both analyses can be calculated by subtracting the residual graft/scaffold from the total defect area.

**Fig. 2:**
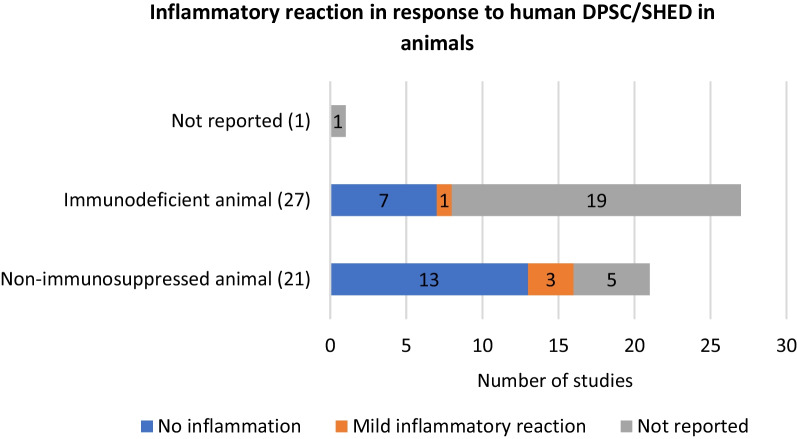
Inflammatory reaction in response to human DPSC and SHED in animals

It has been suggested that the mesenchymal stem cells (MSCs) such as BMSC, DPSC and SHED have an immunomodulatory effect as well as reducing the reaction of the transplant onto the host. Human MSCs can secrete bioactive factors that can inhibit T-cells which helps to establish a regenerative microenvironment in the defect area [66, 67]. Based on this concept, 21 studies in this systematic review used non-immunosuppressed animals-13 of them reported no inflammatory reactions, 3 of them reported mild inflammatory reaction and 5 of them did not report information on the inflammatory reaction. On the other hand, 27 studies used immunodeficient animals-7 of them reported no inflammatory reactions, only 1 study reported mild inflammation in the defect site and 19 studies did not report the information on the inflammatory reaction (Fig. 2).

Detailed information on the inflammatory reaction in response to human DPSCs/SHED in animal defects is listed in Additional file 2.

Due to the lack of quantitative data such as for osteogenic marker expression [13, 25, 30, 32, 41, 42, 46, 48, 49, 53–55, 58] and new bone formation [33], lack of ‘scaffold-only negative control’ data [11, 45, 50, 60], missing SD/SEM [38, 40] and less than 2 articles for each outcome measure [35, 36], only 27 articles out of 49 were qualified in the meta-analysis. Detailed reasons of why 22 articles were excluded from the meta-analyses can be found in Additional file 3. In the meta-analysis, only 4 different outcome measures (unit for bone regeneration) were qualified, most commonly % bone formation (13 articles, 15 test conditions), followed by bone mineral density (mg/cm^3^) (6 articles/test conditions), % BV/TV (5 articles, 7 test conditions) and new bone formation (mm^2^) (3 articles, 4 test conditions).

### Quality assessment and risk of bias

Assessment of risk of bias and quality for included studies were assessed using the CAMARADES tool and listed in Table 2. Only 4 studies out of 49 (8%) reported sample size calculations. 29% of the included studies (14 studies) also reported randomisation of the experimental and control group allocation. Only 3 studies (6%) reported the blinded implantation or insertion of the experimental and control group. However, 76% of included studies did not assess the outcome blindly or failed to report the blinded assessment. In conclusion, 20% of studies were scored as low risk, 70% were at medium risk, and only 10% were scored as high risk of bias (Table 3).

**Table 2:** The Collaborative Approach to Meta-Analysis and Review of Animal Data from Experimental Studies (CAMARADES) tool (released in 2004)

Major components	Response options		
1. Sample size calculation	Yes	No	Unclear
2. Random allocation to treatment or control	Yes	No	Unclear
3. Blinded implant/insertion of scaffold*	Yes	No	Unclear
4. Blinded assessment of outcome	Yes	No	Unclear
5. Appropriate animal defect model	Yes	No	Unclear
6. Use of anaesthetic on animal model where necessary throughout the study*	Yes	No	Unclear
7. Statement of control of temperature*	Yes	No	Unclear
8. Compliance with animal welfare regulations	Yes	No	Unclear
9. Peer-reviewed publication	Yes	No	Unclear
10. Statement of potential conflict of interests	Yes	No	Unclear

*can be modified by user when using in another animal models

Yes = 1 score

No and unclear = 0 score

Total scores (out of 10): Quality

7 to 10: low risk

4 to 6: medium risk

1 to 3: high risk

**Table 3:** Quality assessment of the included studies

References	1	2	3	4	5	6	7	8	9	10	Score	Quality
Annibali [19]	0	0	0	0	1	1	0	1	1	0	4	Medium risk
Annibali [20]	0	1	1	1	1	1	0	1	1	1	8	Low risk
Ansari [21]	0	0	0	0	1	0	0	1	1	0	3	High risk
Asutay [22]	0	0	0	1	1	1	1	1	1	1	7	Low risk
Bakopoulou [23]	1	0	0	1	1	1	0	1	1	0	6	Medium risk
Behnia [24]	0	0	0	1	1	1	1	1	1	0	6	Medium risk
Bressan [25]	0	0	0	0	1	1	1	1	1	1	6	Medium risk
Campos [26]	0	0	0	0	1	1	0	1	1	1	5	Medium risk
Colorado [27]	0	0	0	1	1	1	1	1	1	0	6	Medium risk
Colpak [28]	0	0	0	0	1	1	0	1	1	1	5	Medium risk
da Silva [29]	1	1	0	1	1	1	1	1	1	1	9	Low risk
Fahimipour [30]	0	0	0	0	1	0	1	1	1	1	5	Medium risk
Fang [31]	0	0	0	0	1	1	0	1	1	1	5	Medium risk
Fu [32]	0	0	0	0	1	0	0	0	1	1	3	High risk
Ghavimi [33]	0	0	0	0	1	1	0	1	1	1	5	Medium risk
Gonçalves [34]	0	0	0	0	1	1	0	1	1	1	5	Medium risk
Gutiérrez-Quintero [35]	0	1	0	0	1	1	0	1	1	1	6	Medium risk
Hiraki [36]	0	0	0	0	1	1	0	1	1	1	5	Medium risk
Huang [37]	0	0	0	0	1	1	0	1	1	1	5	Medium risk
Jahanbin [38]	0	1	0	0	1	1	1	0	1	0	5	Medium risk
Jin [39]	0	1	0	0	1	1	0	1	1	1	6	Medium risk
Kang [40]	0	0	0	1	1	1	0	1	1	1	6	Medium risk
Kawanabe [41]	0	0	0	0	1	0	0	0	1	0	2	High risk
Kunwong [42]	0	0	0	0	1	0	0	1	1	1	4	Medium risk
Kuo [43]	0	0	0	0	1	1	0	1	1	0	4	Medium risk
Kwon [44]	0	1	0	0	1	1	0	1	1	1	6	Medium risk
Liu [45]	0	0	0	0	0	0	0	1	1	1	3	High risk
Man [46]	0	0	0	0	1	1	0	1	1	1	5	Medium risk
Maraldi [47]	0	0	0	0	1	1	0	1	1	1	5	Medium risk
Mohanram [48]	0	0	0	0	1	0	0	1	1	1	4	Medium risk
Nakajima [11]	0	0	0	0	1	1	0	1	1	1	5	Medium risk
Niu [49]	0	0	0	0	1	1	0	1	1	0	4	Medium risk
Novais [50]	0	1	0	0	1	1	1	1	1	1	7	Low risk
Petridis [51]	0	0	1	1	1	1	1	1	1	1	8	Low risk
Pisciotta [52]	0	0	0	0	1	0	0	1	1	1	4	Medium risk
Prabha [53]	0	0	0	0	1	1	0	1	1	1	5	Medium risk
Prahasanti [54]	0	1	1	0	1	0	1	1	1	1	7	Low risk
Prahasanti [55]	0	1	0	0	1	1	0	1	1	1	6	Medium risk
Saha [56]	0	0	0	1	1	1	0	1	1	1	6	Medium risk
Salgado [57]	0	0	0	0	1	1	0	1	1	1	5	Medium risk
Saskianti [13]	1	1	0	1	1	1	1	1	1	1	9	Low risk
Saskianti [58]	0	0	0	0	1	0	0	1	1	0	3	High risk
Seo [59]	0	0	0	0	1	1	0	1	1	0	4	Medium risk
Serano-Bello [60]	1	1	0	0	1	1	1	1	1	1	8	Low risk
Vater [61]	0	1	0	1	1	1	0	1	1	1	7	Low risk
Wongsupa [62]	0	1	0	0	1	1	0	1	1	1	6	Medium risk
Xavier Acasigua [63]	0	0	0	0	1	1	0	1	1	1	5	Medium risk
Zhang [64]	0	0	0	0	1	1	0	1	1	1	5	Medium risk
Zhu [65]	0	1	0	1	1	1	0	1	1	1	7	Low risk

### Overall effect by outcome measurement unit

**Fig. 3:**
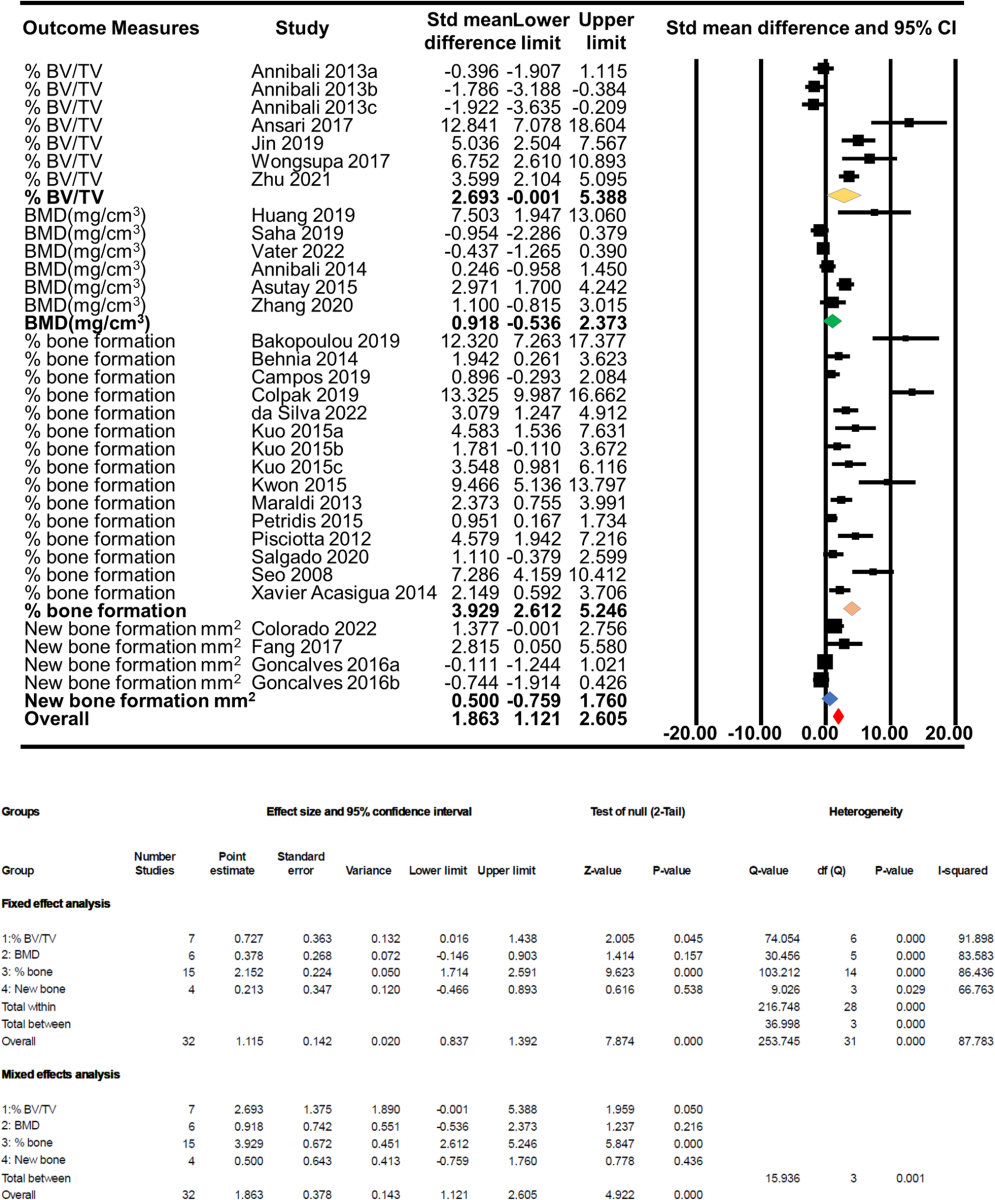
Overall effect by bone regeneration measurement unit

Overall, bone regeneration was significantly higher (*p* < 0.0001) in experimental group (dental pulp stem cells + scaffold) compared to control group (scaffold-only) (SMD: 1.863, 95% CI 1.121–2.605). The effect is 1.863, representing quite a large effect where the experimental group tends to have larger scores than the control group. However, the effect is almost entirely driven by the % bone formation group (SMD: 3.929, 95% CI 2.612–5.246) while %BV/TV (SMD: 2.693, 95% CI − 0.001–5.388) shows a marginal effect and both BMD (SMD: 0.918, 95% CI − 0.536–2.373) and new bone formation (mm^2^) (SMD: 0.500, 95% CI − 0.759–1.760) shows no effects. % bone formation group shows a highly significant effect (*p* < 0.0001) where scaffold + dental pulp stem cells group regenerate bone more than the scaffold-only control group, and there is a significant difference between the groups (*p* < 0.0001). % BV/TV group also shows a marginally significant (*p* = 0.045) effect on bone regeneration by the scaffold + dental pulp stem cells group compared to the scaffold-only group and has a significant difference between the groups (*p* = 0.05) (Fig. 3).

### Subgroup analysis by outcome measurement unit

The amounts of bone defect at t0 and new bone formation at t1 were defined as total volume (TV) and bone volume (BV), respectively. The bone regeneration rate was determined as a percentage of BV/TV using the following formula.

$${\text{Regenerated bone rate }}\left( {\% {\text{BV}}/{\text{TV}}} \right) \, = \frac{{{\text{Regenerated bone volume at t1 }}\left( {{\text{BV}}} \right) \, *}}{{{\text{Bone defect volume at t}}0 \, \left( {{\text{TV}}} \right)}} \times { 1}00$$

*$${\text{Regenerated bone volume at t1 }} = {\text{ Bone defect volume at t}}0 \, - {\text{ Bone defect volume at t1}}$$.

**Fig. 4:**
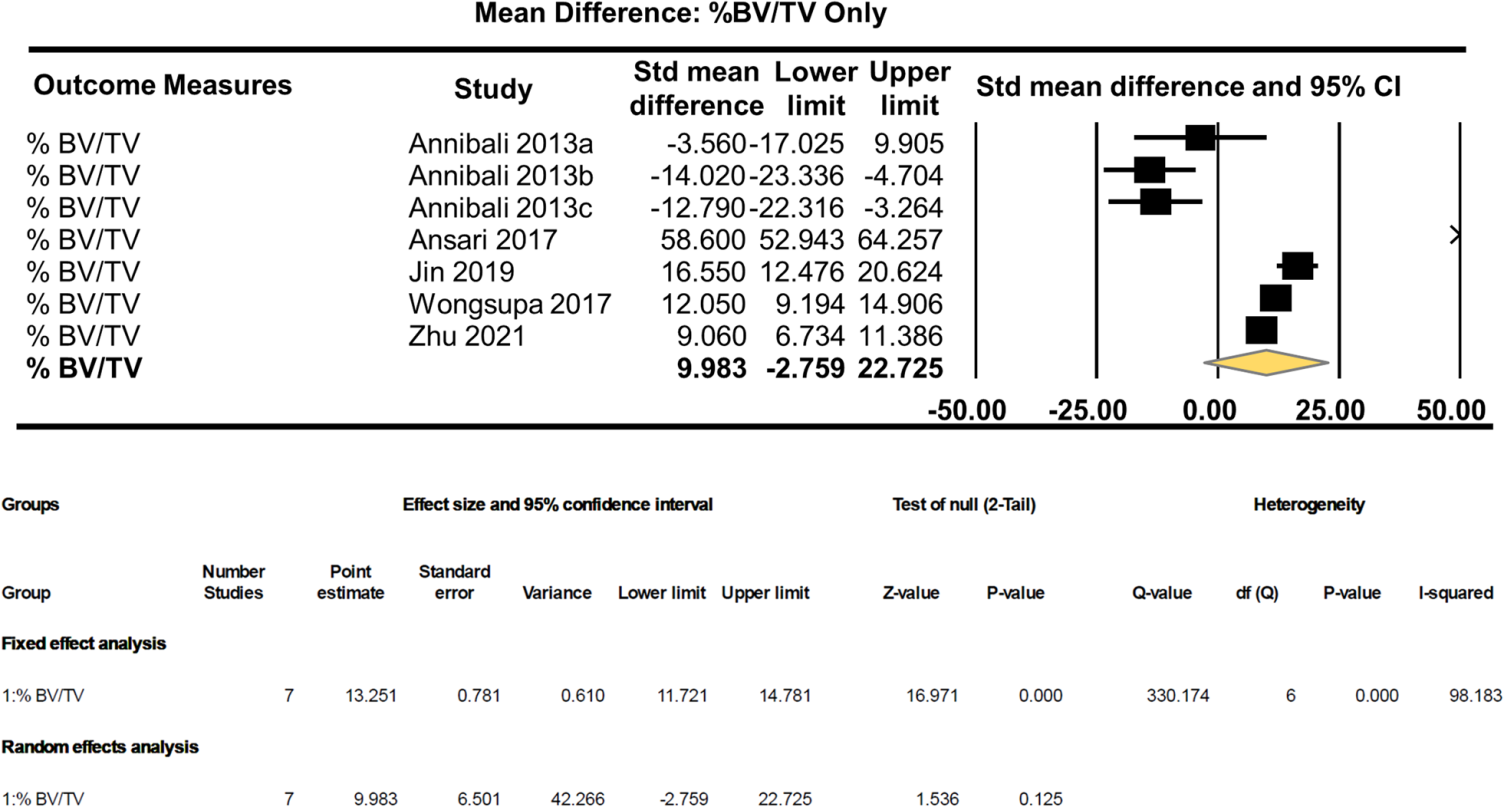
Subgroup effect analysis of the % BV/TV group

Unstandardised random effect analysis of the % BV/TV group alone shows no significant effects in bone regeneration by the scaffold + dental pulp stem cells (MD: 9.983, 95% CI − 2.759–22.725, *p* = 0.125) (Fig. 4).

**Fig. 5:**
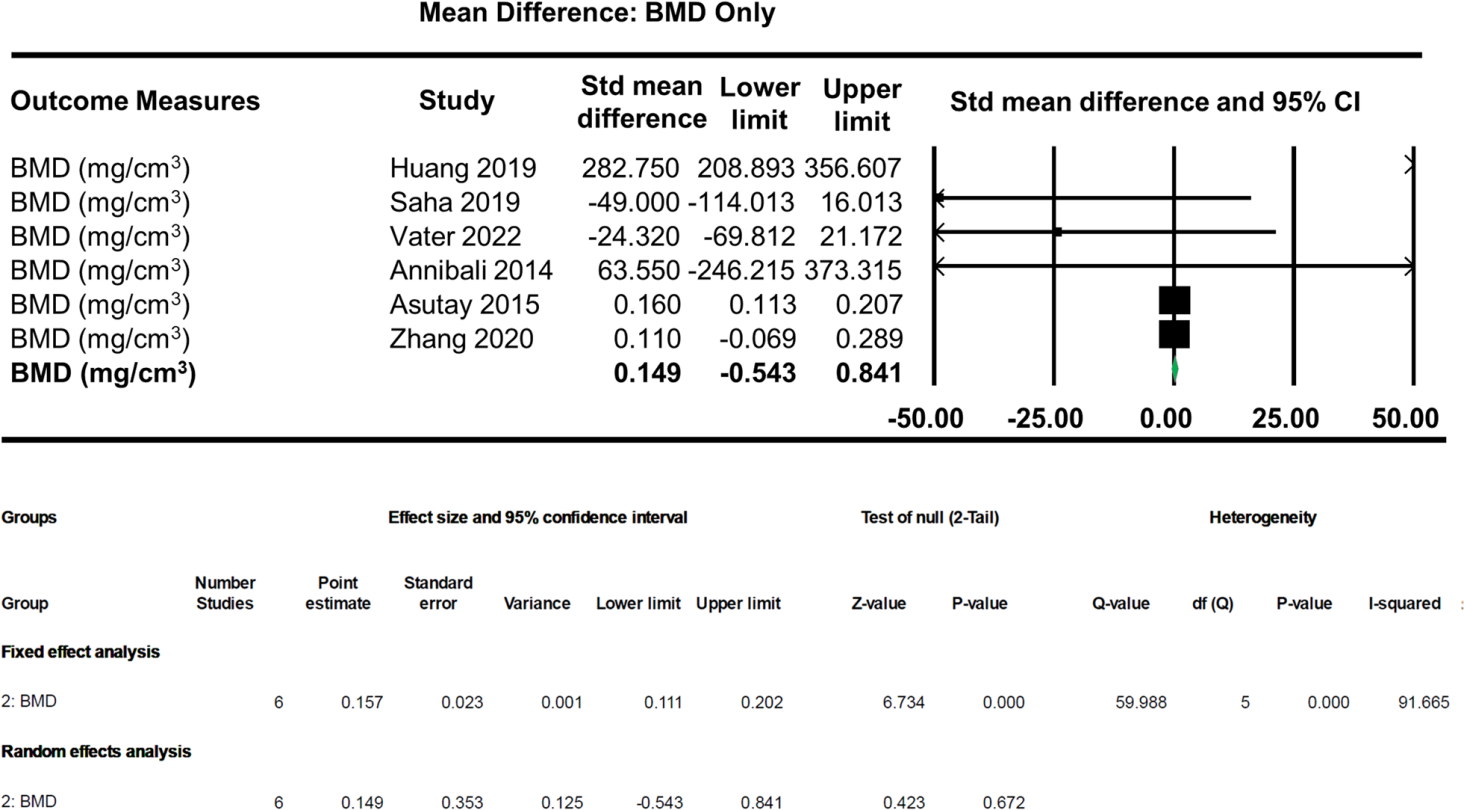
Subgroup effect analysis of the bone mineral density (mg/cm^3^) group

Bone tissue density/bone mineral density is the amount of bone mineral in bone tissue. The BMD values were normalised to bone tissue adjacent to the defect and used as an indicator of the quality of regenerated bone in reference to healthy tissue. Unstandardised random effect analysis of the BMD (mg/cm^3^) group alone shows no significant effects on bone regeneration by the scaffold + dental pulp stem cells (MD: 0.149, 95% CI − 0.543–0.841, *p* = 0.672). The analysis also shows very high heterogeneity; that is, the effects wildly vary between studies (Fig. 5).

The percentage of newly formed bone was calculated using the following equation:

$$\% {\text{ New bone}}\, = \,\left( {{\text{Area of regenerated bone}}/{\text{Area of created defect}}} \right)\, \times \,{1}00$$

**Fig. 6:**
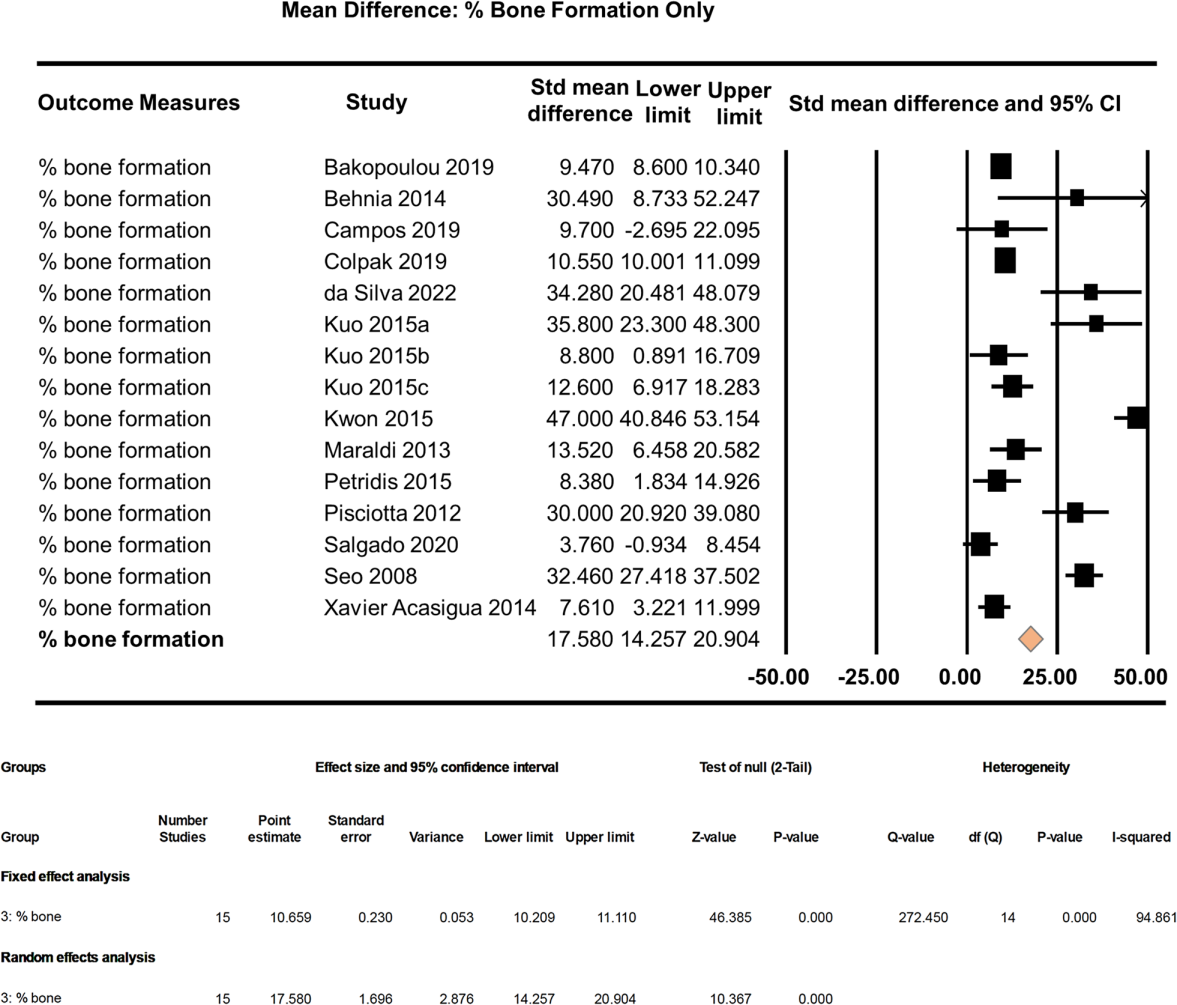
Subgroup effect analysis of % bone formation group

Unstandardised random effect analysis of the % bone formation group alone shows highly significant effects in bone regeneration by the scaffold + dental pulp stem cells (MD: 17.580, 95% CI 14.257–20.904, *p* < 0.0001) compared to the control. On average, the experimental group (scaffold + dental pulp stem cells) scores were almost 18 points higher than the control group scores (Fig. 6).

**Fig. 7:**
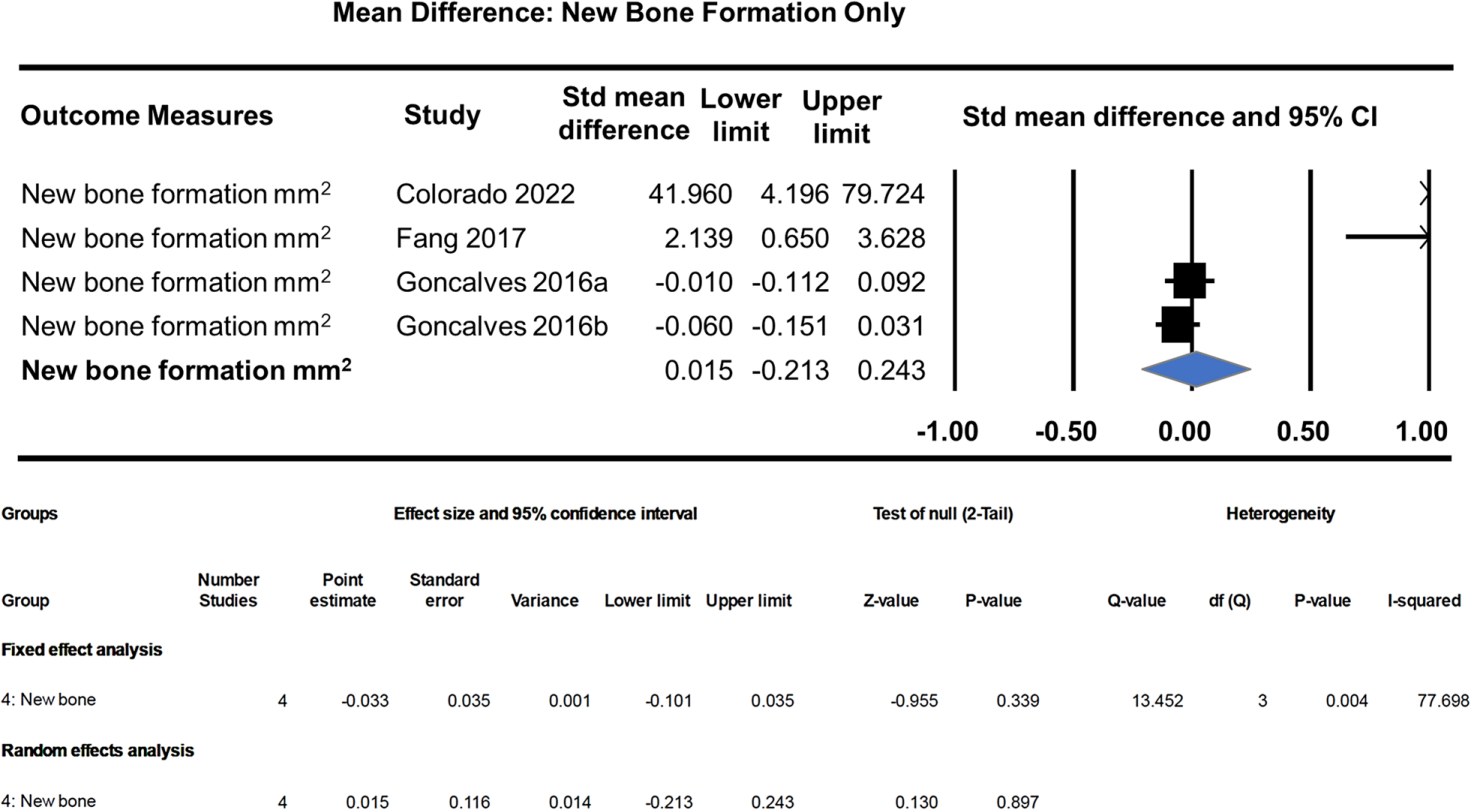
Subgroup effect analysis of new bone formation (mm^2^) group

Unstandardised random effect analysis of the new bone formation mm^2^ group alone shows no significant effects in terms of bone regeneration by the scaffold + dental pulp stem cells (MD: 0.015, 95% CI − 0.213–0.243, *p* = 0.897). The analysis also shows very high heterogeneity, that is, the effects wildly vary between studies (Fig. 7).

### Effect of scaffold on bone regeneration in response to human DPSC/SHED

**Fig. 8:**
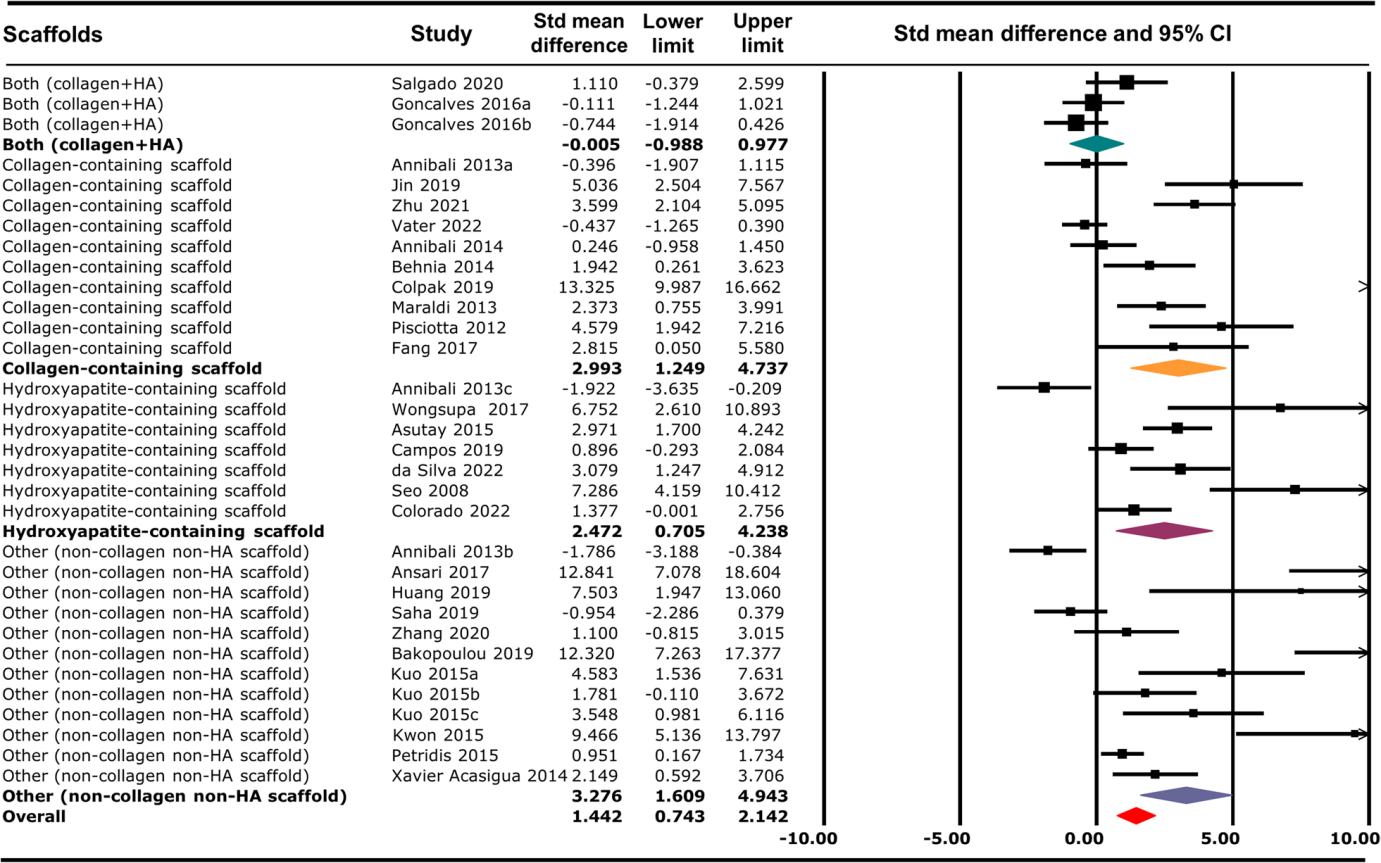
Overall effect of scaffold types on bone regeneration in animal models in response to human DPSC/SHED

Different types of scaffolds were used by the included studies in this meta-analysis. To analyse the effect of scaffold types in bone regeneration, we grouped all the scaffold into 4 groups: 1. collagen-containing scaffold group, 2. hydroxyapatite (HA)-containing scaffold group, 3. both (collagen- and HA-containing scaffold) and 4. other (non-collagen- and non-HA-containing scaffold). Overall, different scaffold groups have significant differences on bone regeneration in combination with DPSC/SHED irrespective of the outcome measure used (MD: 1.442, 95% CI 0.743–2.142, *p* < 0.001). Collagen-containing scaffold regenerate new bone 3 times higher (MD: 2.992, 95% CI 1.249–4.736, *p* < 0.001), HA-containing scaffold regenerate new bone almost 2.5 times higher (MD: 2.471, 95% CI 0.705–4.238, *p* < 0.001), and ‘other group’ regenerate new bone 3 times higher (MD: 3.275, 95% CI 1.608–4.943, *p* < 0.001) in combination with human DPSC/SHED compared to all the other groups (Fig. 8 and Additional file 4).

Unstandardised random effect analysis of the % BV/TV outcome measure among different scaffold groups has shown that only collagen-containing scaffolds regenerate bone almost 10 times higher (MD: 9.740, 95% CI 2.368–17.111, *p* < 0.001) in combination with human DPSC/SHED compared to all the other groups (Additional file 5 and Additional file 6). Analysis of the BMD group has shown that scaffold types does not have any significant effect on bone regeneration (Additional file 7and Additional file 8). Analysis of the % new bone formation group has shown that collagen-containing scaffolds regenerate new bone 18 times higher (MD: 18.80, 95% CI 9.310–28.40, *p* < 0.001), HA-containing scaffolds regenerate new bone almost 26 times higher (MD: 25.872, 95% CI 11.650–40.095, *p* *<* 0.001) and the ‘other’ scaffold group regenerate new bone 18 times higher (MD: 18.004, 95% CI 8.959–27.049, *p* < 0.001), in combination with human DPSC/SHED compared to all the other groups (Additional file 9and Additional file 10). This suggests HA-containing scaffolds have larger effect on bone regeneration compared to other type of scaffolds. Analysis of the new bone formation (mm^2^) group has shown the significant effect of the collagen-containing and HA-containing scaffold on bone regeneration. However, as only 1 study is included in each group true effect cannot be validated for the outcome measure—new bone formation (mm^2^) (Additional file 11and Additional file 12).

### Bone regeneration capacity of different animal species in response to human DPSC/SHED

**Fig. 9:**
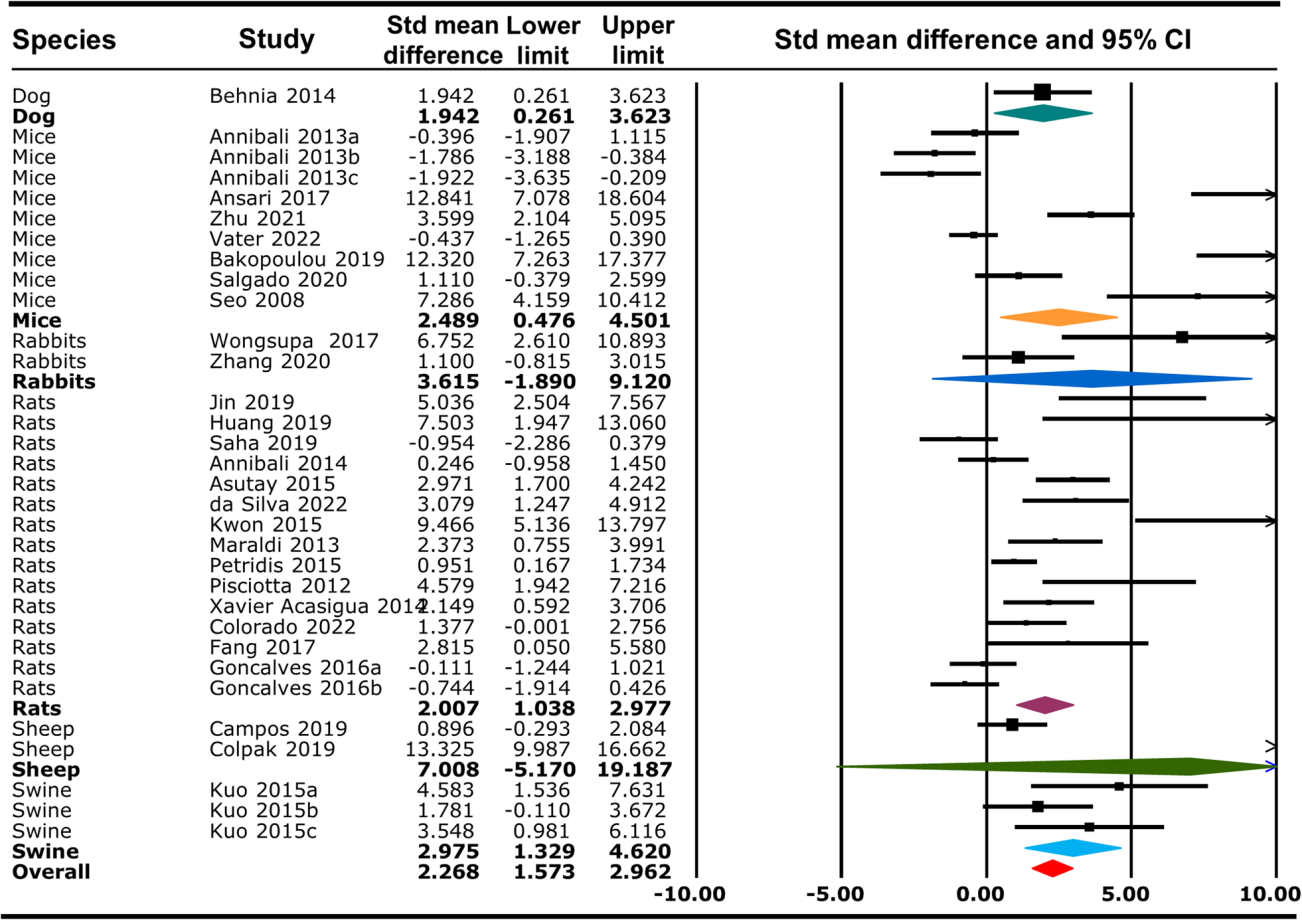
Overall effect of different animal species on bone regeneration in response to human DPSC/SHED

There is a significant difference on bone regeneration among species in response to human DPSC/SHED irrespective of the outcome measure used (MD: 2.268, 95% CI 1.573–2.962, *p* < 0.001). In particular, rats regenerate new bone 2 times higher (MD: 2.007, 95% CI 1.038–2.977, *p* < 0.001), swine regenerate new bone almost 3 times higher (MD: 2.975, 95% CI − 1.329–4.620, *p* < 0.001), mice regenerate new bone 2.5 times higher (MD: 2.489, 95% CI 0.476–4.501, *p* < 0.05) and dog regenerate new bone almost 2 times higher (MD: 1.942, 95% CI 0.261–3.623, *p* < 0.05) in response to human DPSC/SHED compared to other species (Fig. 9 and Additional file 13).

Unstandardised random effect analysis of the % BV/TV outcome measure among different species has shown that rats ((MD: 16.550, 95% CI 12.476–20.624, *p* < 0.001) and rabbits (MD: 12.050, 95% CI 9.194–14.906, *p* < 0.001) regenerate bone significantly higher in response to DPSC/SHED (16 times and 12 times, respectively) compared to other species (Additional file 14and Additional file 15). Animal species have non-significant effect in BMD (Additional file 16 and Additional file 17). Analysis of the % new bone formation group has shown that swine regenerate new bone almost 18 times higher (MD: 17.912, 95% CI 5.450–30.375, *p* < 0.001), sheep regenerate new bone 10 times higher (MD: 10.548, 95% CI 10.00–11.096, *p* < 0.001), dog regenerate new bone 30 times higher (MD: 30.490, 95% CI 8.733–52.247, *p* < 0.001), rats regenerate new bone 23 times higher (MD: 23.200, 95% CI 8.923–37.477, *p* < 0.01) and mice regenerate new bone 15 times higher (MD: 15.115, 95% CI 1.882–28.348, *p* < 0.001) in response to human DPSC/SHED compared to other species (Additional file 18and Additional file 19). This suggests that dog have the highest bone regenerating capacity among all animal species analysed. Analysis of the new bone formation (mm^2^) group has shown that animal species (rats only) does not have any significant effect in bone formation (Additional file 20and Additional file 21).

### Effect of the site of defect on bone regeneration in response to human DPSC/SHED

**Fig. 10:**
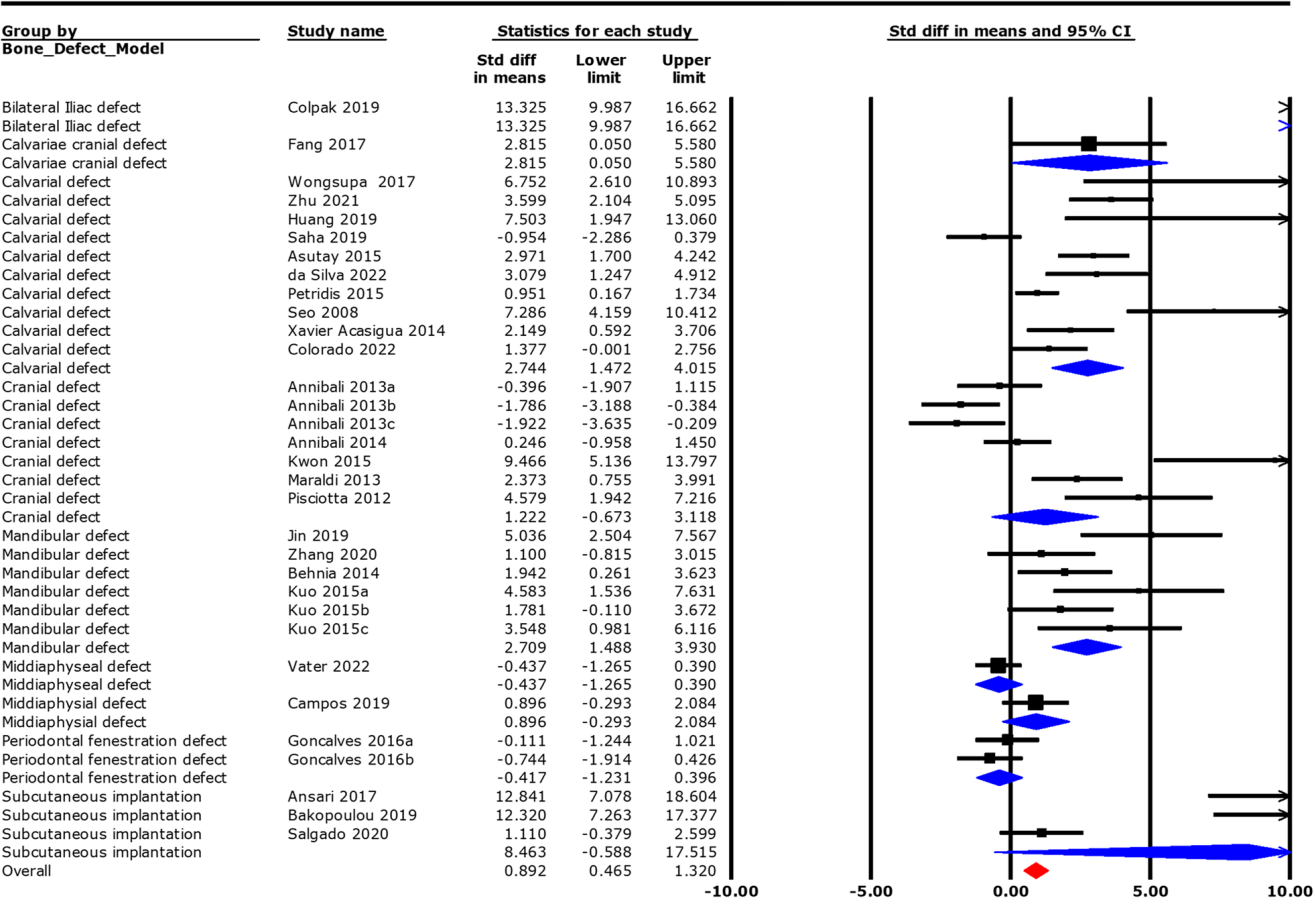
Overall effect of the site of defect in animal on bone regeneration in response to human DPSC/SHED

Overall, different bone defect models have significant differences on bone regeneration (MD: 0.892, 95% CI 0.465–1.319, *p* < 0.001) in response to human DPSC/SHED. However, only calvarial defect (MD: 2.743, 95% CI 1.472–4.017, *p* < 0.001) and mandibular defect (MD: 2.709, 95% CI 1.488–3.930, *p* < 0.001) have shown significantly higher bone regeneration in response to human DPSC/SHED compared to other sites of defects, irrespective of the outcome measure used (Fig. 10 and Additional file 22).

Unstandardised random effect analysis of the %bone formation group only has shown the significant differences in bone regeneration among different bone defect models (MD: 10.562, 95% CI 10.562–11.106, *p* < 0.001).% bone formation in mandibular defect is almost 20 times higher (MD: 19.825, 95% CI 8.380–31.30, *p* < 0.001), in calvarial defect is 20 times higher (MD: 20.127, 95% CI 5.465–34.790, *p* < 0.01) and in cranial defect is 30 times higher (MD: 30.020, 95% CI 9.214–51.20, *p* < 0.001), in response to human DPSC/SHED compared to other defects (Additional file 23 and Additional file 24).

Out of 27 included papers, 25 experimental groups used DPSC, and 7 experimental groups used SHED with the scaffold for bone regeneration (Additional file 25, Additional file 26, Additional file 27, Additional file 28, Additional file 29and Additional file 30). DPSC group had an overall effect of 2.512 (95% CI 1.534–3.490) and SHED had an overall average effect of 2.774 (95% CI 0.815–4.734), meaning no difference on the effect between DPSC and SHED (Additional file 31).

### Sensitivity analysis

**Fig. 11:**
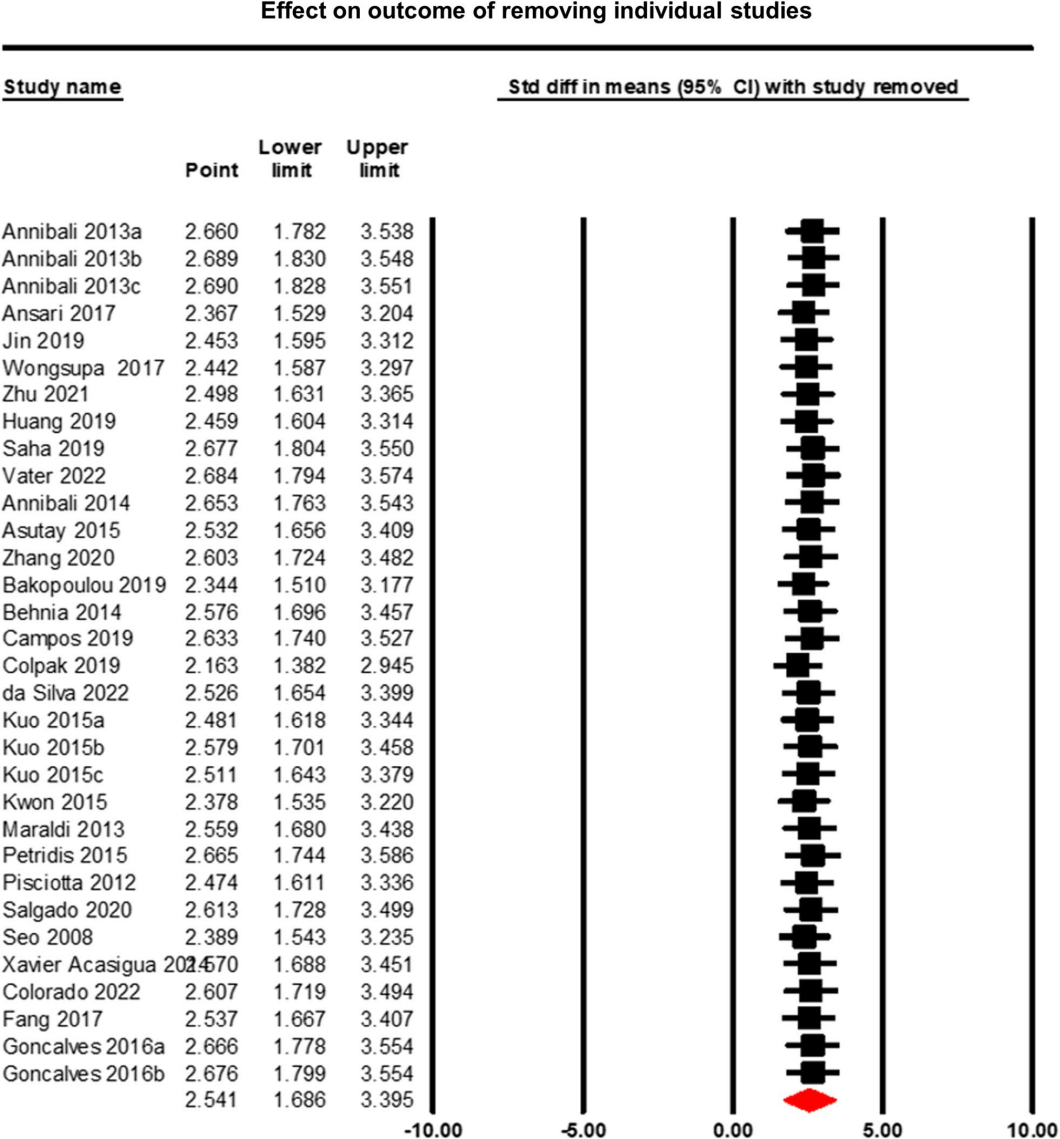
Sensitivity analysis—leave-one-out method. This figure looks at whether any individual studies were unduly influential by rerunning the analysis with that single study removed. This time, each row represents the overall effect that was found when the named study is not included. The overall effect is barely altered by the removal of any one study

**Fig. 12:**
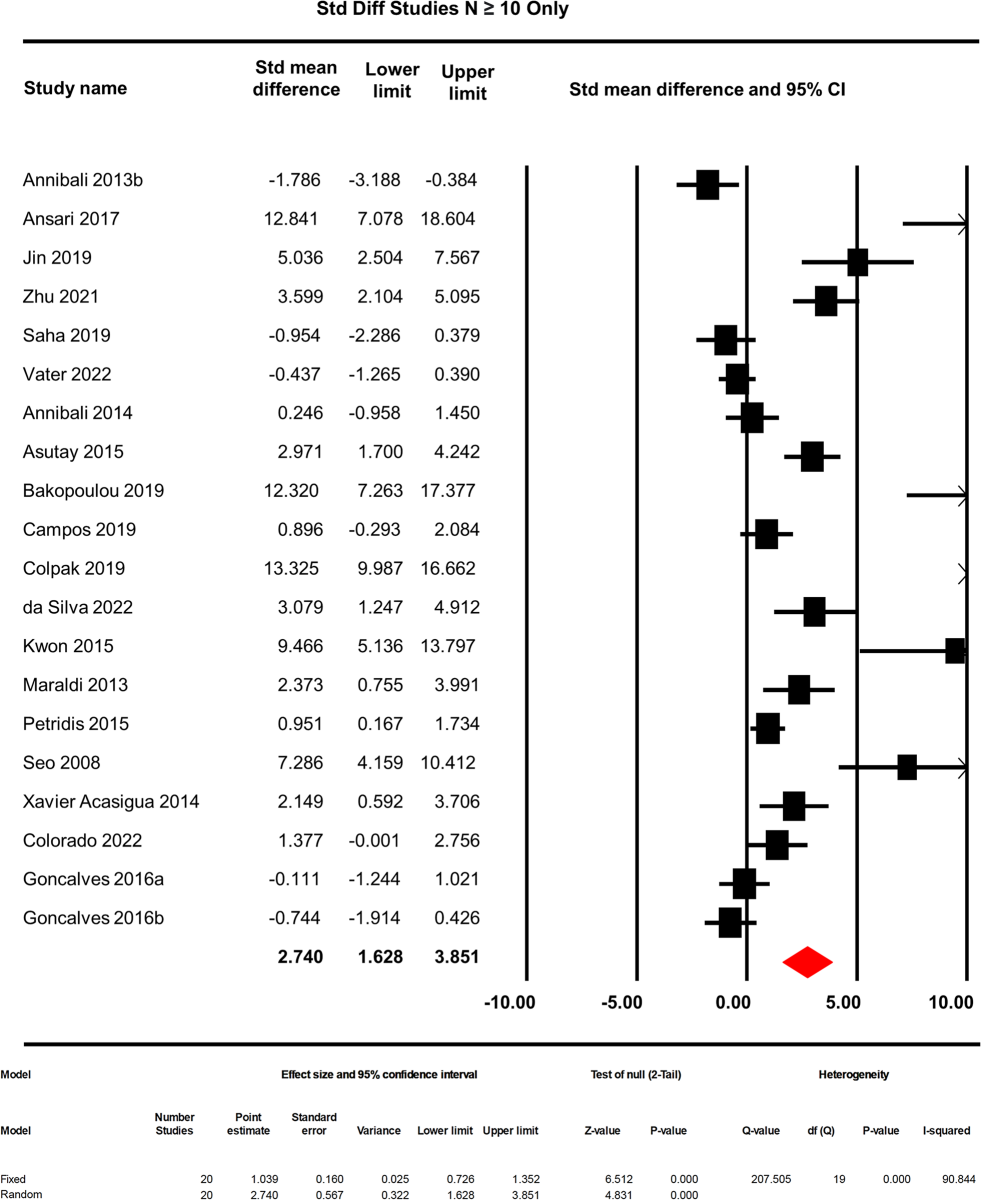
Sensitivity analysis-removing small studies. Studies with sample sizes equal to or more than 10 were included to analyse the changes in effect

Using the leave-one-out method, no single study was identified as a remarkably influential study and removing any single study did not alter neither outcomes nor heterogeneity remarkably (Fig. 11). Excluding the low-quality study (Ansari 2017), the results did not alter significantly either (data not shown). When the effect is calculated using only studies with 10 or more samples, the effect size increases somewhat from 1.8 to 2.7. Only looking at the larger studies has increased the observed effect significantly to a standardised mean difference of 2.740 compared with the original of 1.863 (*p* < 0.0001), meaning the effect of the experimental (scaffold + dental pulp stem cells) group in bone regeneration is greater than control (scaffold) groups (Fig. 12). Therefore, these sensitivity analyses indicate that the results generated in this meta-analysis are robust and reliable.

#### Publication bias

**Fig. 13:**
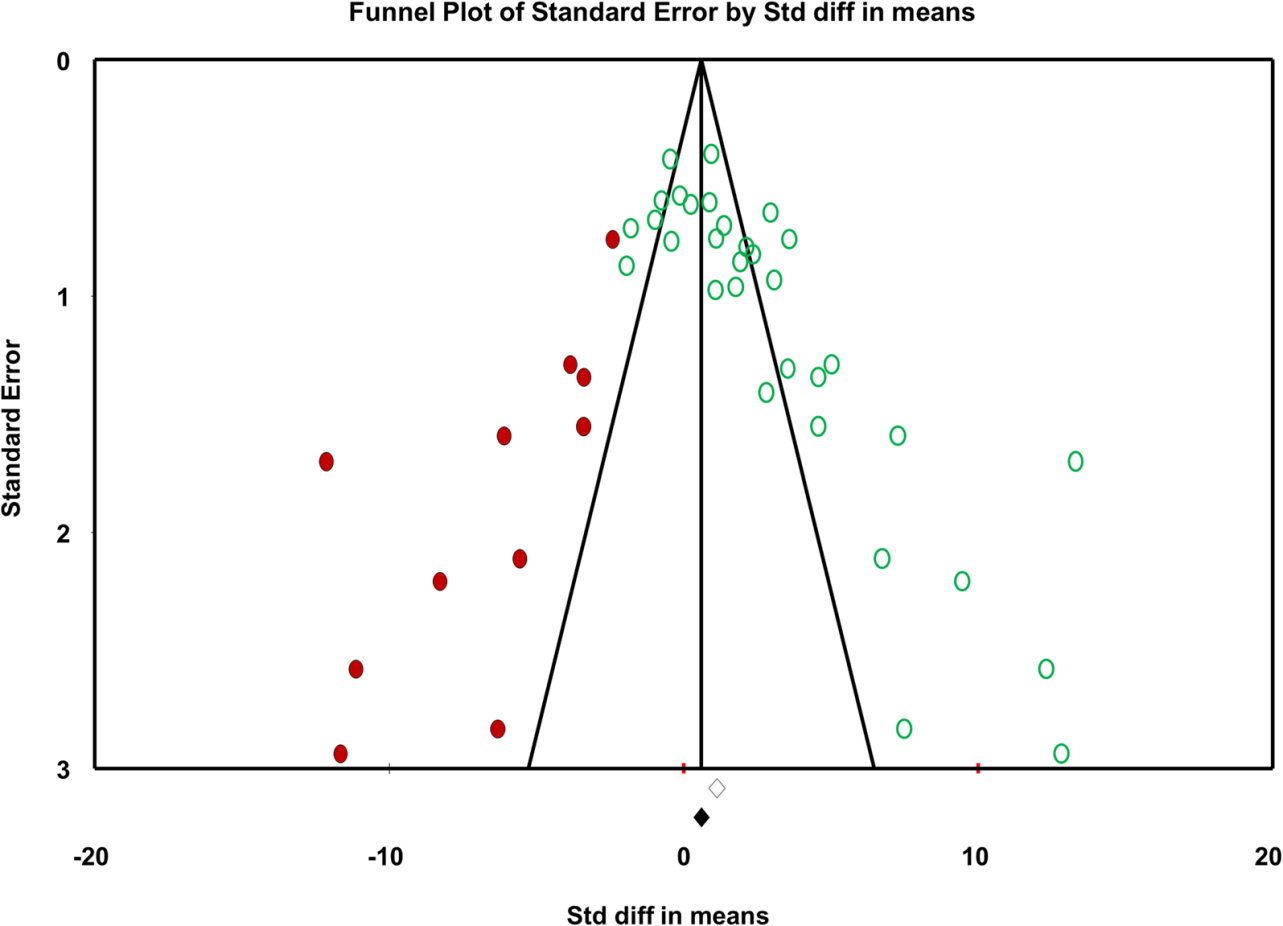
Funnel plot. The observed data are represented by hollow circles and diamonds. The large studies are at the top of the figure and are indeed broadly evenly distributed on either side of the observed effect. Small studies are at the bottom of the figure and do seem to be biased towards very large effect sizes, suggesting possible bias. The filled-in circles and diamonds represent replacing the hypothetical ‘missing’ studies to see how the effect changes. The effect size drops but does not disappear, suggesting publication bias exaggerates effect size though does not explain the effect entirely

The funnel plot is to explore the possibility of publication bias affecting the results. Overall, the plot exhibits no obvious asymmetry representing a lack of remarkable publication bias (Fig. 13).

## Discussion

All the scaffolds were biocompatible and were tested in vitro along with DPSCs or SHED prior to their use in the animal bone defect model. Irrespective of the types or composition of the scaffolds and different outcome measures used for bone regeneration, DPSCs/SHED-incorporated scaffolds enhanced the amount of bone regeneration highly significantly compared to the cell-free scaffold (*p* < 0.0001). In the subgroup analysis, we grouped all the included studies according to the outcome measure units, animal species, scaffold groups, site of defects and two types of dental pulp stem cells: adult DPSCs and SHED. In the outcome measure subgroup analysis, dental pulp stem cells incorporated with the scaffolds in the % bone formation group significantly increased, and in the % BV/TV, BMD-mg/cm^3^, new bone formation-mm^2^ group, it shows no significant difference in bone regeneration in comparison with the scaffold-only control. Dogs and hydroxyapatite-containing scaffolds have the highest % new bone forming capacity in response to human DPSC/SHED. The non-significant effect of the scaffold with dental pulp stem cells in bone regeneration in the % BV/TV, BMD and new bone formation-mm^2^ group can partly be explained by the higher mean difference between the studies and low number of sample sizes in each group. The variable mean differences in our analyses, in case of %BV/TV, BV (mm3), BMD (mg/cm3), % bone formation and new bone formation (mm2), may represent the differences in terms of study design and treatment protocol (i.e. bone defect models, scaffold types and animal species used). In addition, a considerable heterogeneity was observed which also may represent the variable mean differences across the included studies. Besides, studies with smaller sample sizes or weaker study designs may have contributed to the smaller treatment effects than studies with larger sample sizes or stronger designs. These factors altogether may have affected the overall mean differences between the intervention and control groups across the included studies. % BV/TV have only 70 samples (7 studies), bone mineral density (mg/cm^3^) have 74 samples (6 studies), and new bone formation (mm^2^) have only 38 samples (4 studies). In total, 181 samples were tested in the % bone formation group (15 studies) in the meta-analysis. Using a smaller sample size than the ideal undermines the internal and external validity of the results. Thus, sample size calculation is essential in designing a study for methodological and ethical reasons. In this meta-analysis, only 2 papers reported the sample size calculations. The importance of sample sizes was further confirmed when studies with less than 10 samples were excluded in the sensitivity analysis and showed a highly significant (*p* < 0.0001) increase in the effect of the scaffold + stem cell group in bone regeneration from 1.863 to 2.740.

Dental stem cells were first isolated and characterised from the dental pulp of the adult permanent teeth (DPSC) and subsequently from the dental pulp of deciduous teeth (milk teeth) (SHED) [68]. SHED has been reported to exhibit a higher proliferation rate, differentiation potential and increased mineralisation capacity in vivo compared to DPSCs due to their origin from a more immature subpopulation than permanent teeth [69]. However, Nakajima et al*.* (2018) reported that SHED and human DPSC transplantation in the mice bone defect model exhibited nearly the same quantity of new bone formation [11]. In this meta-analysis, the scaffold + DPSC group and the scaffold + SHED group also show no difference in effect on bone regeneration. This observation is the first evidence in synthesising the data published on the role of DPSC and SHED in bone regeneration in animal models. This meta-analysis shows the evidence that the DPSCs and SHED play vital roles in bone regeneration irrespective of the type of scaffold used. The purpose of using human DPSC/SHED in the animal bone defect model is to explore their bone regeneration ability. In this meta-analysis, it is evident that human DPSC/SHED have successfully differentiated into bone forming cells and regenerate bone in the animal defect areas more than the cell-free group. This indicates that the formed bone tissues are mostly donor-derived. Some studies also analysed the fate of the transplanted stem cells using human mitochondrial antibody and have shown the presence of human cells in the regenerated bone in animals [42, 52, 59]. However, all the studies have shown some bone formation in the scaffold-only (cell-free) group meaning host-derived bone formation occurred in some extent in response to the scaffolds.

Impacted and unerupted wisdom teeth (3rd molar) extraction is one of the most routine procedures in oral surgery, and the extracted teeth can be recycled for dental pulp stem cell extraction [70, 71]. SHED, the immature MSCs are obtained from naturally exfoliated deciduous teeth. Thus, teeth could offer unique, easily accessible and non-invasive (particularly for deciduous teeth) stem cell resources with limited legal and ethical concerns [72, 73]. Removal of autogenous grafts from other sites have associated with morbidity, and the use of SHED or DPSC may avoid those inconveniences. Moreover, contrary to autologous bone grafts, SHED or DPSC can be multiplied in vitro prior to their use in vivo to generate the suitable number of cells for the tissue being restored. Applying this principle could decrease or prevent issues associated with the autogenous grafting method, such as the risk of infection and the limited amount of tissue that can be extracted from the donor site [74]. Furthermore, DPSC and SHED are originated from the neural crest, which makes them mostly compatible with the regeneration and repair of neural crest-derived tissues, e.g. jawbone [75, 76]. DPSCs have already attracted interest as an alternative to improve the outcome of dental implants [77]. Studies such as Alge et al*.* [78] and Stanko et al*.* [79] also indicated that DPSCs are more proliferative, have a higher percentage of stem cells and possess higher osteogenic potential than bone marrow stem cells (BMSCs), which are still regarded as the gold standard for bone tissue formation [77].

So far, researchers are investigating various approaches to utilise stem cells for bone regeneration; but, in order to exploit the full potential of stem cell therapy, the scaffold should hold the stem cells at the implantation site and maintain the essential characteristics of stem cells such as self-renewal and stimulate them to differentiate [80]. From this systematic review, it is observed that out of 49 articles, only 4 articles provided evidence that the scaffold + dental stem cell group did not enhance the new bone formation compared to the scaffold-only group. Annibali et al*.* [20] used three different types of scaffolds, namely GDPB (Bio-Oss) + Collagen, β-TCP and Agarose + nanohydroxyapatite with DPSCs, and all the scaffolds enhanced the new bone formation alone. Gonҫalves et al*.* [34] revealed that polyester poly (isosorbide succinate-co-L-lactide) (PisPLLA) + Collagen + hydroxyapatite and poly (L-lactide) (PLLA) + collagen + hydroxyapatite alone could form new bone more than the scaffold + SHED combination in rats. Jahanbin et al*.* [38] used a collagen scaffold in combination with DPSC, but collagen alone formed new bone more than the combination with cells. Vater et al*.* [61] incorporated DPSC and BMSC into mineralised collagen matrix in a rat bone defect model, but pre-seeding with either of the cells did not enhance bone defect healing. The author argued that the inability of the dental pulp stems to enhance the new bone formation can be explained by various factors such as: 1. the nature of the scaffolds interfered with the stem cells osteogenic differentiation in the microenvironment of the defect or 2. difficulty in positioning the graft in the experiment wound or 3. the created microenvironment was not optimal to generate sufficient osteogenic activity or 4. the lack of appropriate differentiation factors and most importantly, 5. the presence of pro-inflammatory mediators could regress the osteogenic trend of the stem cells [20, 34, 38, 61]. There are still challenges in designing an ideal scaffold which not only should support the complex structure of bone defects to guide bone tissue regeneration, but also, provide a porous microenvironment to employ biological factors and stimulate dental pulp stem cell growth and differentiation. However, with the evidence synthesised in this meta-analysis, it is clear that the advent of bone tissue engineering with the incorporation of osteogenic capable dental pulp stem cells has certainly increased scaffold effectiveness, increased new bone formation and added further versatility in bone defect therapy. So far, only three human clinical trials on bone regeneration reported results with pre-seeded dental pulp stem cells with the scaffold and showed evidence of a positive outcome. D’aquino et al*.* (2009) reported a split-mouth-controlled trial on 17 patients with socket preservation using DPSCs pre-seeded on collagen sponge for 1 year. They revealed that optimal vertical repair and complete restoration of periodontal tissue in the mandible bone defect were higher at the test site the control site [81]. Hernández-Monjaraz B et al*.* (2014) reported preliminary findings of a case study on a patient with periodontal disease. They pre-seeded SHED with collagen + polyvinylpyrrolidone sponge and implanted it in the pre-molar area. After 6 months, the patient exhibited a reduction in tooth mobility, periodontal pocket depth and bone defect area and an increase in bone mineral density [82]. Tanikawa D et al*.* (2020) also reported the result of a case series on 6 cleft lip and palate patients. They also pre-seeded SHED with hydroxyapatite–collagen sponge and grafted in the maxillary alveolar defect. SHED therapy resulted in satisfactory bone healing in this case series [83].

We, however, acknowledge some limitations in this systematic review and meta-analysis. Although most of the included studies (90%) were either medium or low-risk, only 6% reported the most important method to avoid bias—the blinded implantation or insertion of the experimental and control groups. This may increase the substantial risk of misunderstanding the effect of scaffold + dental pulp stem cells on bone regeneration. Included articles differed in animal species, sex, bone defect model and the healing time. Furthermore, we found that the studies dealt with the regeneration of different bones by utilising different bone defect models with different degrees of complexities, and there was lack of homogenisation between studies in terms of the analysis of new bone formation; therefore, the result obtained cannot be standardised. Due to the heterogeneity of the results, we were only able to analyse 27 articles out of 49, grouped by 4 different units of bone regeneration analysis. However, we tried to reduce bias in the systematic review by independent screening, data extraction, evaluation of results and risk of bias evaluation by at least two blind evaluators.

## Conclusion

Since the discovery of dental pulp stem cells, this is the first meta-analysis that synthesised the evidence of the effect of dental pulp stem cells pre-seeded with the scaffold on bone regeneration in animal models. This study also revealed strong evidence of an increase in new bone formation in response to the ‘dental pulp stem cells and scaffold’ combination therapy. The increase in the ageing population and traumatic injury creates a massive socioeconomic and healthcare burden, resulting in a prime need for bone tissue [84, 85]. As the current gold standard therapies for healing bone defects, autografts suffer from restricted supply and injury at the donor site; however, the tissue engineering approach of incorporating dental pulp stem cells with the biocompatible scaffold could meet the rising demand for clinically relevant bone tissue. The clinical trials and clinical applications of dental pulp stem cells on bone regeneration are still in their infancy due to the large gap in basic and translational research. Synthesised evidence from this meta-analysis and a few published clinical trials indicate that dental pulp stem cells would be a promising tool for treating various bone diseases, and more clinical trials should be conducted to evaluate the effectiveness of the dental pulp stem cell-based therapy.

## Supplementary Information

**Additional file 1.** Search Strategy. Embase database as an example.

**Additional file 2.** Inflammatory reaction in response to human DPSC/SHED in animals.

**Additional file 3.** Reasons for exclusion from meta-analysis.

**Additional file 4.** Overall effect of scaffold type on bone regeneration.

**Additional file 5.** Raw mean difference of the effect of scaffold type on % BV/TV.

**Additional file 6.** Detailed mean difference and the significance of the effect of scaffold type on % BV/TV.

**Additional file 7.** Raw mean difference of the effect of scaffold type on BMD.

**Additional file 8.** Detailed mean difference and the significance of the effect of scaffold type on BMD.

**Additional file 9.** Raw mean difference of the effect of scaffold type on % new bone formation.

**Additional file 10.** Detailed mean difference and the significane of the effect of scaffold type on % new bone formation.

**Additional file 11.** Raw mean difference of the effect of scaffold type on new bone formation (mm2).

**Additional file 12.** Detailed mean difference and the significance of the effect of scaffold type on new bone formation (mm2).

**Additional file 13.** Overal effect of animal species on bone regeneration.

**Additional file 14.** Raw mean difference of the effect of animal species on % BV/TV.

**Additional file 15.** Detailed mean difference and the significance of the effect of species on % BV/TV.

**Additional file 16.** Raw mean difference of the effect of animal species on BMD.

**Additional file 17.** Detailed mean difference and the significance of the effect of animal species on BMD.

**Additional file 18.** Raw mean difference of the effect of animal species on % new bone formation.

**Additional file 19.** Detailed mean difference and the significance of the effect of animal species on % new bone formation.

**Additional file 20.** Raw mean difference of the effect of animal species on new bone formation (mm2).

**Additional file 21.** Detailed mean difference and the significane of the effect of species on new bone formation (mm2).

**Additional file 22.** Overall effect of the site of defect in animals on bone regeneration.

**Additional file 23.** Raw mean difference of the effect of defect sites on % BV/TV.

**Additional file 24.** Detailed mean difference and the significance of the defect sites on % BV/TV.

**Additional file 25.** Raw mean difference of the effect of defect sites on BMD.

**Additional file 26.** Detailed mean difference and the significance of the defect sites on BMD.

**Additional file 27.** Raw mean difference of the effect of defect sites on % new bone formation.

**Additional file 28.** Detailed mean difference and the significance of the defect sites on % new bone formation.

**Additional file 29.** Raw mean difference of the effect of defect sites on new bone formation (mm2).

**Additional file 30.** Detailed mean difference and the significance of the defect sites on new bone formation (mm2).

**Additional file 31.** Overall effect of the type of dental pulp stem cells on bone regeneration.

## Data Availability

The datasets used and/or analysed during the current study are available from the corresponding author upon reasonable request.

## Abbreviations

hDPSC: Human dental pulp stem cell
SHED: Stem cells from human exfoliated deciduous teeth
%BV/TV: Per cent bone volume/total volume
BMD: Bone mineral density
