# Supplementary material for: Evaluating the efficacy of human dental pulp stem cells and scaffold combination for bone regeneration in animal models: a systematic review and meta-analysis
Source: Stem Cell Res Ther. 2023 May 15;14:132. doi: 10.1186/s13287-023-03357-w (PMC10186750; doi:10.1186/s13287-023-03357-w)
Supplement: Supplementary file 1 — Additional file 1. Search Strategy. Embase database as an example. [file 13287_2023_3357_MOESM1_ESM.docx]

| Set |  | | Search Statement | # Results |
| --- | --- | --- | --- | --- |
| 1 |  | | Dental pulp mesenchymal stem cells | 113 |
| 2 |  | | DPSC | 1214 |
| 3 |  | | Dental Pulp Stem Cells | 4976 |
| 4 |  | | SHED | 153010 |
| 5 |  | | Stem cells from human exfoliated deciduous teeth | 798 |
| 6 |  | | Bone regeneration | 59482 |
| 7 |  | | Bone regenerations | 38 |
| 8 |  | | Osteo-regeneration | 17 |
| 9 |  | | Osteoregeneration | 86 |
| 10 |  | | Guided bone regeneration | 4492 |
| 11 |  | | 1 or 2 or 3 or 4 or 5 | 158056 |
| 12 |  | | 6 or 7 or 8 or 9 or 10 | 59528 |
| 13 |  | | Scaffold | 180818 |
| 14 |  | | Scaffolds | 127532 |
| 15 |  | | Scaffold Matrix | 618 |
| 16 |  | | Scaffold for bone regeneration | 471 |
| 17 |  | | Scaffolding | 25651 |
| 18 |  | | Scaffoldings | 191 |
| 19 |  | | Bone substitute | 6427 |
| 20 |  | | Bone substitutes | 13941 |
| 21 |  | | Bone Augmentation material | 53 |
| 22 |  | | Alloplastic material | 818 |
| 23 |  | | Bone graft | 81000 |
| 24 |  | | Xenograft | 217091 |
| 25 |  | | Allograft | 176322 |
| 26 |  | | Ceramics | 47177 |
| 27 |  | | Autograft | 40134 |
| 28 |  | | 13 or 14 or 15 or 16 or 17 or 18 or 19 or 20 or 21 or 22 or 23 or 24 or 25 or 26 or 27 | 794,373 |
| 29 |  | | 11 and 12 and 28 | 316 |
|  | |  | | |

Table S1: Search strategy
