# Supplementary material for: Evaluating the efficacy of human dental pulp stem cells and scaffold combination for bone regeneration in animal models: a systematic review and meta-analysis
Source: Stem Cell Res Ther. 2023 May 15;14:132. doi: 10.1186/s13287-023-03357-w (PMC10186750; doi:10.1186/s13287-023-03357-w)
Supplement: Supplementary file 2 — Additional file 2. Inflammatory reaction in response to human DPSC/SHED in animals. [file 13287_2023_3357_MOESM2_ESM.docx]

**Table S2: Inflammatory reaction in response to human DPSC/SHED in animals**

| **References** | **Information on inflammatory reaction** |
| --- | --- |
| Annibali 2014 | Immunodeficient rats, immune reaction was not discussed |
| Annibali 2013 | Immunodeficient mice, immune reaction was not discussed |
| Ansari 2017 | Both wild type and immunodeficient mice. Host T-lymphocytes prevented SHED mediated ostoegenesis in nude mice. Encapsulated SHED into the hydrogel construct prevented T-lymphocytes to infiltrate, thus enhanced the osteogenesis |
| Asutay 2015 | Non-immunosuppressed Albino rats. Graft was not rejected, indicated that DPSC elicit immunosuppressive effect. |
| Bakopoulou 2019 | Immunocompromised mice. Immune reaction was not analysed but hypothesised that the scaffold, Chitosan has inherent immune-stimulatory properties |
| Behnia 2014 | Fiest dog, No immune reaction. SHED has immunomodulatory properties |
| Bressan 2012 | Immunodeficient mice. No immune reaction was observed after implantation |
| Campos 2019 | Non-immunosuppressed sheep. No immune reaction. Discussed that the DPSC elicit immunosuppressive effect. |
| Colorado 2022 | Non-immunocompromised Wistar SPF rats. No immune reaction was observed |
| Colpak 2019 | Non-immunocompromised Sheep. Immune reaction was not discussed |
| da Silva 2022 | Non-immunosuppressed Wistar rats. No immune reaction was observed, and SHED’s immunosuppressive effect has proven. |
| Fahimipour 2019 | Non-immunocompromised Fisher 344 rats. No immune reaction was observed |
| Fang 2017 | Immunodeficient Sprague-Dawley Rats. Immune reaction was not discussed |
| Fu 2018 | Immunodeficient mice. Immune reaction was not discussed |
| Ghavimi 2020 | Non-immunocompromised Mongrel dogs. No significant immune reaction was observed. |
| Gonçalves 2016 | Non-immunosuppressed Wistar rats. Immune reaction was not discussed |
| Gutiérrez-Quintero 2020 | Non-immunosuppressed New Zealand albino rabbits. No immune reaction was observed |
| Hiraki 2020 | Immunodeficient BALB/c-nu Mice. Immune reaction was not discussed |
| Huang 2019 | Immunodeficient Sprague-Dawley Rats. Immune reaction was not discussed |
| Jahanbin 2016 | Non-immunosuppressed Wistar rats. Significant immune reaction was observed |
| Jin 2019 | Rats. Type of rats or immune information not disclosed |
| Kang 2017 | Immunodeficient athymic mice. Inflammation was not observed. |
| Kawanabe 2012 | Immunodeficient Fox-Chase SCID mice. Information on immune reaction was not discussed |
| Kunwong 2021 | Immunodeficient Sprague–Dawley rats. Inflammatory cell infiltration was observed. |
| Kuo 2015 | Non-immunocompromised pigs. Mild inflammatory reaction was observed in first 2 weeks. |
| Kwon 2015 | Immunodeficient Sprague–Dawley rats. Information on immune reaction was not discussed |
| Liu 2015 | Immunodeficient C57BL/6J mice and Beige nude/nude Xid (III) mice. Acetyl Salicylic Acid enhanced the immunomodulatory function of SHED |
| Man 2022 | Immunodeficient CD1 nude mice. Information on immune reaction was not discussed. |
| Maraldi 2013 | Immunodeficient CD® IG5 rats. Information on immune reaction was not discussed. |
| Mohanram 2020 | Immunodeficient MF1 Nu/Nu mice. Information on immune reaction was not discussed. |
| Nakajima 2018 | Immunodeficient BALB/c-nu Mice. Immune reaction was not discussed |
|  |  |
| Niu 2014 | Immunodeficient nude Mice. Immune reaction was not discussed |
| Novais 2019 | Immunodeficient athymic (nude)"NMRI Foxn1 nu/nu" mice. Immune reaction was not discussed |
| Petridis 2015 | Non-immunosuppressed Wistar rats. Inflammatory cells were present |
| Pisciotta 2012 | Immunodeficient Sprague–Dawley rats. Information on immune reaction was not discussed |
| Prabha 2018 | Immunodeficient NOD.CB17-Prkdcscid/J mice. No signs of inflammation |
| Prahasanti 2020 | Non-immunosuppressed Wistar rats. Information on immune reaction was not discussed |
| Prahasanti 2019 | Non-immunosuppressed Wistar rats. Information on immune reaction was not discussed |
| Saha 2019 | Immunodeficient athymic nude (Foxn1-rnu) rats. Information on immune reaction was not discussed. |
| Salgado 2020 | Immunodeficient nude mice. Information on immune reaction was not discussed. |
| Saskianti 2022 | Non-immunosuppressed Wistar rats. SHED played immunomodulation role toward an inflammatory response suppression |
| Saskianti 2018 | Non-immunosuppressed brown rats. Information on immune reaction was not discussed. |
| Seo 2008 | Immunodeficient NIH-bgnu-xid, Harlan Sprague–Dawley mice. Information on immune reaction was not discussed. |
| Serano-Bello 2020 | Non-immunosuppressed Wistar rats. No signs of inflammatory response |
| Vater 2022 | Immunodeficient NMRI nude mice. Author hypothesised that DPSC might be acted as anti-inflammatory that inhibited bone regeneration. |
| Wongsupa 2017 | Non-immunosuppressed New Zealand white rabbits. Minimal sign of inflammation. |
| Xavier Acasigua 2014 | Non-immunosuppressed Wistar rats. No signs of inflammatory response |
| Zhang 2020 | Non-immunosuppressed New Zealand white rabbits. No sign of inflammation or host rejection |
| Zhu 2021 | Immunodeficient nude mice. No signs of inflammatory response |
