# Supplementary material for: Evaluating the efficacy of human dental pulp stem cells and scaffold combination for bone regeneration in animal models: a systematic review and meta-analysis
Source: Stem Cell Res Ther. 2023 May 15;14:132. doi: 10.1186/s13287-023-03357-w (PMC10186750; doi:10.1186/s13287-023-03357-w)
Supplement: Supplementary file 3 — Additional file 3. Reasons for exclusion from meta-analysis. [file 13287_2023_3357_MOESM3_ESM.docx]

Table S3: Reasons for exclusion from meta-analysis

| **References** | **Reasons for exclusion from meta-analysis** |
| --- | --- |
| Bressan 2012 | Lack of quantitative data for osteogenic marker expression |
| Fahimipour 2019 | Lack of quantitative data for osteogenic marker expression |
| Fu 2018 | Lack of quantitative data for osteogenic marker expression |
| Ghavimi 2020 | Lack of quantitative data for new bone formation |
| Gutiérrez-Quintero 2020 | Only study that used new bone formation (mm) as bone regeneration unit |
| Hiraki 2020 | Only study that used bone volume (mm^3^) as bone regeneration unit |
| Jahanbin 2016 | Missing SD/SEM data |
| Kang 2017 | Missing SD/SEM data |
| Kawanabe 2012 | Lack of quantitative data for osteogenic marker expression |
| Kunwong 2021 | Lack of quantitative data for osteogenic marker expression |
| Liu 2015 | Lack of quantitative data for ‘scaffold only negative control’ group |
| Man 2022 | Lack of quantitative data for osteogenic marker expression |
| Mohanram 2020 | Lack of quantitative data for osteogenic marker expression |
| Nakajima 2018 | Lack of quantitative data for ‘scaffold only negative control’ group |
| Niu 2014 | Lack of quantitative data for osteogenic marker expression |
| Novais 2019 | Lack of quantitative data for ‘scaffold only negative control’ group |
| Prabha 2018 | Lack of quantitative data for osteogenic marker expression |
| Prahasanti 2020 | Lack of quantitative data for osteogenic marker expression |
| Prahasanti 2019 | Lack of quantitative data for osteogenic marker expression |
| Saskianti 2022 | Lack of quantitative data for osteogenic marker expression |
| Saskianti 2018 | Lack of quantitative data for osteogenic marker expression |
| Serano-Bello 2020 | Lack of quantitative data for ‘scaffold only negative control’ group |
