# Supplementary figures and images for: Evaluating the efficacy of human dental pulp stem cells and scaffold combination for bone regeneration in animal models: a systematic review and meta-analysis
Source: Stem Cell Res Ther. 2023 May 15;14:132. doi: 10.1186/s13287-023-03357-w (PMC10186750; doi:10.1186/s13287-023-03357-w)

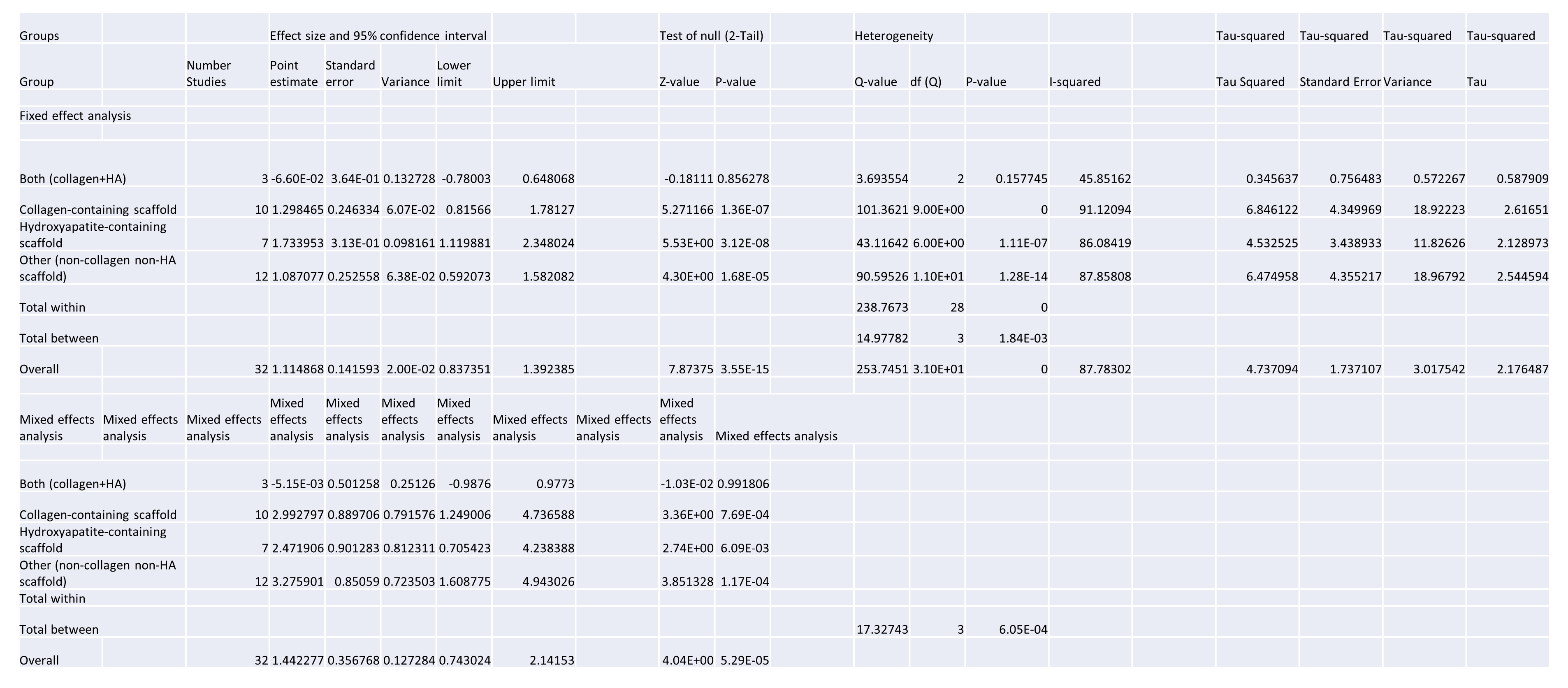

Supplement: Supplementary file 4 — Additional file 4. Overall effect of scaffold type on bone regeneration. [file 13287_2023_3357_MOESM4_ESM.tif]

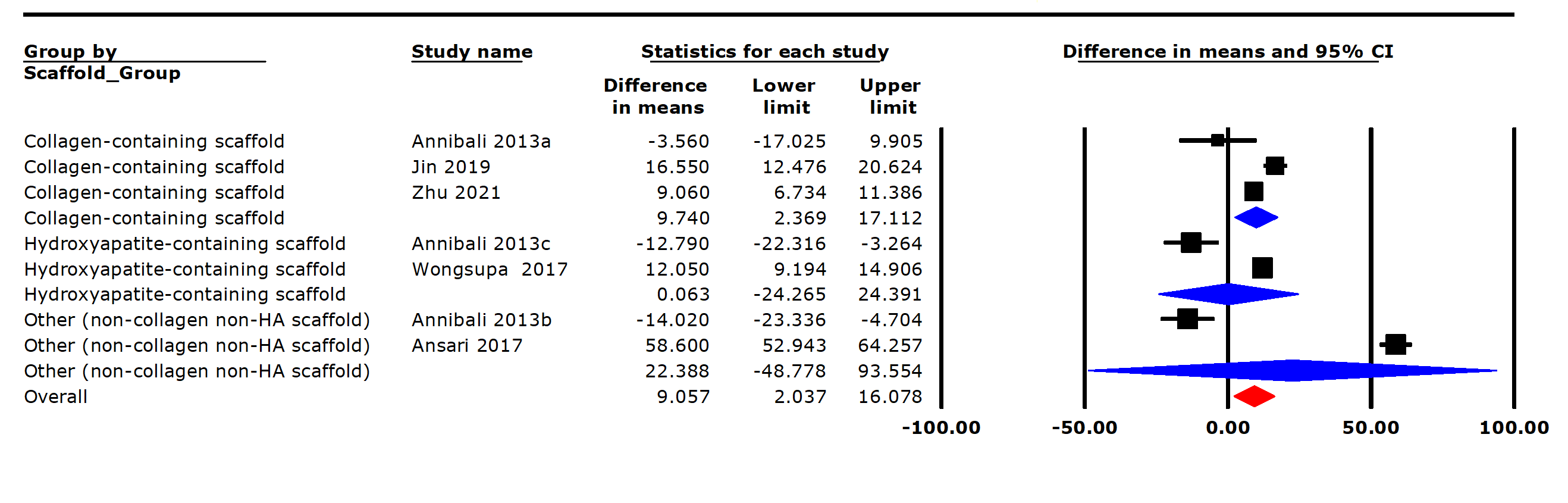

Supplement: Supplementary file 5 — Additional file 5. Raw mean difference of the effect of scaffold type on % BV/TV. [file 13287_2023_3357_MOESM5_ESM.tif]

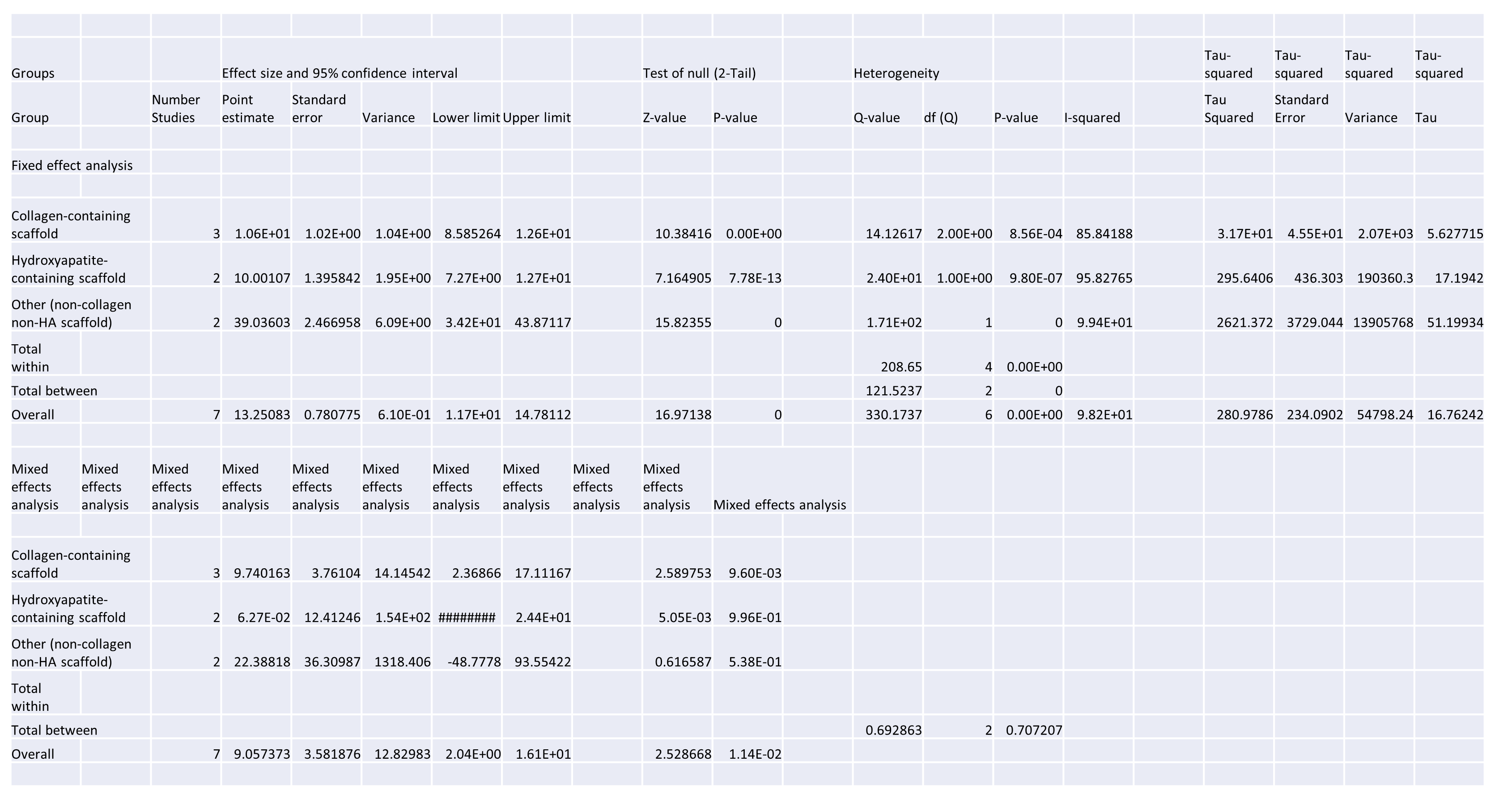

Supplement: Supplementary file 6 — Additional file 6. Detailed mean difference and the significance of the effect of scaffold type on % BV/TV. [file 13287_2023_3357_MOESM6_ESM.tif]

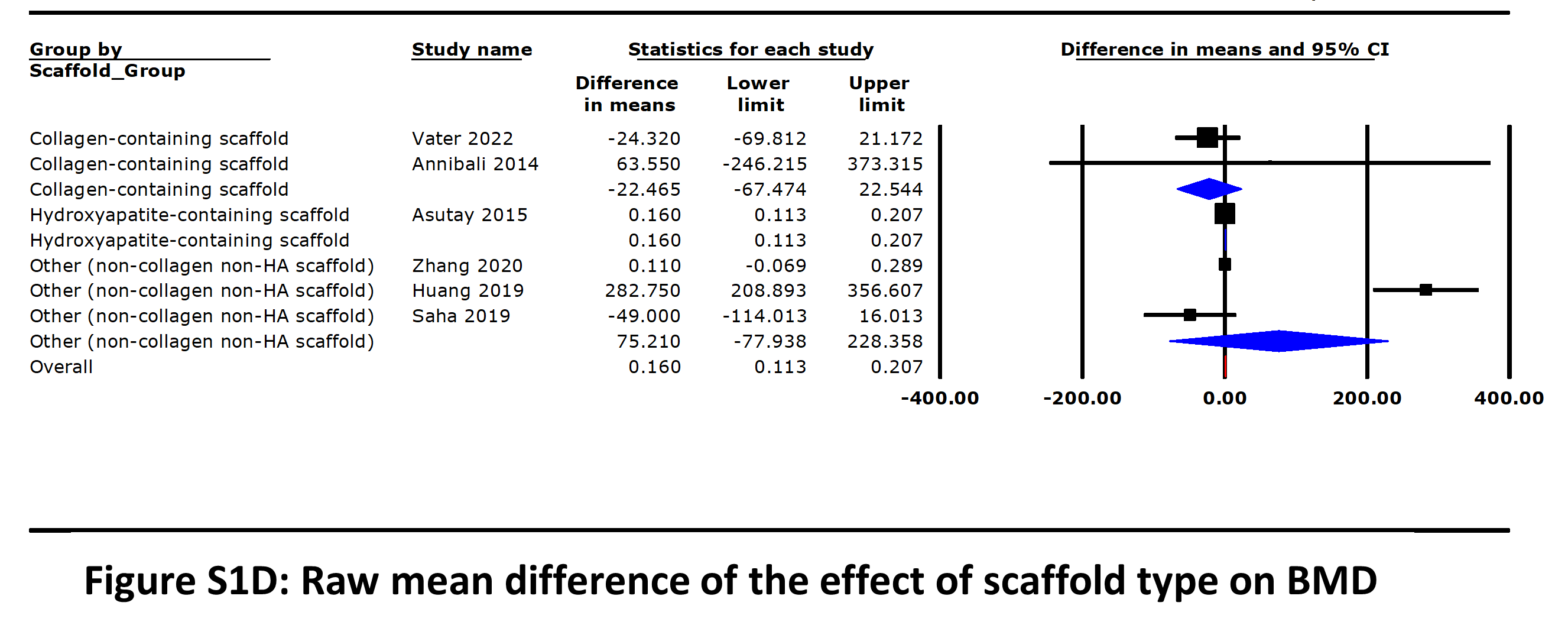

Supplement: Supplementary file 7 — Additional file 7. Raw mean difference of the effect of scaffold type on BMD. [file 13287_2023_3357_MOESM7_ESM.tif]

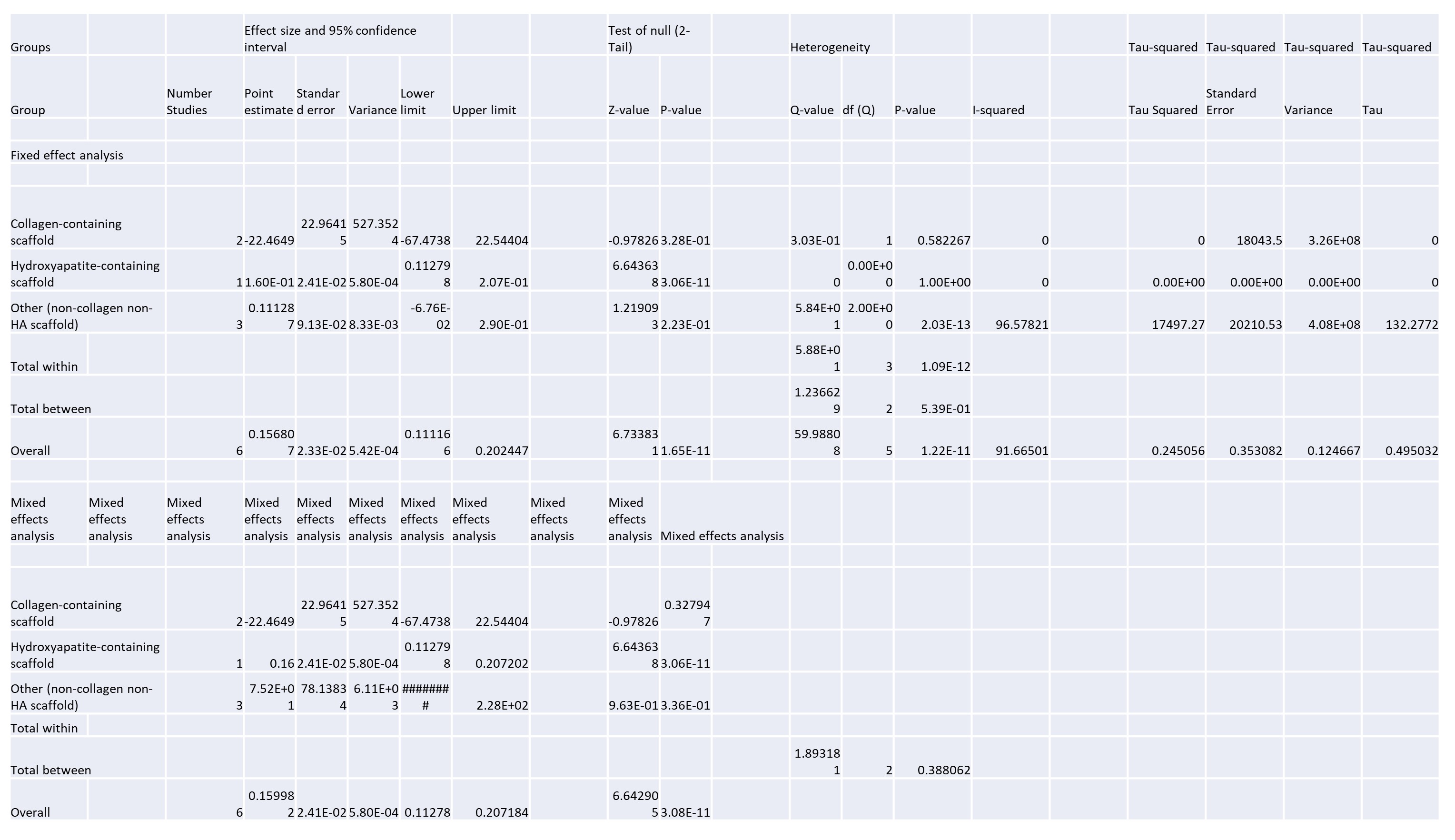

Supplement: Supplementary file 8 — Additional file 8. Detailed mean difference and the significance of the effect of scaffold type on BMD. [file 13287_2023_3357_MOESM8_ESM.tif]

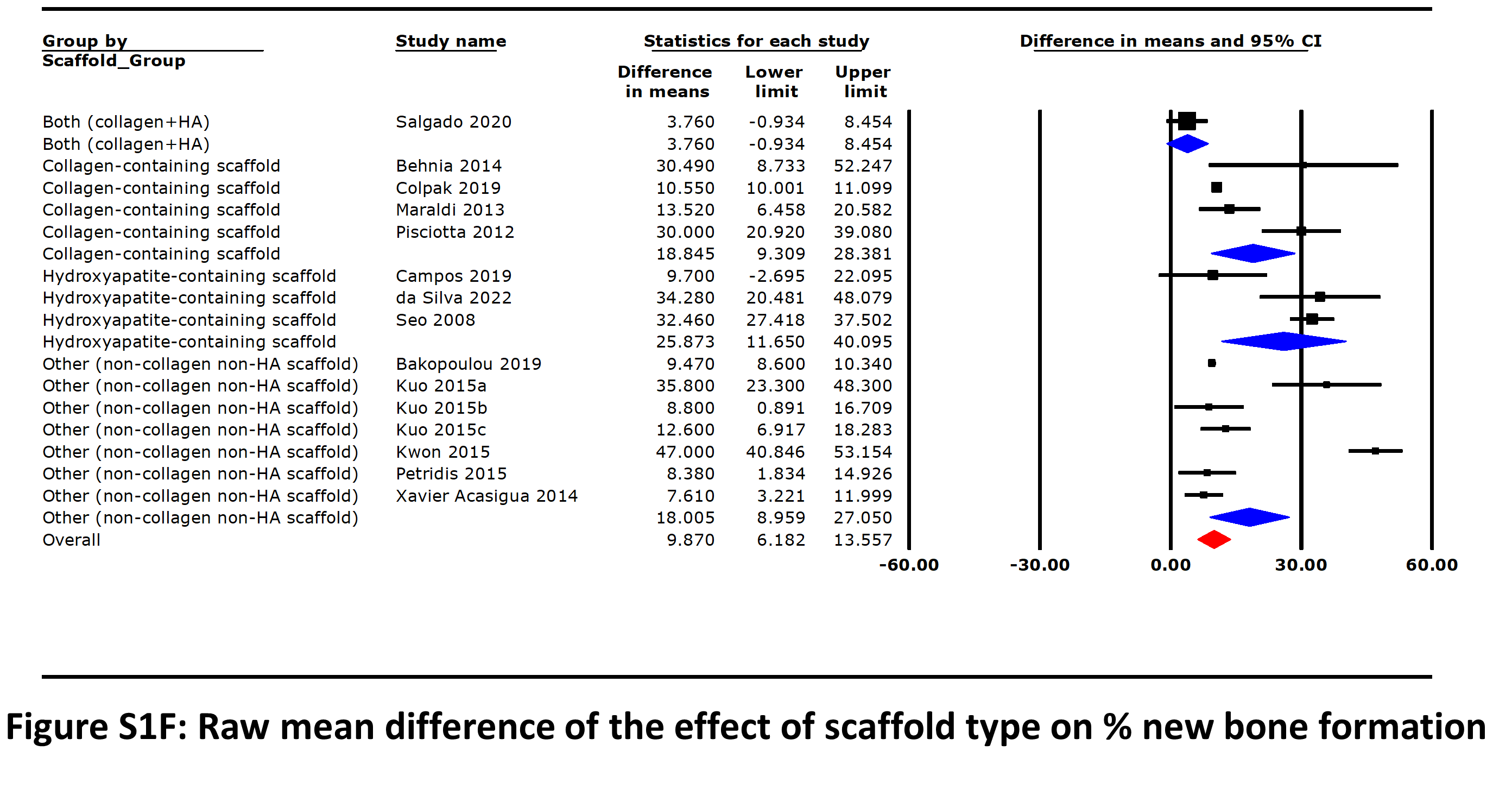

Supplement: Supplementary file 9 — Additional file 9. Raw mean difference of the effect of scaffold type on % new bone formation. [file 13287_2023_3357_MOESM9_ESM.tif]

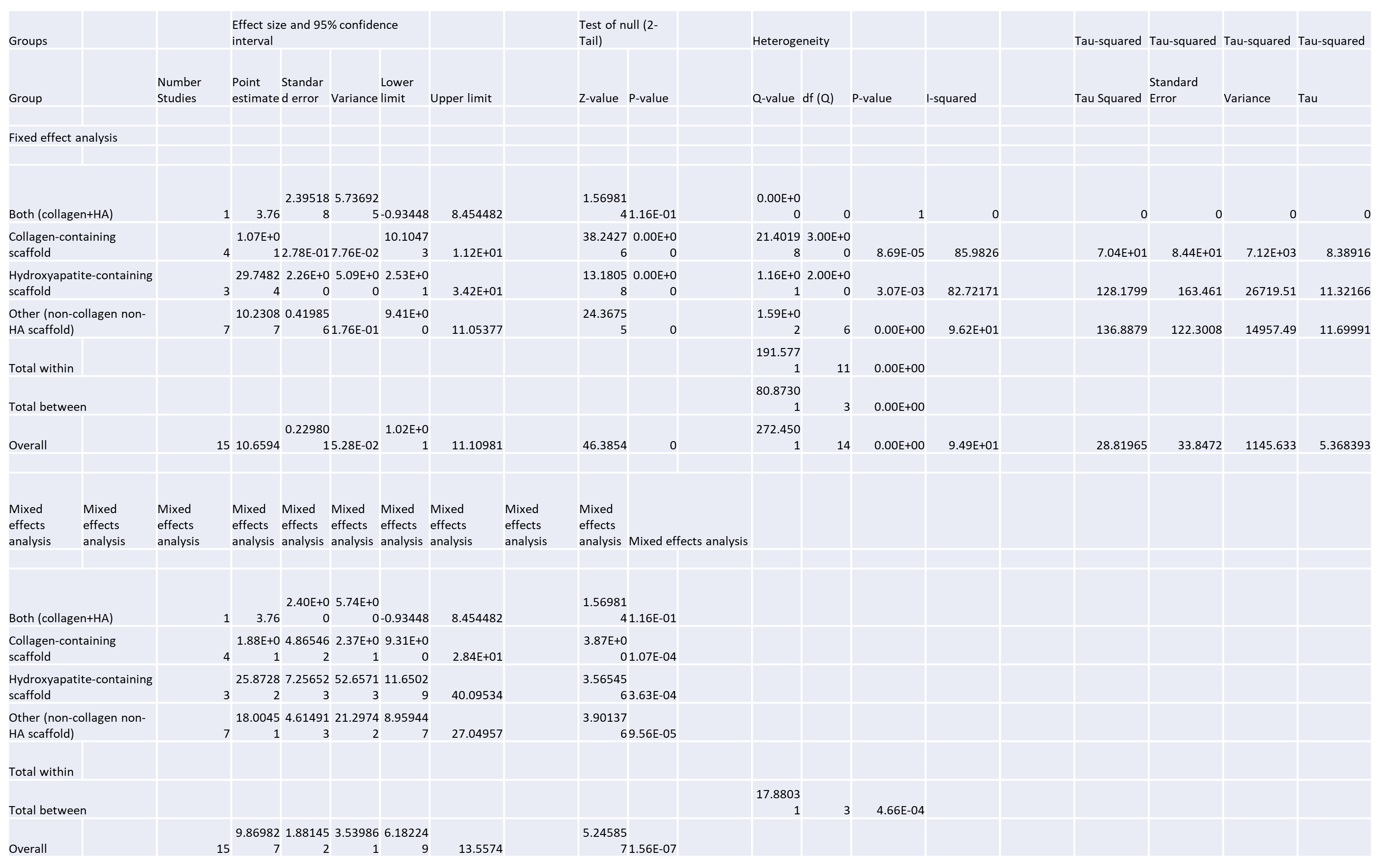

Supplement: Supplementary file 10 — Additional file 10. Detailed mean difference and the significane of the effect of scaffold type on % new bone formation. [file 13287_2023_3357_MOESM10_ESM.tif]

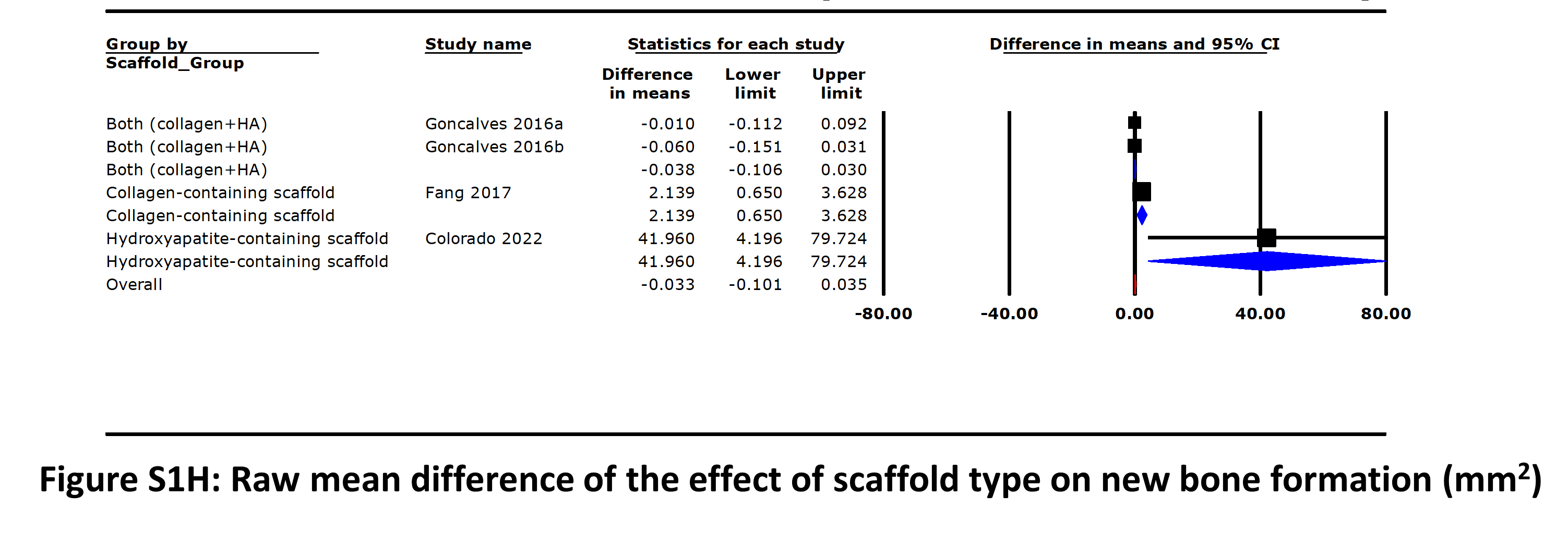

Supplement: Supplementary file 11 — Additional file 11. Raw mean difference of the effect of scaffold type on new bone formation (mm2). [file 13287_2023_3357_MOESM11_ESM.tif]

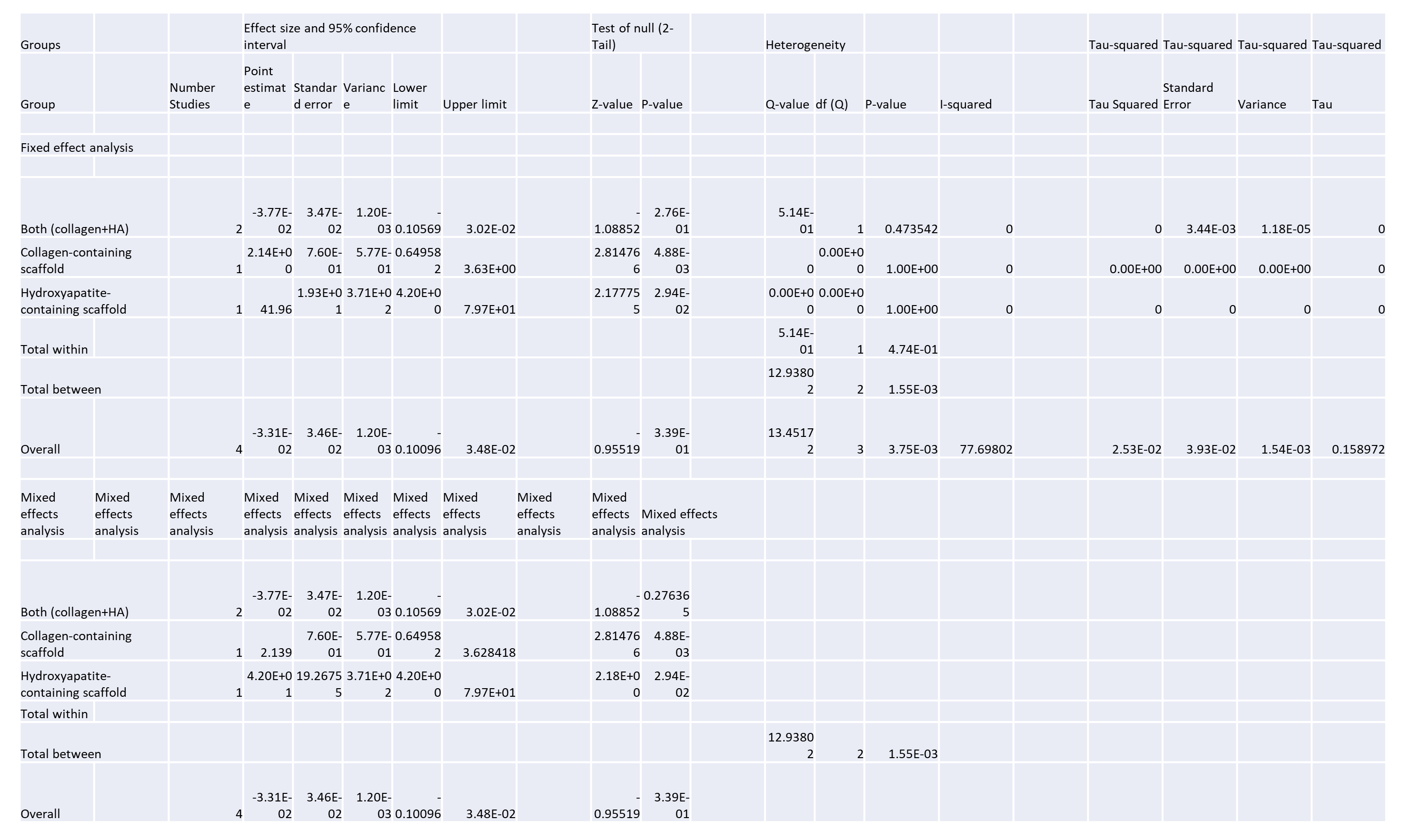

Supplement: Supplementary file 12 — Additional file 12. Detailed mean difference and the significance of the effect of scaffold type on new bone formation (mm2). [file 13287_2023_3357_MOESM12_ESM.tif]

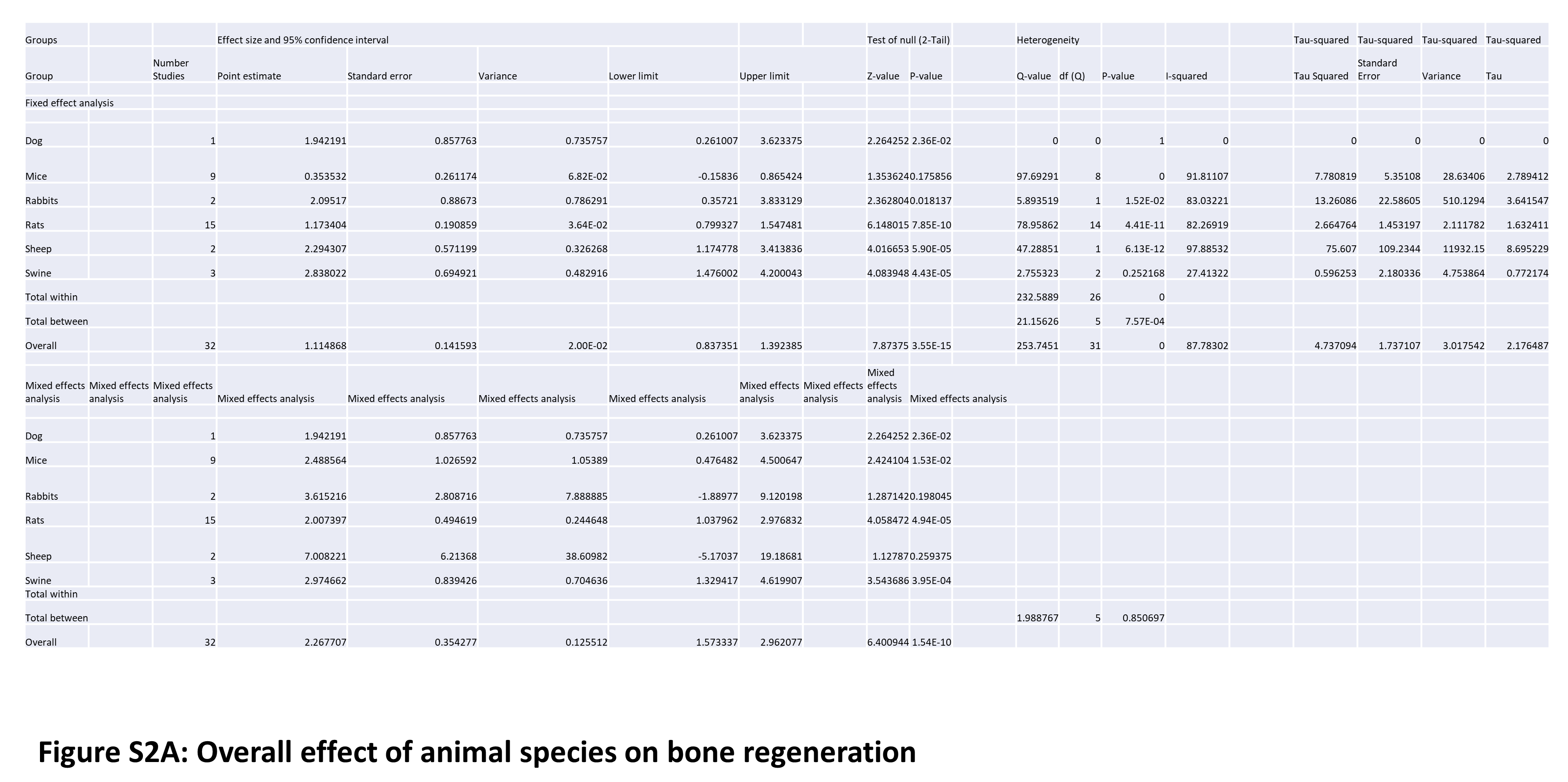

Supplement: Supplementary file 13 — Additional file 13. Overal effect of animal species on bone regeneration. [file 13287_2023_3357_MOESM13_ESM.tif]

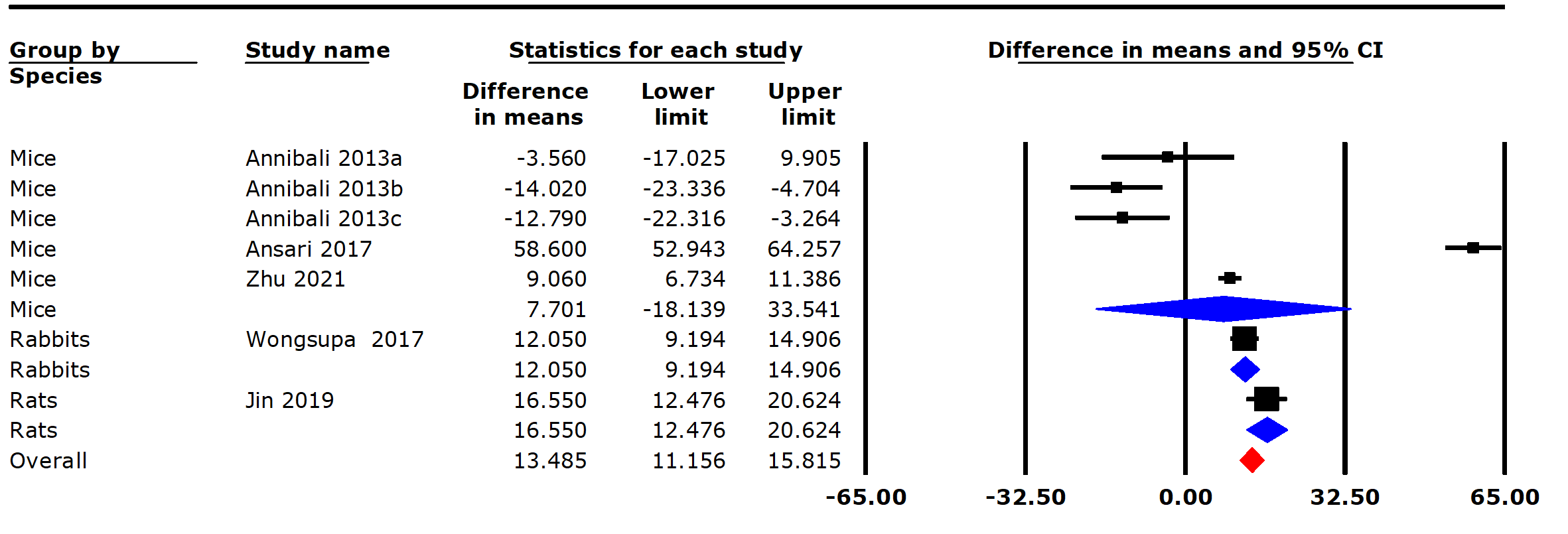

Supplement: Supplementary file 14 — Additional file 14. Raw mean difference of the effect of animal species on % BV/TV. [file 13287_2023_3357_MOESM14_ESM.tif]

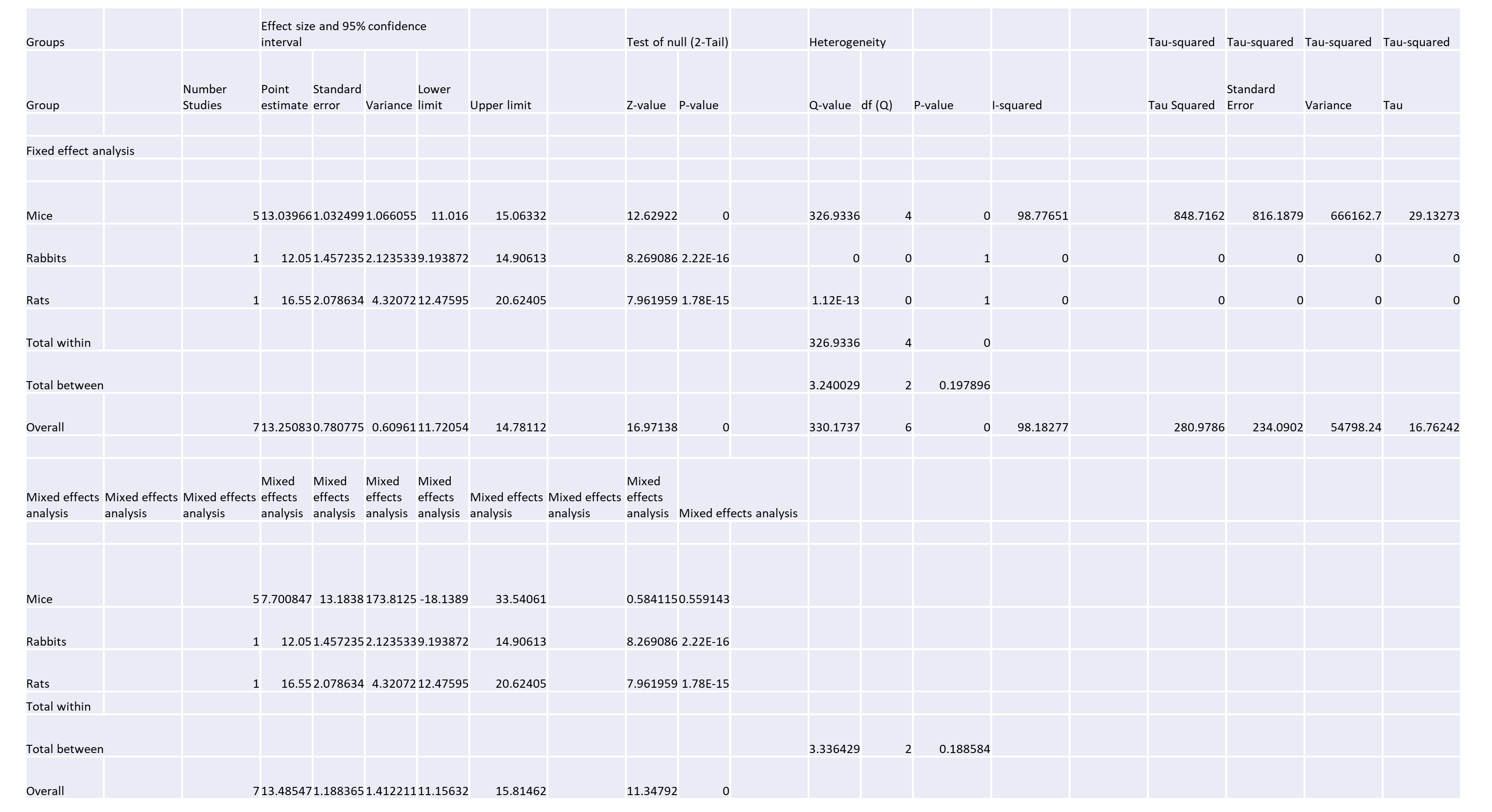

Supplement: Supplementary file 15 — Additional file 15. Detailed mean difference and the significance of the effect of species on % BV/TV. [file 13287_2023_3357_MOESM15_ESM.tif]

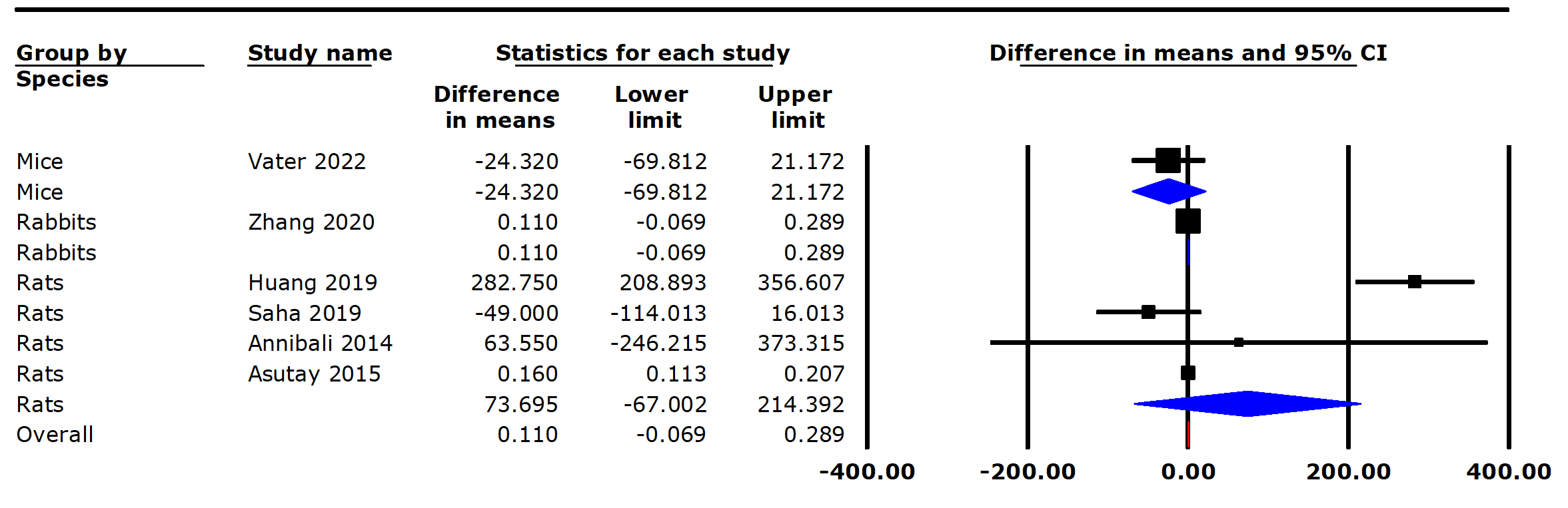

Supplement: Supplementary file 16 — Additional file 16. Raw mean difference of the effect of animal species on BMD. [file 13287_2023_3357_MOESM16_ESM.tif]

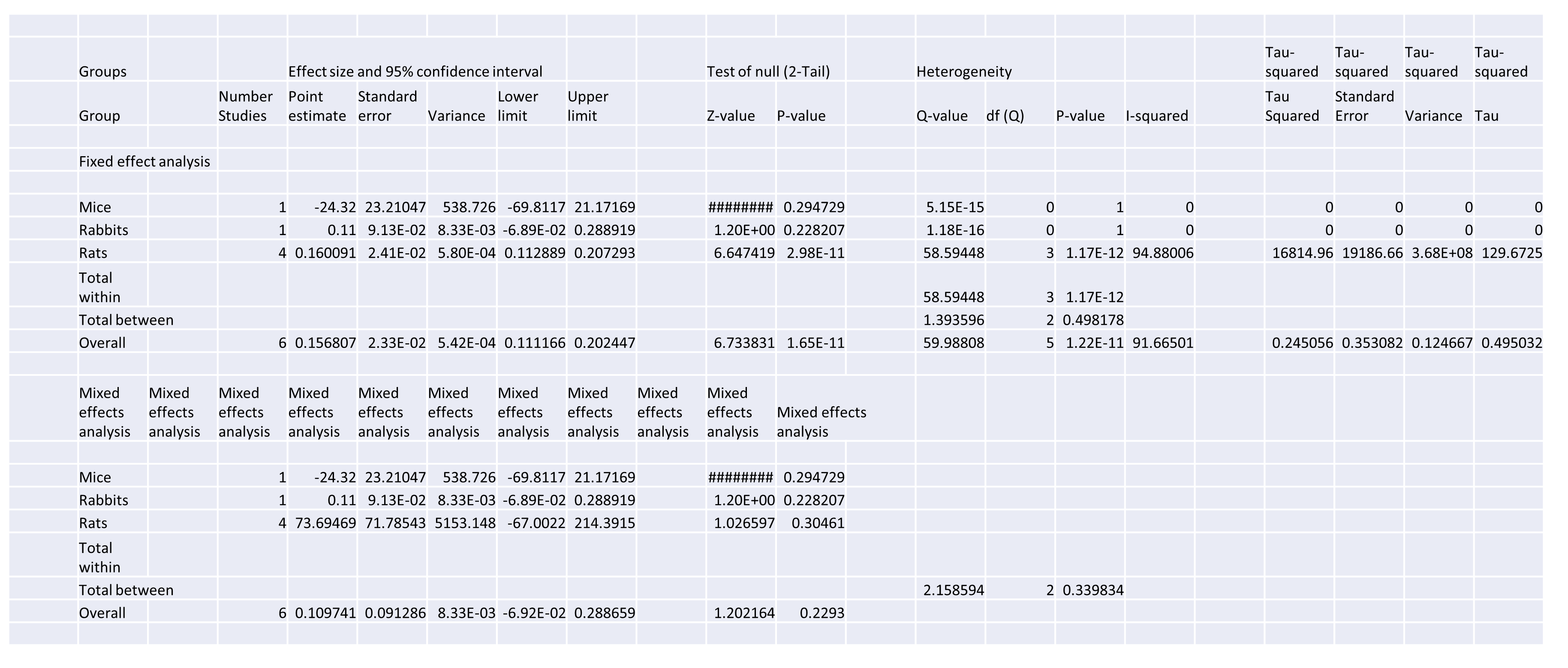

Supplement: Supplementary file 17 — Additional file 17. Detailed mean difference and the significance of the effect of animal species on BMD. [file 13287_2023_3357_MOESM17_ESM.tif]

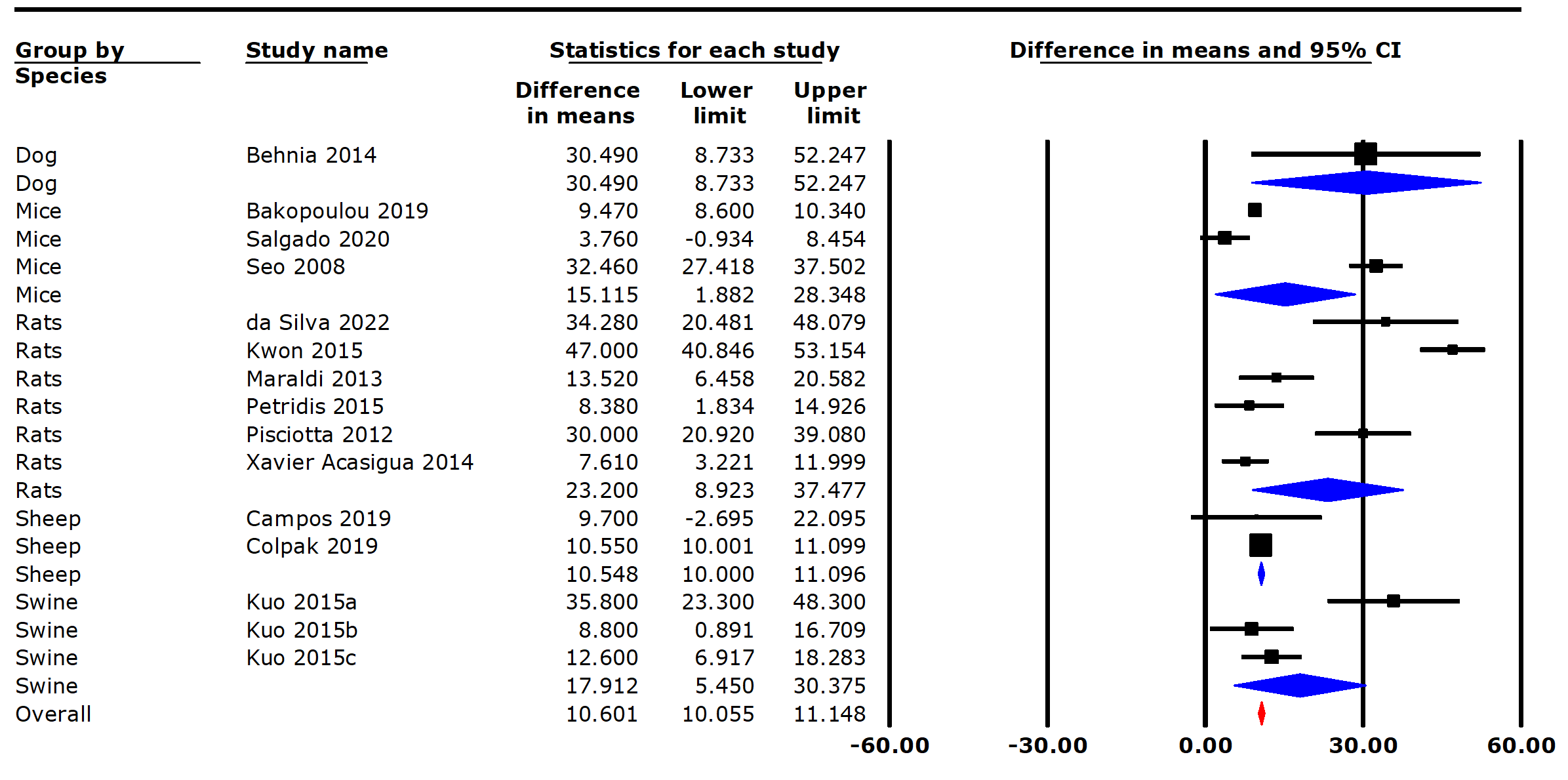

Supplement: Supplementary file 18 — Additional file 18. Raw mean difference of the effect of animal species on % new bone formation. [file 13287_2023_3357_MOESM18_ESM.tif]

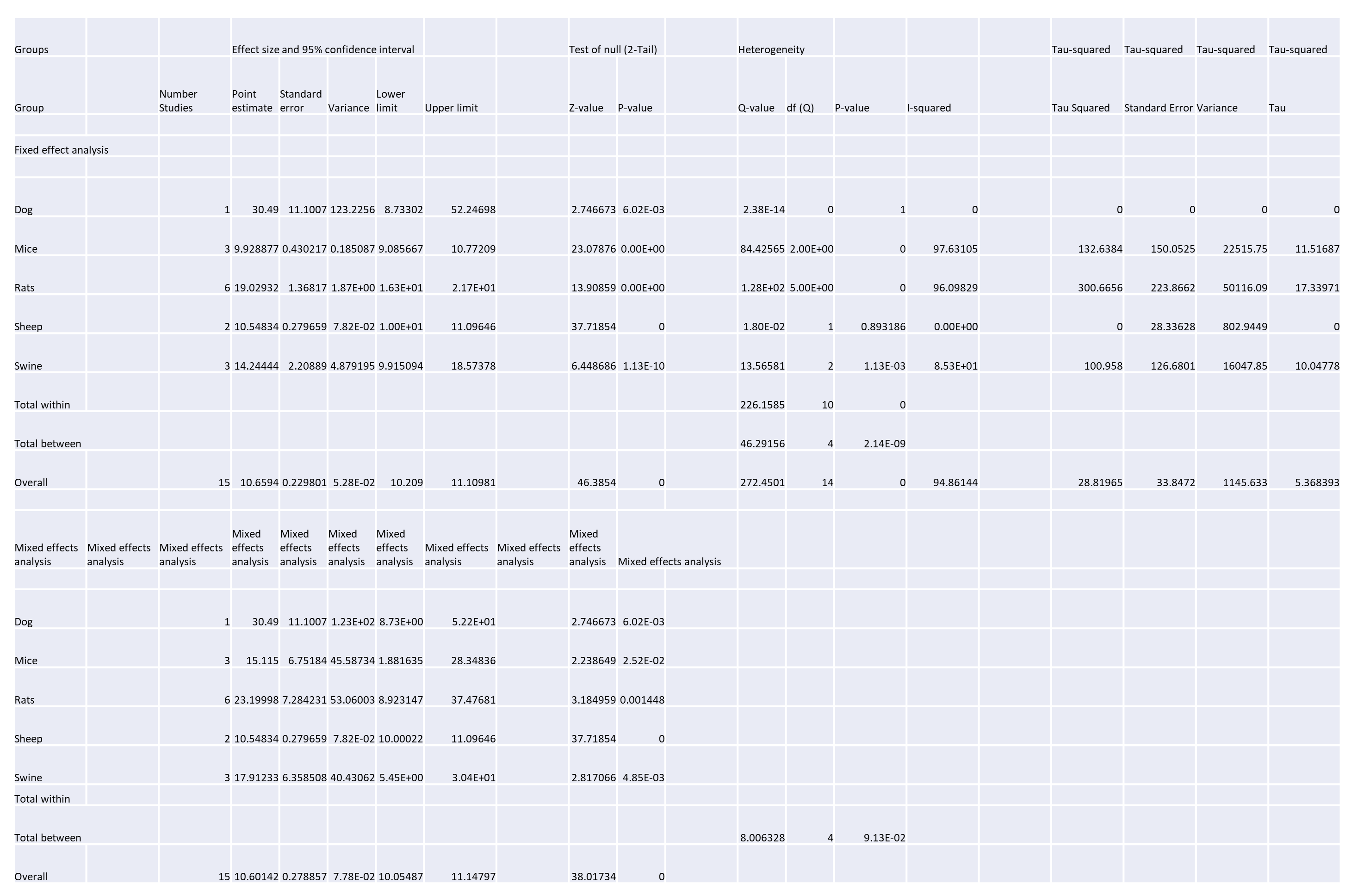

Supplement: Supplementary file 19 — Additional file 19. Detailed mean difference and the significance of the effect of animal species on % new bone formation. [file 13287_2023_3357_MOESM19_ESM.tif]

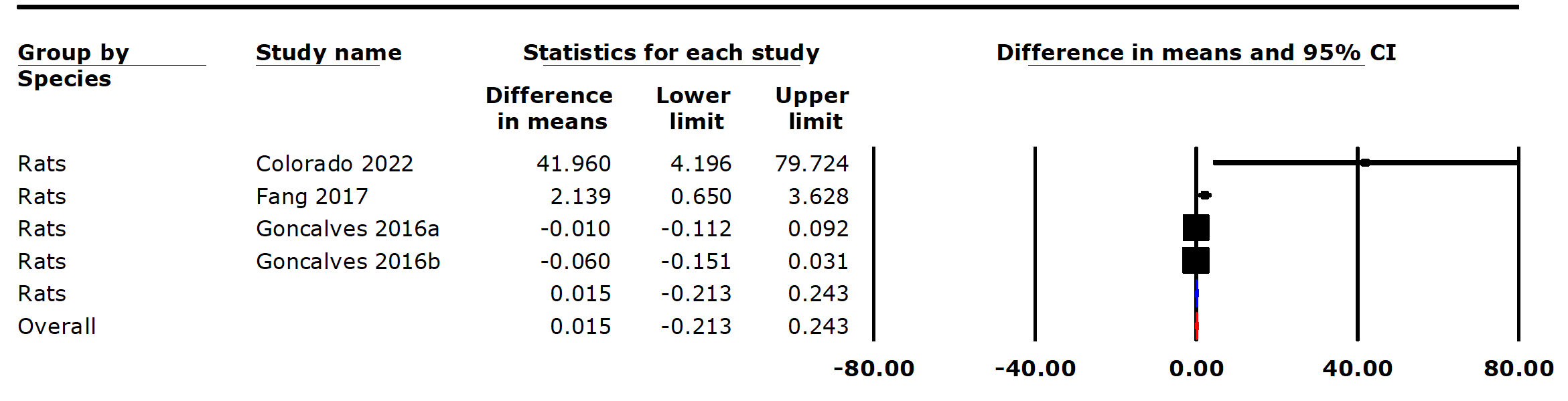

Supplement: Supplementary file 20 — Additional file 20. Raw mean difference of the effect of animal species on new bone formation (mm2). [file 13287_2023_3357_MOESM20_ESM.tif]

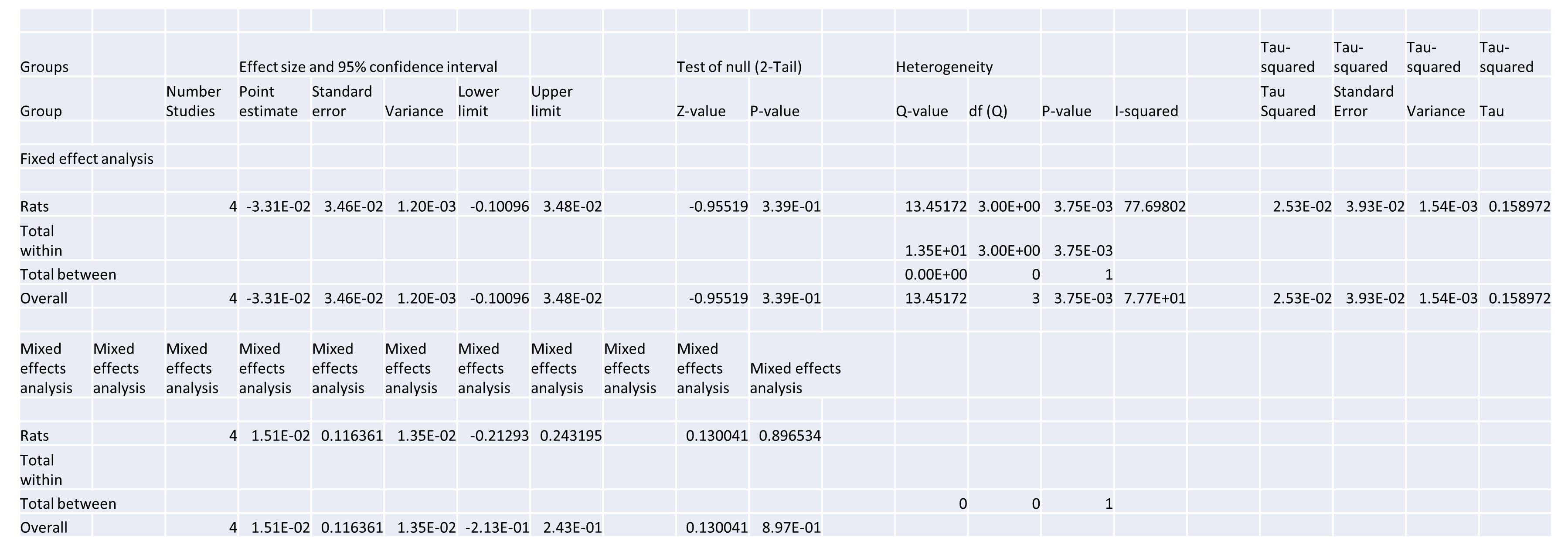

Supplement: Supplementary file 21 — Additional file 21. Detailed mean difference and the significane of the effect of species on new bone formation (mm2). [file 13287_2023_3357_MOESM21_ESM.tif]

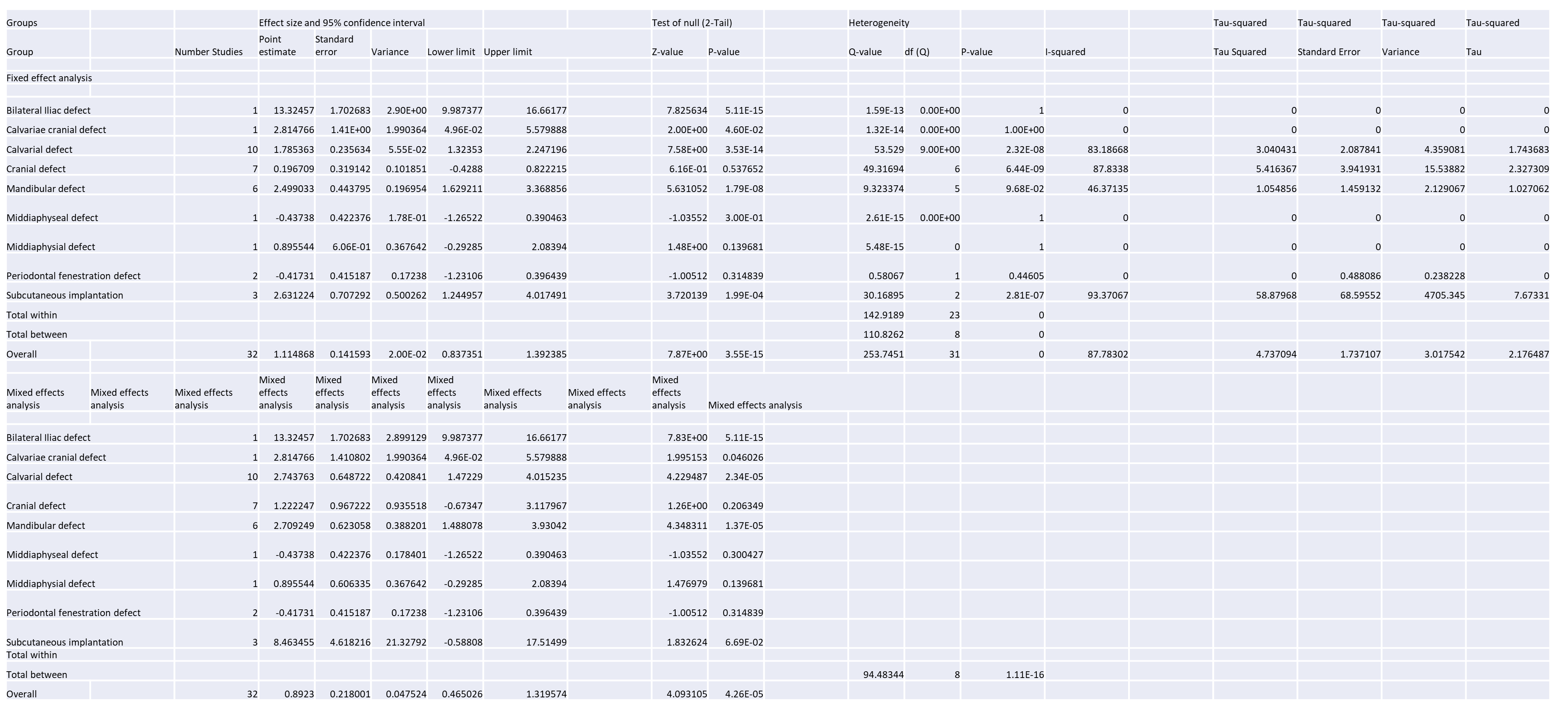

Supplement: Supplementary file 22 — Additional file 22. Overall effect of the site of defect in animals on bone regeneration. [file 13287_2023_3357_MOESM22_ESM.tif]

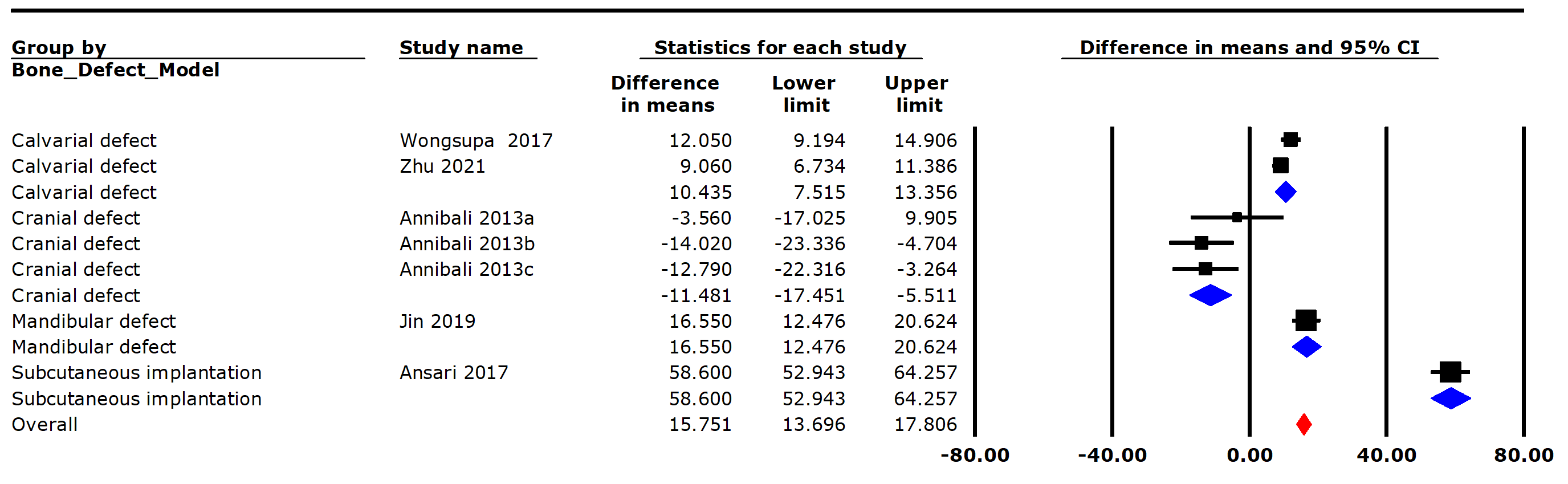

Supplement: Supplementary file 23 — Additional file 23. Raw mean difference of the effect of defect sites on % BV/TV. [file 13287_2023_3357_MOESM23_ESM.tif]

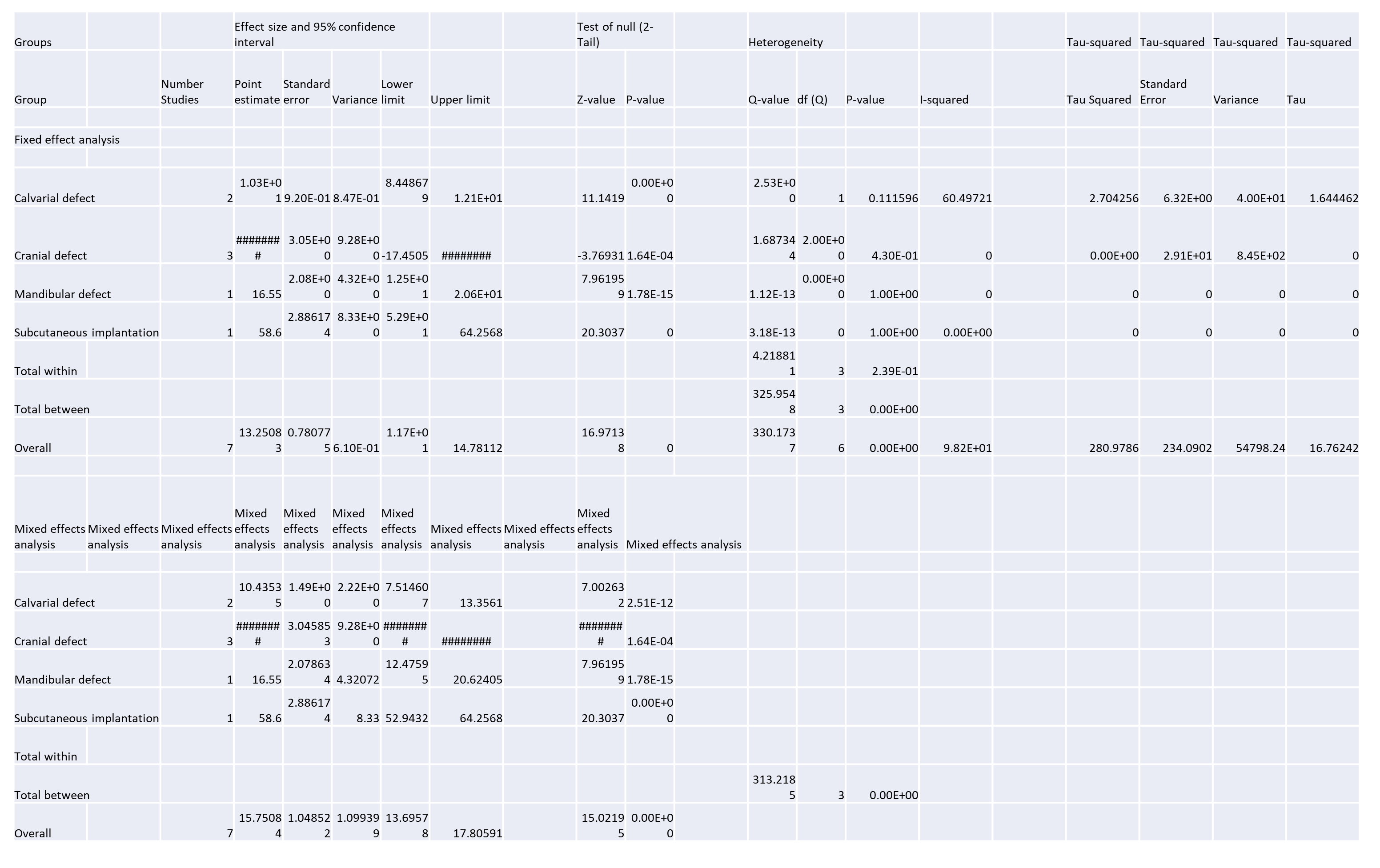

Supplement: Supplementary file 24 — Additional file 24. Detailed mean difference and the significance of the defect sites on % BV/TV. [file 13287_2023_3357_MOESM24_ESM.tif]

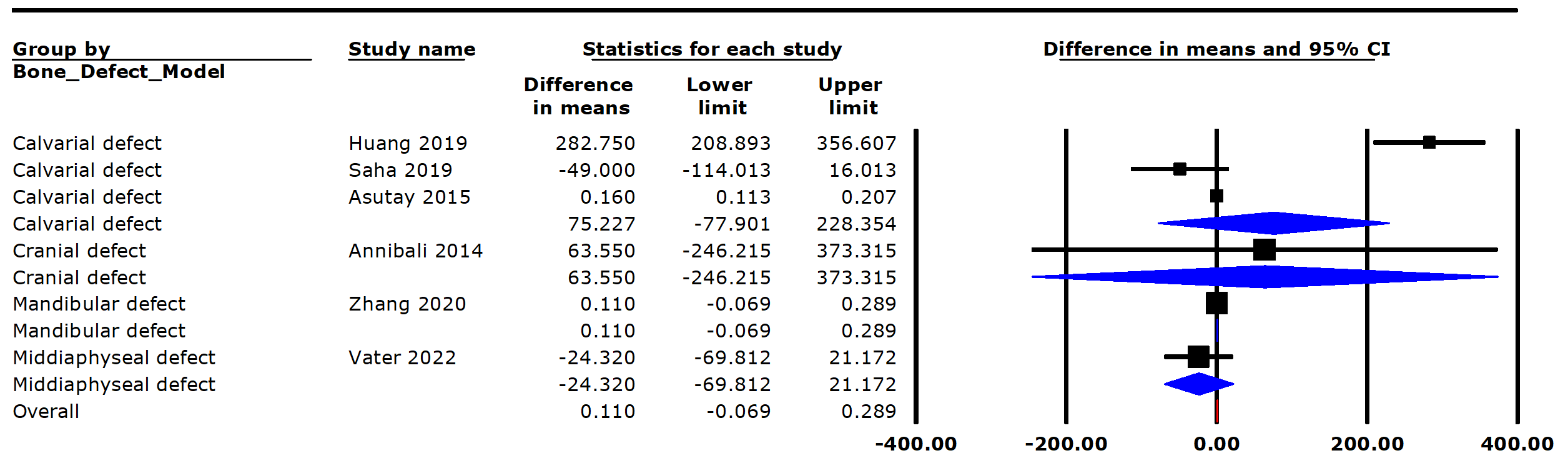

Supplement: Supplementary file 25 — Additional file 25. Raw mean difference of the effect of defect sites on BMD. [file 13287_2023_3357_MOESM25_ESM.tif]

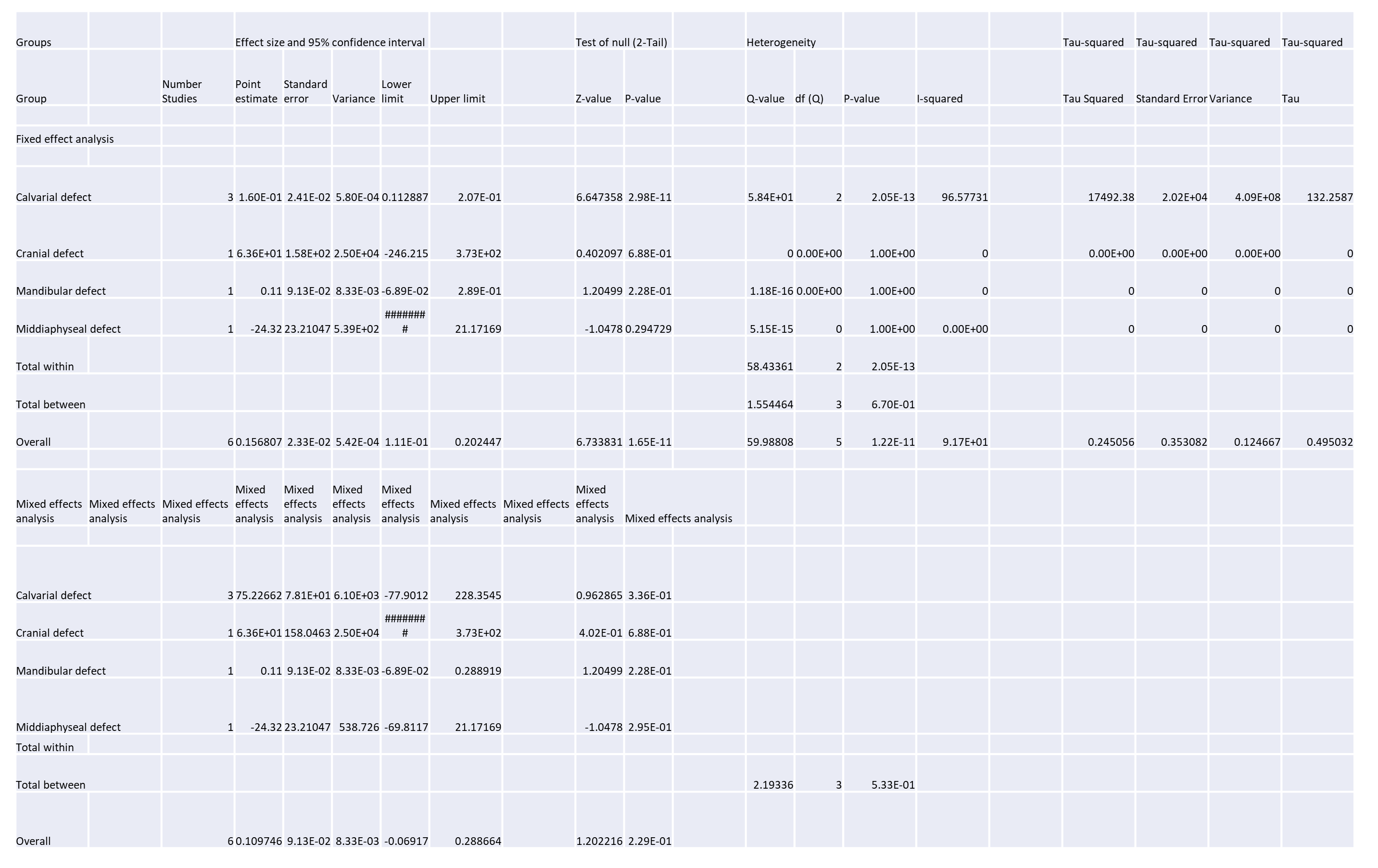

Supplement: Supplementary file 26 — Additional file 26. Detailed mean difference and the significance of the defect sites on BMD. [file 13287_2023_3357_MOESM26_ESM.tif]

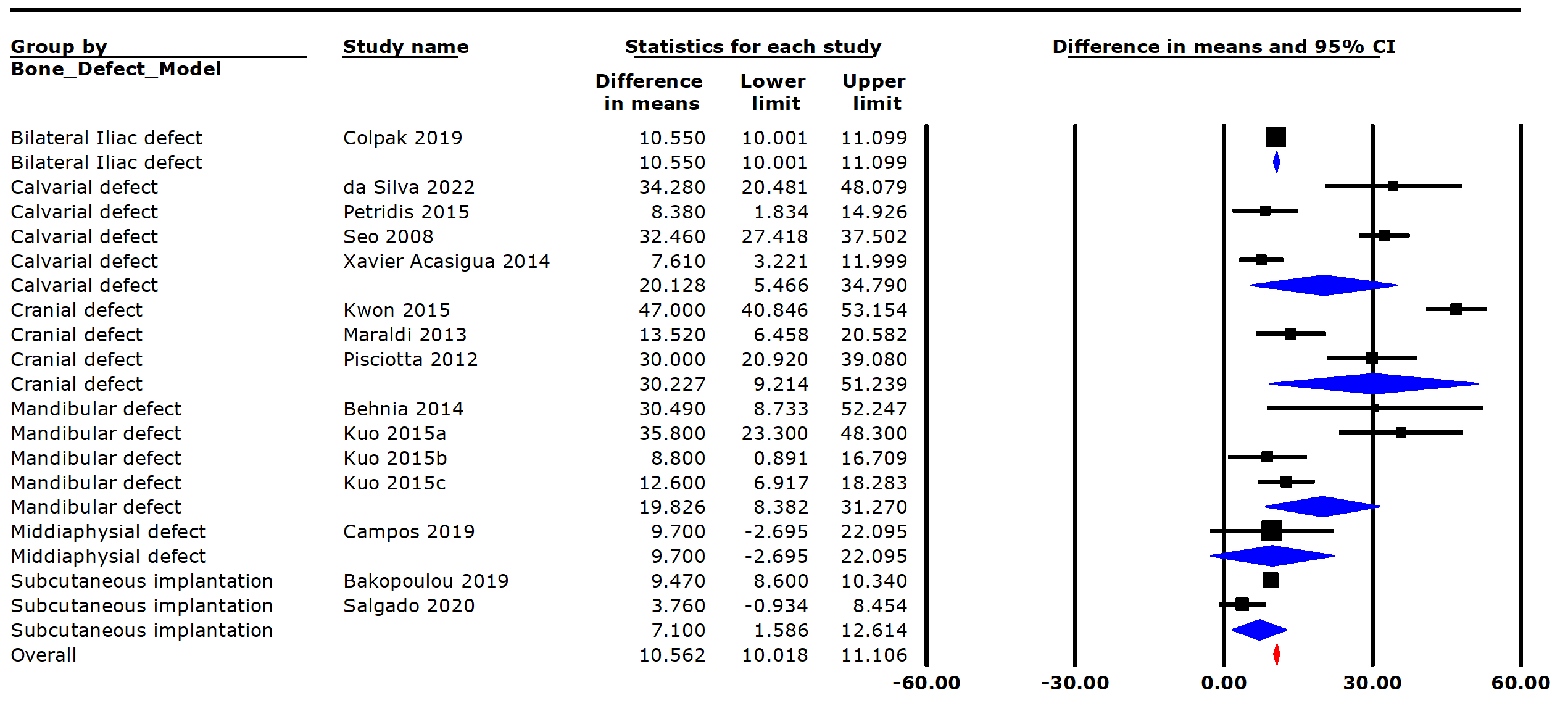

Supplement: Supplementary file 27 — Additional file 27. Raw mean difference of the effect of defect sites on % new bone formation. [file 13287_2023_3357_MOESM27_ESM.tif]

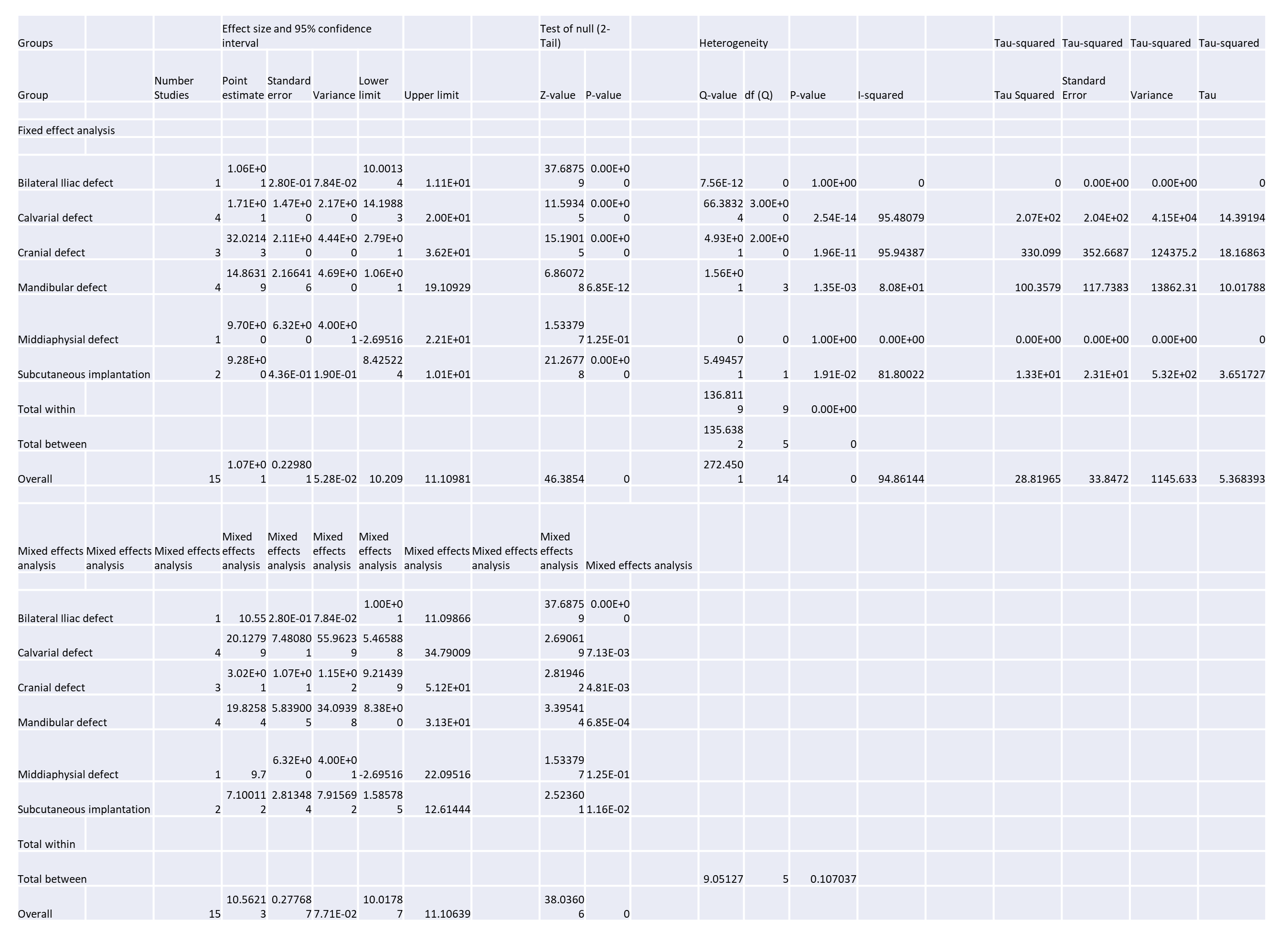

Supplement: Supplementary file 28 — Additional file 28. Detailed mean difference and the significance of the defect sites on % new bone formation. [file 13287_2023_3357_MOESM28_ESM.tif]

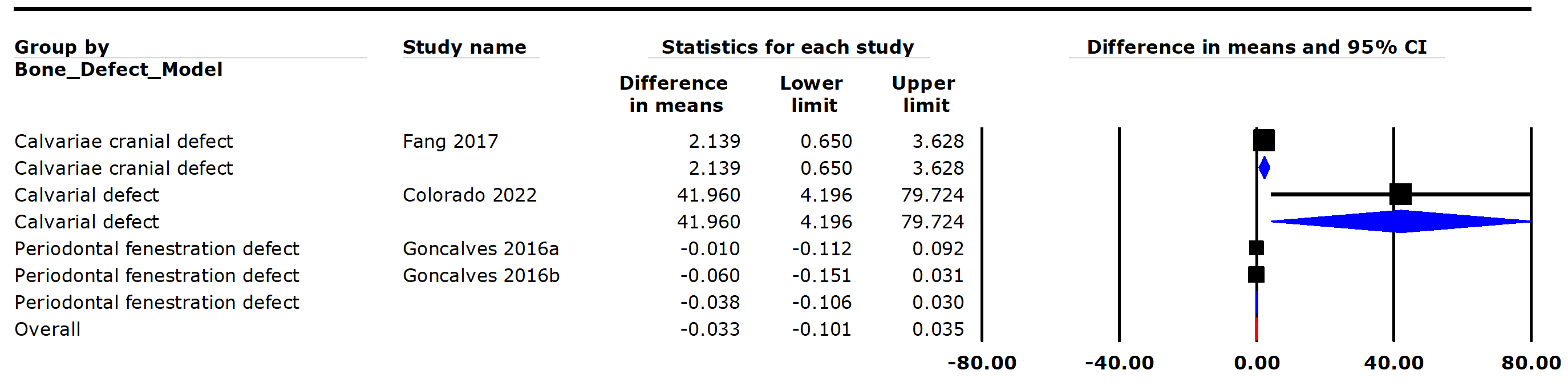

Supplement: Supplementary file 29 — Additional file 29. Raw mean difference of the effect of defect sites on new bone formation (mm2). [file 13287_2023_3357_MOESM29_ESM.tif]

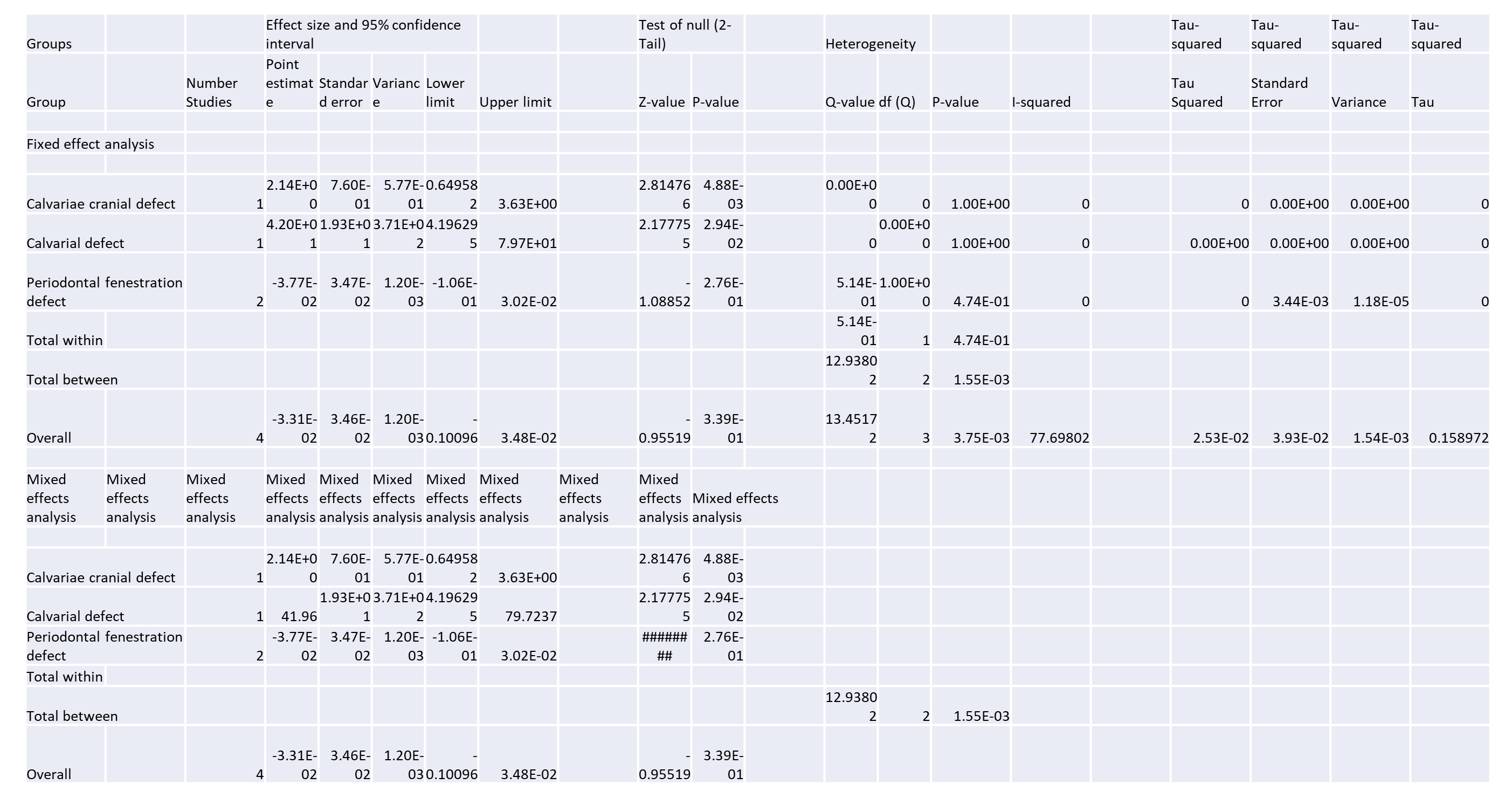

Supplement: Supplementary file 30 — Additional file 30. Detailed mean difference and the significance of the defect sites on new bone formation (mm2). [file 13287_2023_3357_MOESM30_ESM.tif]

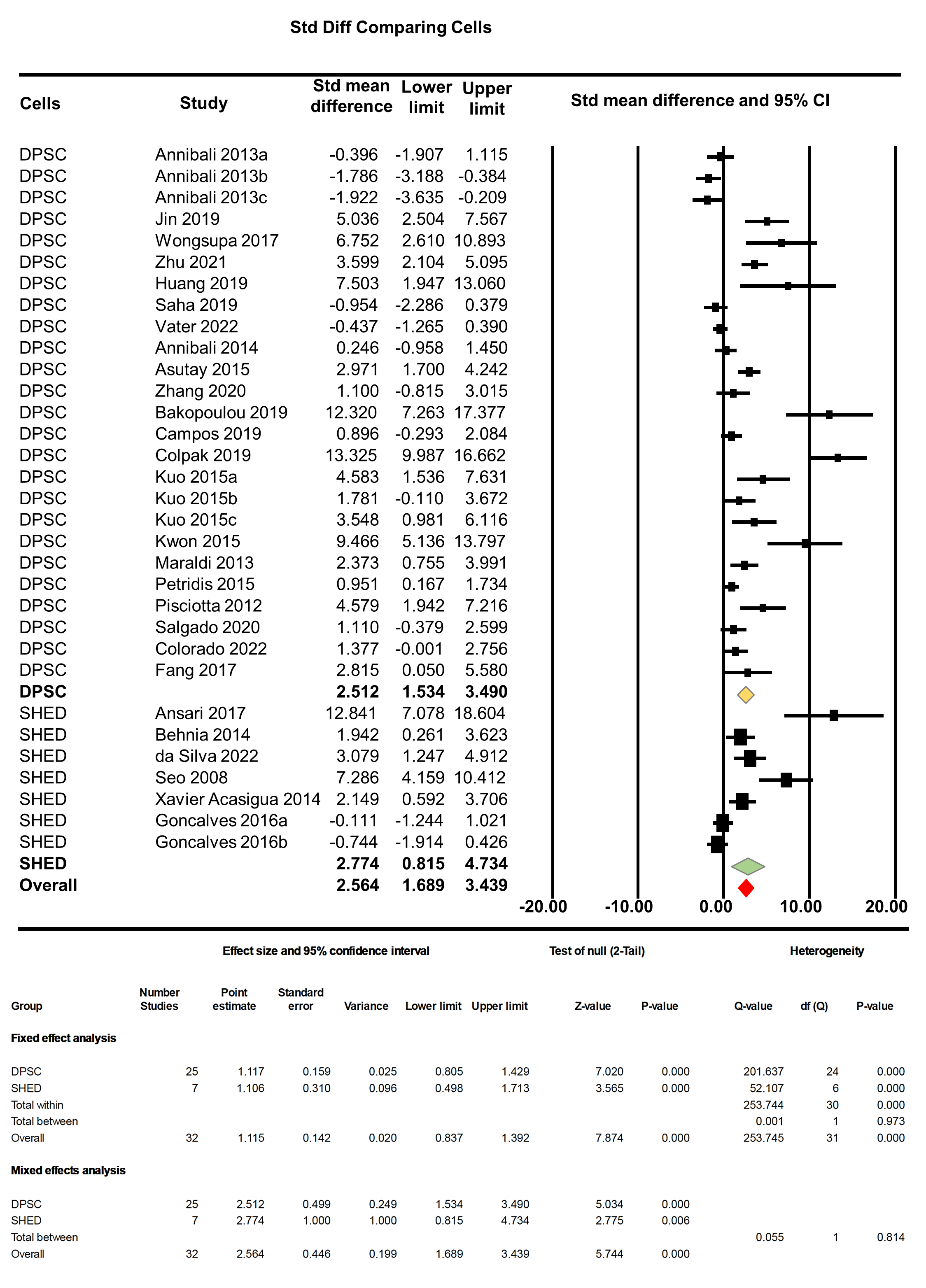

Supplement: Supplementary file 31 — Additional file 31. Overall effect of the type of dental pulp stem cells on bone regeneration. [file 13287_2023_3357_MOESM31_ESM.tif]
